# Evaluating single-cell ATAC-seq atlasing technologies using sequence-to-function modeling

**DOI:** 10.1038/s41467-026-68742-4

**Published:** 2026-01-22

**Authors:** Hannah Dickmänken, Marta Wojno, Lukas Mahieu, Koen Theunis, Eren Can Ekşi, Valerie Christiaens, Niklas Kempynck, Florian V. De Rop, Natalie Roels, Katina I. Spanier, Roel Vandepoel, Gert Hulselmans, Suresh Poovathingal, Stein Aerts

**Affiliations:** 1 Laboratory of Computational Biology, VIB Center for AI & Computational Biology, Leuven, Belgium; 2 VIB-KU Leuven Center for Brain & Disease Research, Leuven, Belgium; 3 Department of Human Genetics, KU Leuven, Leuven, Belgium; 4 VIB-KU Leuven Center for Brain & Disease Research, CBD Technologies, Single Cell & Microfluidics Expertise Unit, Leuven, Belgium; 5 Aligning Science Across Parkinson’s (ASAP) Collaborative Research Network, Chevy Chase, MD 20815 USA

**Keywords:** Data acquisition, Embryogenesis, Machine learning, DNA, Computational models

## Abstract

Deciphering the cis-regulatory logic underlying cell type identity remains a key challenge in biology. Single-cell chromatin accessibility (scATAC-seq) atlases enable training of sequence-to-function (S2F) deep learning models to decode enhancer logic. Yet, optimal criteria for constructing training datasets, i.e., the number of cells and ATAC fragments, remain unclear. Moreover, the suitability of different scATAC-seq platforms for such models has not been systematically tested. We introduce HyDrop v2, an improved custom droplet scATAC-seq method, and perform the first benchmark of scATAC-seq platforms focusing on its capacity to train S2F models and its capacity to yield TF footprints in different species. We show that lower fragment counts can be compensated for by increased cell numbers. S2F models trained on custom or commercial data perform comparably in enhancer prediction, sequence explainability, and transcription factor footprinting. We demonstrate that integrating data from different scATAC-seq platforms enables large-scale, cost-efficient atlas construction for deep learning-based regulatory modeling.

## Abstract (web summary)

Generating high-quality training data for machine learning is costly. Here, authors include sequence-to-function modeling in benchmarking of custom and commercial droplet-based scATAC platforms, and release a new Drosophila embryo atlas along with a new mouse cortex atlas, assessed for model interpretability.

## Introduction

Cell type identity is encoded in the DNA sequence of *cis*-regulatory elements (CREs), such as enhancers^1^. By harboring binding sites for specific combinations of transcription factors (TFs), CREs orchestrate cell-type-specific binding of TFs, subsequently resulting in the expression of genes underlying the cell’s identity^1^. Thus, identifying CREs is a fundamental question in biology. Several experimental approaches are available to identify CREs and characterize their function^2–4^. Measuring chromatin accessibility with Assay of Transposase Accessible Chromatin (ATAC) using the enzyme Tn5 to tag open chromatin offers an unbiased approach that can be performed in single cells (scATAC)^2,4^. After clustering cells, profiled with scATAC-seq, into distinct cell types or cell states, differential accessibility between cell types was found as an accurate enhancer-predictor, thus far^5–10^.

Over recent years, various scATAC-seq methods have been developed using either well-based combinatorial indexing strategies or microfluidics encapsulation (for an in-depth comparison, see De Rop et al.^9^). To date, benchmarking and evaluation of scATAC-seq data quality have been performed with metrics such as fragment count, cell type clustering, integrability with other datasets, and the power to identify differentially accessible regions (DARs)^9^.

The rapid growth of machine learning, especially deep learning (DL), has opened new possibilities for accurately identifying promoters and enhancers, and deciphering the combinatorial rules of TF binding sites (TFBS) within regulatory regions. So-called sequence-to-function (S2F) models facilitate this decoding step. Even though they can be trained on different epigenomic datasets^5^, S2F models trained only on scATAC atlases^5,10–12^ have been shown to be excellent oracles for deciphering the cis-regulatory code and for designing synthetic enhancers (for an in-depth review, see De Winter et al., 2025^8^). A recent benchmarking study on various data modalities showed that training S2F models on scATAC data performs superior in predicting cell-type-specific enhancers compared to non-deep learning methods or using other data modalities^5^. Importantly, the need for high-quality scATAC data with improved coverage is emphasized^5^. Earlier work has trained multi-class S2F models with as few as 100-200 cells for several cell types^11^, while alternative methods like ChromBPNet have also shown that a minimum of (pseudobulked) coverage is needed^13^ (for example, 5 million reads in total, cumulative across all pseudobulked cells). The minimal quality, number of cells, and read depth needed to train S2F models have, however, not yet been specified. Additionally, recently developed S2F models assume that the preferential binding of the Tn5 enzyme to specific base pairs, i.e., Tn5 bias, does not influence model performance^14^ or that Tn5 bias can be corrected to identify TF footprints^13,15^. The effect of different protocols and sequencing platforms on Tn5 tagmentation has, however, not yet been determined. Such a quantitative evaluation of scATAC-seq techniques becomes even more relevant as more ready-to-use DL packages are developed, making S2F modeling available to a broad research audience^13,14,16^.

The combination of the great demand for training data for S2F models^5,12^ and the sparse nature of scATAC data^2^ poses challenges to developing organism-scale S2F models^5,12^. Beyond comparing the quality and costs of various scATAC-seq techniques^9^, to our knowledge, no benchmarking has been performed to assess the effect of different scATAC-seq techniques (e.g., commercial versus custom platforms) on the capacity to train S2F models.

In an earlier publication, we developed HyDrop, a microfluidics droplet technology based on deformable hydrogel indexing beads, which enables high-throughput profiling of both single-cell RNA-seq and single-nuclei ATAC-seq^17^. Based on this work, we have improved several aspects of the technology to develop HyDrop-ATAC v2 (further addressed as HyDrop v2), which enables profiling of single-cell ATAC-seq with higher detection sensitivity compared to v1. Like commercial platforms for droplet-based scATAC-seq (e.g., 10X Genomics Epi ATAC), HyDrop v2 uses a hydrogel-based single-cell indexing strategy with droplet microfluidics. Alternative commercial platforms are based on microwell encapsulation of single cells (BD Rhapsody) or use a well-based combinatorial indexing strategy (Parse Bioscience). Overall, the commercial platforms are costly and/or require labor-intensive workflows that hamper the generation of large-scale single-cell atlasing works of complex tissues or entire organisms^9,18^.

Here, we perform the first benchmark of a scATAC-seq platform focusing on its capacity to train S2F models and its capacity to yield TF footprints in different species and complex tissues. We compare the performance of S2F models trained on different read depths and numbers of cells, highlighting model improvements upon increasing the size of the training dataset.

By offering highly accessible data generation with the open-source HyDrop v2, we contribute to unraveling cell-type-specific regulatory elements in health and disease with DL. Our approach of including S2F modeling into the benchmarking of custom data generation platforms paves the way for a synergy between computational and experimental disciplines in method development.

## Results

### S2F training data generation using commercial and custom technologies

**Fig. 1:**
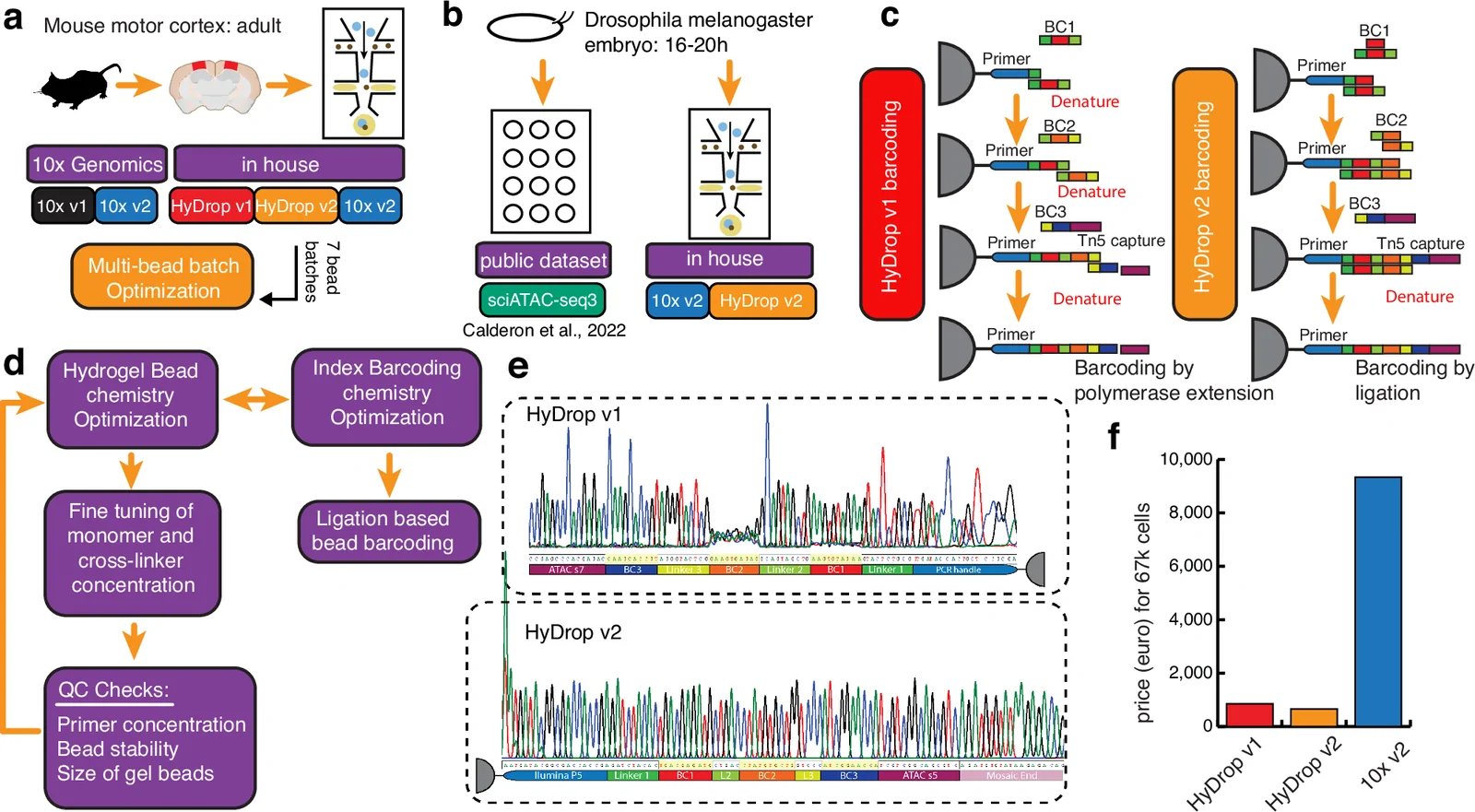
scATAC-seq atlasing with commercial and improved custom technology. **a** Mouse cortex data were partly downloaded from the official 10x Genomics website and partly generated in-house. Overview of the sample generation: dissection of mouse motor cortex, tissue lysis, followed by droplet encapsulation for cell barcoding. For HyDrop v1, only one bead batch was generated (data reused from De Rop et al., 2022), while for HyDrop v2, seven bead batches were generated. **b** *Drosophila melanogaster* embryos were collected 16-20 h after egg laying. In-house experiments were performed with 10x v2 chemistry on the 10x Chromium device, while HyDrop v2 beads were used for the open-source data generation. sciATAC-seq3 data (plate-based essay) were retrieved from Calderon et al. (2022). **c** Comparison between bead barcoding chemistries of HyDrop v1 and v2. **d** The HyDrop v1 protocol was optimized in two ways: (1) the barcoding of the beads was changed from a cyclic polymerase extension to a linear ligation-based chemistry (right), and (2) Bead chemistry was adjusted in several optimization rounds (left). **e** Sanger sequencing of an exemplary HyDrop v1 bead (top) showing impurity in barcode one and barcode two, and a HyDrop v2 bead (bottom) with pure barcode signals across all three barcodes. **f** Estimation of generation costs of 67k cells in euros, excluding sequencing costs. Source data are provided as a Source Data file. HyDrop v1: 851.53 euro, HyDrop v2: 668.18 euro, 10x v2: 9,337.79 euro.

To compare the quality of different scATAC-seq training data sets for S2F modeling, we generated two atlases: the mouse brain (M1 primary motor cortex *Mus musculus*) and the fly embryo (*Drosophila melanogaster*). For both, we generated scATAC-seq atlases using the commercial 10x Genomics kit and the custom droplet microfluidics technique HyDrop v2^17^ (Fig. 1a, b). An earlier benchmarking study has shown that the original HyDrop method suffered from a lower sensitivity (measured as the fraction of uniquely mapped reads per cell), as well as higher variability between bead batches, compared to 10x Genomics^9^. Details on the technical improvements of the HyDrop v2 scATAC-seq platform can be found in the supplementary note.

In short, we modified the bead barcoding process into three stages through ligation (Fig. 1c) and adapted the bead chemistry (Fig. 1d). Single bead picking, followed by Sanger sequencing, confirmed increased purity of individual barcodes (Fig. 1e, Fig. S1a), increasing the sensitivity. The modifications with polymer chemistry resulted in high-quality bead batches and enhanced freeze-thaw stability (Fig. S1b). These changes in bead generation were validated through multiple batches of beads that were produced across seven independent bead production runs (Fig. S1c).

Next, we generated the mouse atlas spanning 67k cells collected across seven bead batches. The cost to generate a dataset of 67k cells using HyDrop v2 is estimated at 668 euros (excluding sequencing), which is an improvement compared to HyDrop v1 (852 euros), and 14 times cheaper than 10x (9338 euros, Fig. 1f). A detailed overview of the costs can be found in Supplementary Tables S4 and S5.

When combining the data of 35 separate HyDrop v2 experiments on frozen adult mouse cortex into one dataset, no batch correction is needed (Fig. S1g). For downstream analysis, the HyDrop v2-based data integrates seamlessly with 10x (v1 and v2) and HyDrop v1 data (Fig. S1h).

In both the mouse motor cortex and the *Drosophila* embryo, we uncover all major cell types using the HyDrop v2 atlas (Tables S1, S3). Compared to the previously published HyDrop v1 atlas of the mouse cortex, we observe a 9.14% increase in the recovered unique fragments (log) at 36k reads per cell (kRPC, Fig. S2a). In the fly embryo atlas, HyDrop v2-based data reaches an average of 94.1% of the unique fragments (log) count, compared to 10x v2-based data at 12kRPC (Fig. S2b). Like with HyDrop v1, the barnyard of mouse and human cell lines generated with HyDrop v2 separates well (Fig. S2c).

To compare cell-type features in-depth, we calculated DARs of each cell type, per technique. DARs identified by HyDrop v2-based data largely overlap with 10x (v2)-based DARs in mouse (Fig. S2d, e) and fly (Fig. S2f, g). Zooming in on the genome-wide coverage, HyDrop v2-based DARs are similar to the 10x (v2)-based coverage, while HyDrop v1 DARs tend to cover smaller regions in the mouse (Fig. S2e). In fly, sciATAC-based DARs cover very large regions while HyDrop v2- and 10x v2-based DARs are more specific (Fig. S2g).

Next, we asked whether these DARs capture similar biological information between techniques. Applying motif enrichment analysis (pyCisTarget^7^), the recovered motifs of HyDrop v2-and 10x-based data correlate highly on their normalized enrichment score (NES) per cell type in mouse (*r* = 0.89, Fig. S2h) and fly (*r* = 0.95, Fig. S2i).

### Increased cell count improves S2F model performance

While enrichment analysis only uncovers known patterns of TF binding sites, i.e., motifs^7^, sequence-based deep learning models trained on scATAC data have been shown to identify potential enhancers and understand the grammar of cell-type-specific regulatory sequences^5,10,12^. Published models thus far have been trained on data generated by commercial platforms such as 10x Genomics^5,10,11^. To advance the to-date benchmarking methods beyond quality control metrics, we aimed to investigate the potential of HyDrop v2-based data as S2F model training data using the CREsted package^14^.

**Fig. 2:**
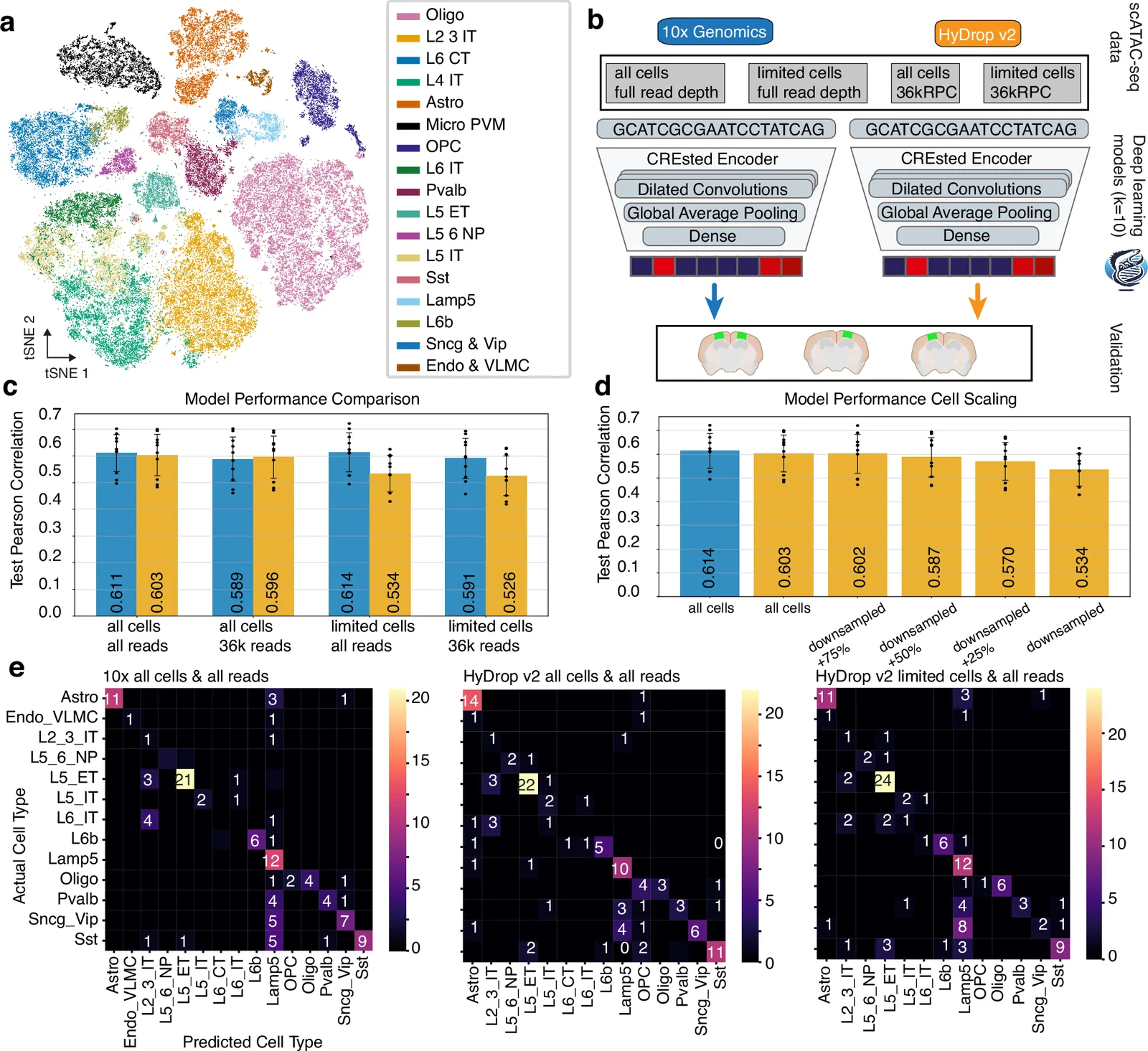
S2F models trained on atlases with varying cell count and read depth. **a** *t*-distributed stochastic neighbor embedding (tSNE) of all 92,363 cells across 44 experiments (2 experiments 10x v1 with a total of 9756 cells, 2 experiments 10x v2 with a total of 9722 cells, 5 experiments HyDrop v1 with a total of 5805 cells, 35 experiments HyDrop v2 with a total of 67,080 cells) colored by cell type, batch corrected for the used technique. Data points were randomly shuffled before plotting. **b** Computational design: 10x v1 and v2 datasets were combined into the 10x Genomics training dataset to compare to the HyDrop v2-based dataset, serving as training data for S2F deep learning models in k-fold cross-validation (*k* = 10). The model performance is validated on standard DL metrics, accessibility predictions, and mouse cortex enhancers previously validated in vivo by Ben-Simon et al. (2024). **c**, **d** Data are presented as mean values ± SD (standard deviation) across *n* = 10 cross-validation folds. Individual fold values are shown as black dots. **c** Model performance comparison (Test set Pearson correlation) of the models shown in (**b**). **d** Model performance comparison (test set Pearson correlation) of the S2F model trained on the full 10x-based cell count and cell depth compared to S2F models trained on different amounts of HyDrop v2-based data at full sequencing depth. Source data are provided as a Source Data file. **e** Heatmap of in vivo validated enhancers (Ben-Simon et al., 2024) identified true positives by the 10x-based and HyDrop v2-based models. Source data are provided as a Source Data file.

After preprocessing from raw fastq to integrated embedding together for 10x (v1 and v2) and HyDrop (v1 and v2)-based data, the combined data set of 92,363 cells (Fig. 2a) was annotated based on cell-type-specific regions from two published data sets^5,19^. Next, we subset for 10x (v1 and v2) and HyDrop v2 cells. For both subsets, we follow the default data preprocessing methodology within CREsted and normalize the pseudobulk peak heights over a set of consensus peaks to ensure similar peak heights per cell type for generally accessible regions. Per consensus region, the average peak height across cell types over the center 1000-bp window is set as the regression prediction target. We use the 2114-bp window centered on the target region as inputs for CNNs with 6.3 M parameters, each containing eight dilated convolutional blocks and an output block consisting of fully connected layers, inspired by the ChromBPNet model architecture^13^. We trained models with identical hyperparameters on HyDrop v2- and 10x-based data, and we varied the training data in terms of the number of cells and the number of reads in the preprocessed data (Fig. 2b). For each scenario, we performed baseline model training on all peaks, followed by a finetuning stage on cell-type-specific peaks. Additionally, we performed k-fold cross validations (k = 10) for each model and used the average predictions over the folds of the finetuned models in our analyses. In total, we trained 220 models starting from the same hyperparameters (11 scenarios x finetuning and baseline x 10 folds).

The finetuned models trained on all available cells (Table S1) and at the full available read depth (10x: 19,478 cells, Mdn = 65.04kRPC, SD = 14.9; HyDrop v2: 67,080 cells, Mdn = 45.48kRPC, SD = 12.7) perform comparably (Test Pearson correlation in Fig. 2c), with 10x-based data being sequenced slightly deeper while the HyDrop v2-based data contains more cells. Also, when down-sampling to 36kRPC, we observe a slightly superior performance of the HyDrop v2 model, possibly due to the higher cell number compared to the 10x training data (Table S2). For a direct comparison, we randomly sampled the HyDrop v2-based data to match the 10x-based cell count per cell type at full read depth (Table S1). Equally, we adapted the number of 10x-based cells based on the HyDrop v2-based cell count, where for the endothelial class, fewer cells were available at 36kRPC (HyDrop v2: 407 cells, 10x: 424). Overall, reducing the cell number leads to a drop in performance for HyDrop v2-based models at full and downsampled sequencing depth (Fig. 2c). Importantly, reducing the number of cells of the training data set mainly impacts the average performance across all predictions (macro AP), while reducing the sequencing depth per cell does not impact the precision and recall (Fig. S3a).

Based on these findings, we determined the number of additional HyDrop v2-based cells needed to generate a model comparable to one trained on 10x-based data. As the sequencing depth did not largely alter the model performance, we opted for the full depth data to be as close to the experimental reality as possible. To the reduced dataset of HyDrop v2, we added 25, 50, or 75% of the left-out cells and again trained ten-fold validated S2F models. The steepest improvement in model performance can be observed when adding 25% to the downsampled cell number, i.e., going from 19,478 to 31,374 cells (Fig. 2d).

In addition to model performance parameters, we evaluate how well the S2F models trained on different datasets predict enhancer sequences as active (Fig. 2b) that were previously experimentally proven to be indeed active in vivo in mouse motor cortex cell types^5,20^. The S2F models trained on the data at full read depth (Fig. 2e) or downsampled to 36kRPC (Fig. S3b) score comparably between 10x-based training data and HyDrop v2-based training data, both at the full and reduced number of cells. Thus, even though we see small differences in model performance (Fig. 2b, c), all S2F models trained on different scenarios of HyDrop v2-based data identify genomic enhancer sequences that were previously validated as active enhancers in vivo^5,20^.

In conclusion, reducing the read depth to 36kRPC does not impact the model performance. Reduced performance for S2F models trained on data with a slightly lower read count in peaks (HyDrop v2: FRIP Mdn = 0.51, SD = 0.09; 10x v2: FRIP Mdn = 0.54, SD = 0.1) can be compensated by adding at least 25% of the left-out cells to the training data. Model improvements upon adding training data highlight the need for large-scale datasets beyond high-quality data. Given the reduced library costs of the HyDrop v2 platform compared to 10x, adding 25% extra cells (increasing it from 19,478 to 31,374 cells) would still only increase the costs by 116 euros (from 191.34 to 308.21 euros) compared to 1,657.94 euros (from 2,714.65 to 4,372.59 euros) if the current commercial alternative (10x Genomics v2) were used.

### Comparison of S2F sequence explainability in the mouse cortex

**Fig. 3:**
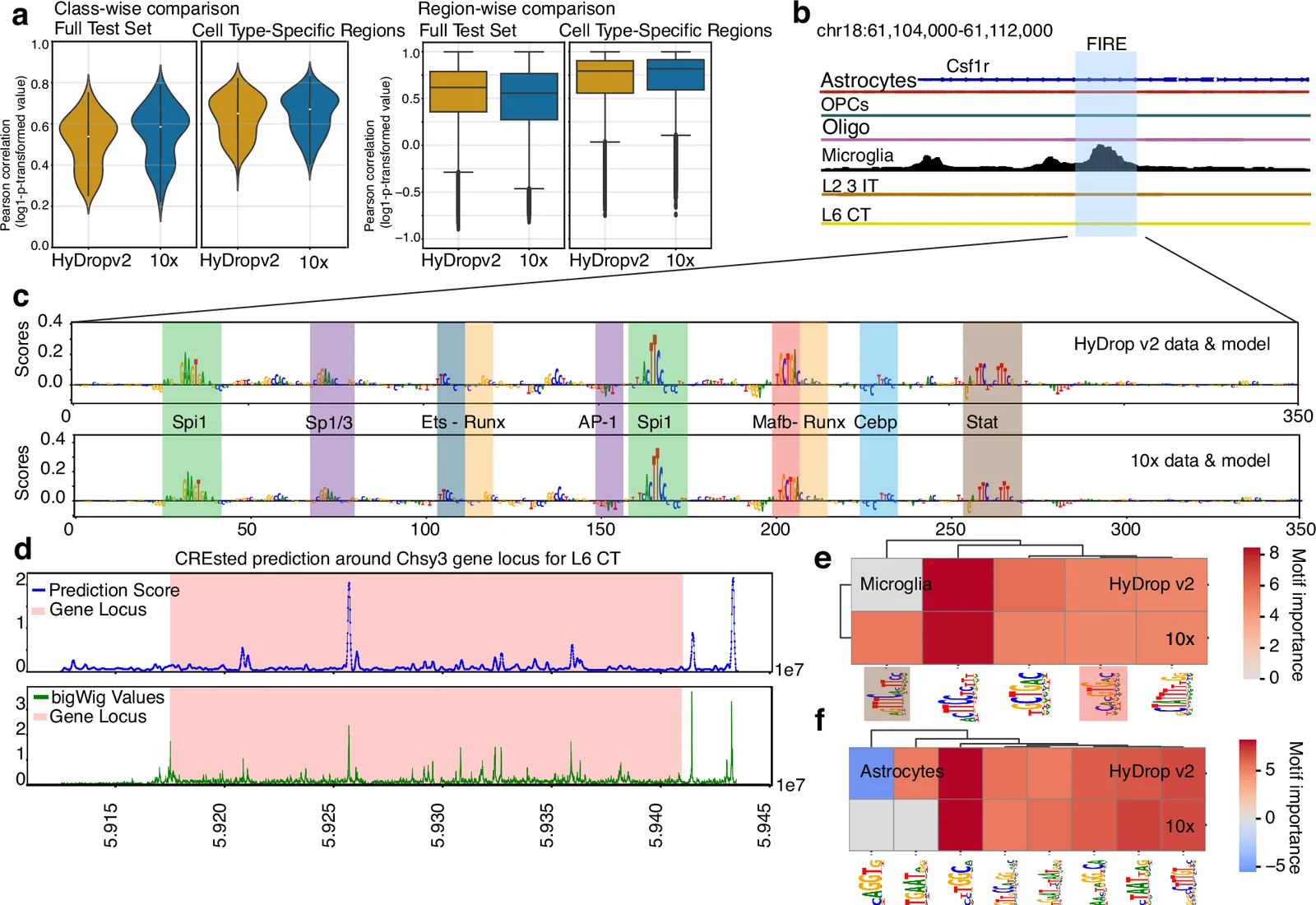
Comparative analysis of S2F explainability. **a** Model comparison of sequence models trained on HyDrop v2-based data (orange) and the 10x-based data (blue). The accuracy of the predictions evaluated based on the ground truth of region accessibility is compared on class-wise (left) and region-wise level (right) for both the full test set in the data and cell type-specific test sets. Class-wise correlations: *n* = 160 measurements (16 cell types × 10 cross-validation folds). Each data point represents correlation across all regions for one cell type in one fold. Region-wise correlations: *n* = 808,843 regions (full dataset) and *n* = 127,944–132,078 regions (cell type-specific) evaluated across 10-fold cross-validation. Each data point represents an independent Pearson correlation between predicted and observed chromatin accessibility for a single genomic regulatory region. **b** Genome tracks of cell-type-specific chromatin accessibility of the FIRE enhancer (mm10 chr18:61108475-61108975), regulating Csf1r expression in microglia. Track height is set to 0–15 for all samples. **c** Nucleotide contribution score of the FIRE enhancer from a sequence model trained on HyDrop v2-based data (top) and a model trained on 10x-based data (bottom). Previously described TF binding sites are highlighted for the corresponding TF. **d** Prediction by the HyDrop v2-based sequence model on HyDrop v2-based data of chromatin accessibility (top) for L6 CT around the Chsy3 gene locus compared to actual chromatin accessibility plotted from the corresponding bigwig file (bottom). Evaluation of motif importance scores of highly relevant regions for cell type identity indicated by the model trained on HyDrop v2-based and 10x-based data for microglia (**e**) and astrocytes (**f**), motifs shown in c are highlighted.

To investigate whether the S2F model trained on the full data set of HyDrop v2-based data at full read depth captures relevant biological information, we evaluate its peak height predictions and sequence explanations. Looking at class-wise and region-wise comparison of predicted and observed peak heights, the finetuned models of HyDrop v2 and 10x score comparably on both the full test set as well as the cell-type specific regions (Fig. 3a).

Similar to previously published models^11,14^, the HyDrop v2-based model recovers the correct microglia TFBS in the FIRE enhancer that replicates the findings of the 10x-based model (Fig. 3b). Furthermore, the HyDrop v2-based model can correctly predict chromatin accessibility in an unseen locus, such as the *Chsy3*, a marker gene of L6CT neurons (Fig. 3c), as seen in comparable models^14^.

To further investigate the sequence explainability of each model for cell-type-specific enhancers, we performed de novo motif discovery using *tfmodisco lite*^21^. Per model (combination of 10-fold models trained on 10x-based or HyDrop v2-based data), we identified enriched patterns, alongside their individual instances (called seqlets). For the highest and lowest scoring patterns, we find the same patterns with both models for astrocytes and microglia (Fig. 3e, f), neuronal cell types, and oligodendrocytes (Figs. S4a–d).

S2F models trained on commercial scATAC data have been shown to accurately identify cell-type-specific regions and predict TF binding sites^11^. Here, we show that an S2F model trained on non-commercial data generated with our open-source HyDrop v2 protocol achieves similar performance to an equivalent 10x model and, importantly, can recover cell-type-specific regions and TF-binding sites and correctly predict in vivo validated enhancers in the adult mouse motor cortex.

### Comparison of enhancer prediction and sequence explainability in the fly embryo

**Fig. 4:**
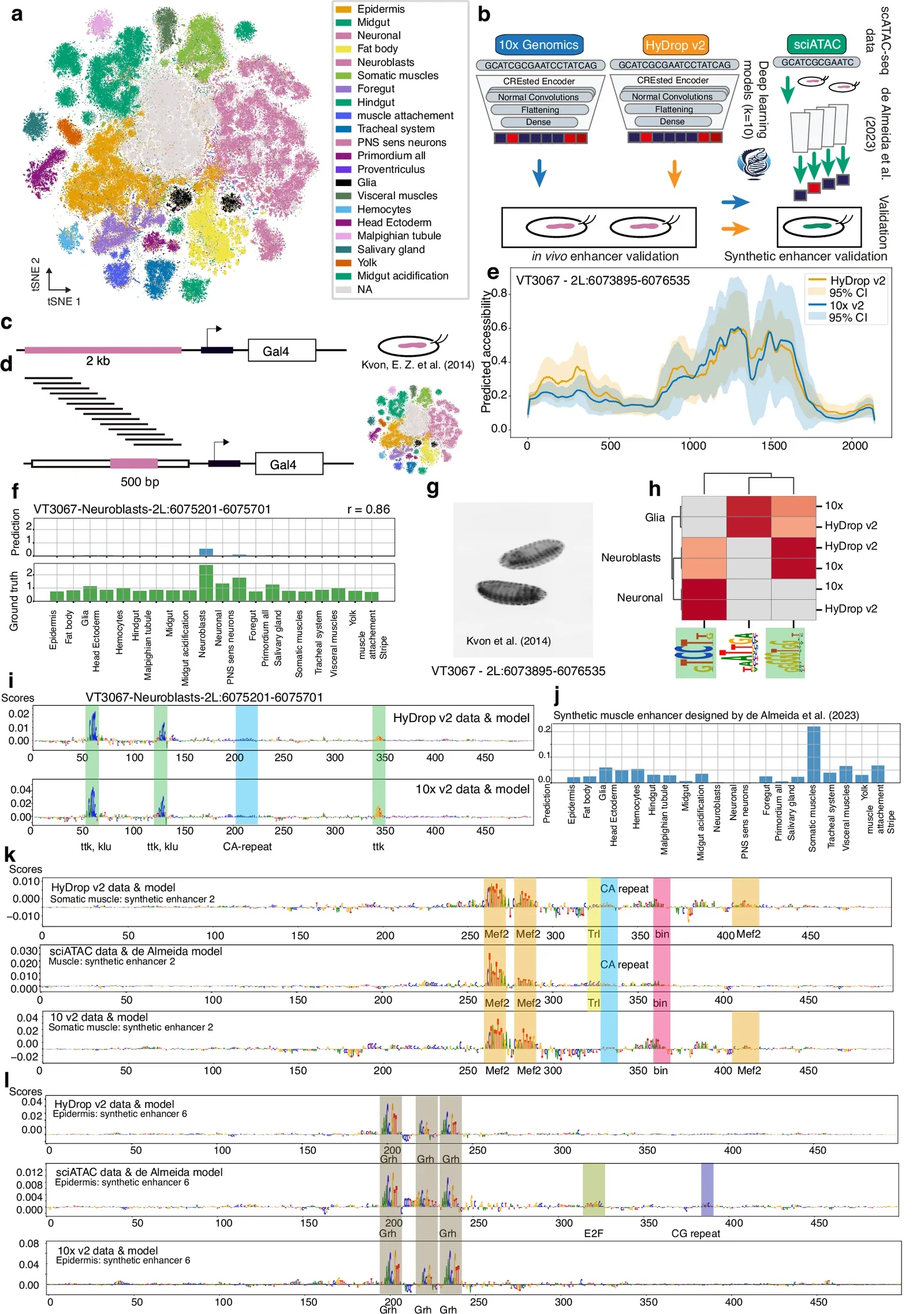
Analysis of *Drosophila* S2F models. **a ***t*-distributed stochastic neighbor embedding (tSNE) of 607,340 cells with HyDrop v2 (340,604, 18 experiments) and 10x v2 (266,736, 5 experiments), colored by cell type, batch correction according to wet lab protocol used to generate data. Data points were randomly shuffled before plotting. **b** Computational design evaluating scATAC techniques as training data for S2F models in k-fold cross-validation (*k* = 10). **c** VDRC library of transgenic flies with ~2 kb enhancers (pink) upstream of the minimal promoter and Gal4 reporter. 2693 enhancers stage 15–16 evaluated in vivo by Kvon et al. (2014). **d** Scanning of ~2 kb enhancers (**c**) with 500 bp sliding window (10 bp shift). **e** Predicted accessibility of 500 bp windows of VT3067 enhancer (VDRC library). The region coordinates are based on Kvon et al. (2014), accessibility as ground truth. Line plots show mean predicted accessibility across *n* = 10 cross-validation folds. Bands represent 95% confidence intervals (1.96 × SD). **f** Prediction versus ground truth of 500 bp region within 2 kb neuroblast enhancer. Source data are provided as a Source Data file. **g** In situ hybridization of VT3067-Gal4 reporter embryo stage 15 with antisense Gal4 probe by Kvon et al. (2014). **h** Motif importance scores by the 10x v2 data- and HyDrop v2 data-based models for neurons, glia, and neuroblasts, importance threshold of 5. **i** Nucleotide contribution score of the 500 bp within VT3067 enhancers of HyDrop v2-based and 10x v2-based models. **j** Prediction by the HyDrop v2 model of a synthetic enhancer accessibility designed for muscle cells in 10–12 h embryos by de Almeida et al. (2023). **k** Nucleotide contribution score of the synthetic enhancer shown in j across HyDrop v2, 10x v2, and sciATAC model. **l** Nucleotide contribution score of a synthetic epithelial cell enhancer designed by de Almeida et al. (2023). VDRC: Vienna Drosophila Resource Center, *ttk: tramtrack, Mef 2: Myocyte enhancer factor 2, bin: biniou, trl: Trithorax-like, grh: grainyhead, E2F1: E2F transcription factor 1*.

Our *Drosophila* dataset of the last four hours of embryo development contains in total 607,330 cells and represents thus far the largest developmental atlas of the fruit fly (Fig. 4a). The HyDrop v2-based dataset (340,604 cells) contains 27% more cells than the 10x-based dataset (266,726 cells) while both datasets are sequenced comparably deep (10x v2: Mdn = 13.21kRPC, SD = 2.09; HyDrop v2: Mdn = 15.88kRPC, SD = 6.81). Following the previous insights of the mouse motor cortex in-depth model comparison, we investigate if the additional cells (Table S3) make the HyDrop v2-based model comparable to the 10x-based models, while the slight difference in read depth is negligible.

We trained separate S2F sequence models on the *Drosophila* embryo data based on 10x v2 and HyDrop v2 and compared their performance to a published model^12^ based on sciATAC data (Fig. 4b). Our S2F models are trained with the CREsted package^14^, with an additional fly-specific filtering on the standardized peak heights (see Methods). Both models score identically on region- and class-wise prediction of accessibility (Fig. S5a, b).

To evaluate the ability of the S2F models to capture biologically relevant information, we made use of validated enhancers^22^ analogous to the evaluation of the mouse models. Kvon et al. (2014) characterized 3557 developmental enhancers in vivo according to tissue specificity of enhancer activity. This enhancer database contains the regional coordinates of 2693 enhancers assayed in stages 15–16, of which 80 are highly active with a specificity of up to five tissues^22^. As the cloned genomic regions have a length of approximately 2 kb (Fig. 4c), we scanned the regions with a sliding window of 500 bp (Fig. 4d) with a 10 bp shift to identify the actual core enhancer region for the respective tissue (Supplementary Data 1). Previously, the enhancer was estimated to use the full 2 kb region^22^. Both models, as well as their respective 10-fold cross-validation models (shown as confidence intervals), identify the same 500 bp regions as primarily accessible across the 2 kb enhancers (Figs. 4e, S5c).

The S2F model trained on HyDrop v2 data predicts the core 500bp-sequence (Fig. 4d, e) of enhancer VT3067 being accessible in neuroblasts (NBs, Fig. 4f). Based on earlier in vivo validation of the 2 kb sequence, the enhancer is mainly active in the ventral nerve cord (VNC, Fig. 4g), a tissue with high NBs content^23^. Similarly, the observed in vivo activity^22^ (Fig. S5d) corresponds to the predicted accessibility in the matching cell type (Fig. S5e).

In the first wave of neurogenesis at the end of embryo development, NBs type 1 and 2 are found in the VNC, giving rise to neurons and glia^23,24^. The nucleotide contribution scores of both models, either trained on HyDrop v2 or 10x v2 data, indeed predict motifs of NB-specific TFs such as *klumpfuß* (*klu*)^25^ and *tramtrack* (*ttk*)^23^. Interestingly, performing *tfmodisco lite* identifies the *ttk* motif (TCCT, ACCC) in NB and neurons but not glia (Fig. 4h). The observed TF-motifs, such as *ttk* or aspecific CA repeats, match the reported motifs by the earlier central nervous system (CNS) S2F model trained on a combination of sciATAC data and enhancer activity data^12^, as NBs occur in the CNS of the developing *Drosophila* embryo^23^. Comparing somatic and visceral muscle cells, we observe different motifs, with, e.g., *dorsal* only being predicted in somatic muscle cells (Fig. S5h). The ability to identify different motifs in closely related tissues underlines the power of HyDrop v2-based S2F to capture biological relevance beyond superficial quality metrics. Additionally, we show that our S2F model can narrow down the core enhancer regions within a 2 kb window with sliding window scanning.

### Multi-class model refines synthetic enhancers in Drosophila development

Previously published *Drosophila* embryo S2F models trained on sciATAC-based data, one model per tissue as a guiding oracle^12^, were used to design synthetic enhancers for five tissues (Fig. 4a, CNS, brain, muscle, epidermis, and gut). Per tissue, the activity of eight enhancers was validated in vivo^12^. To compare our HyDrop v2- and 10x v2-based models with these sciATAC-based models, we evaluated the synthetic sequences designed by de Almeida et al. (2023).

Due to the higher resolution in the annotation of our data and the multi-class design that includes all cell types in one model, we could further specify synthetic enhancer 1^12^: It is designed for muscle cells and appears indeed specific for somatic muscles across the *Drosophila* embryo (Fig. 4j). We observe several muscle development-specific motifs for, e.g., *Myocyte enhancer factor 2* (*Mef2*) and *biniou* (*bin*). The HyDrop v2-based model recovers all motifs indicated by the sciATAC model, while the 10x model is missing one *Trithorax-like* (*trl*, Fig. 4k). Similarly, only the sciATAC-based motif uncovers a motif of the, however, tissue-aspecific *E2F transcription factor 1* (*E2f1*) in the nucleotide contribution scores of synthetic epidermis enhancer 6 (Figs. 4l, S6a). The typical *grainyhead* (*grh*) motif; a TF essential to epithelial cell fate^26^, is found by every model.

The multi-class design of our HyDrop v2-based S2F model allows for scoring synthetic enhancer activity across tissues. Synthetic enhancer 8, originally designed for epithelial cells, is mainly predicted to be accessible in glial cells based on our model trained on HyDrop v2-based data (Fig. S6c). Evaluating the nucleotide contribution indicates *repo* motif instances, being indicative of glia^23^, in both models trained on either 10x-based or HyDrop v2-based data (Fig. S6d). The mix of motifs from different tissues in this synthetic enhancer sequence, identified by our models, however, might explain the lower predicted activity in the original sciATAC-based model^12^.

Notably, not all of the previously designed synthetic enhancers showed (matching) in vivo activity for de Almeida et al. (2024)^12^. Especially, the brain and gut enhancer design is mentioned to be cumbersome. The *Drosophila* gut poses the difficulty of being developed from the ectoderm (foregut and hindgut) and endoderm (midgut)^27^, making a targeted synthetic enhancer design possibly less straightforward compared to tissues with one developmental origin. By including a large variety of tissues in one single model, we find that the synthetic gut enhancers are not specifically active in the target tissue^12^, but are predicted to be active in other tissues outside the gut (Figs. S6b, e, f). This offers a possible explanation for the lack of specificity observed by de Almeida et al. (2023).

### Comparison of Tn5 bias and transcription factor footprints between platforms

**Fig. 5:**
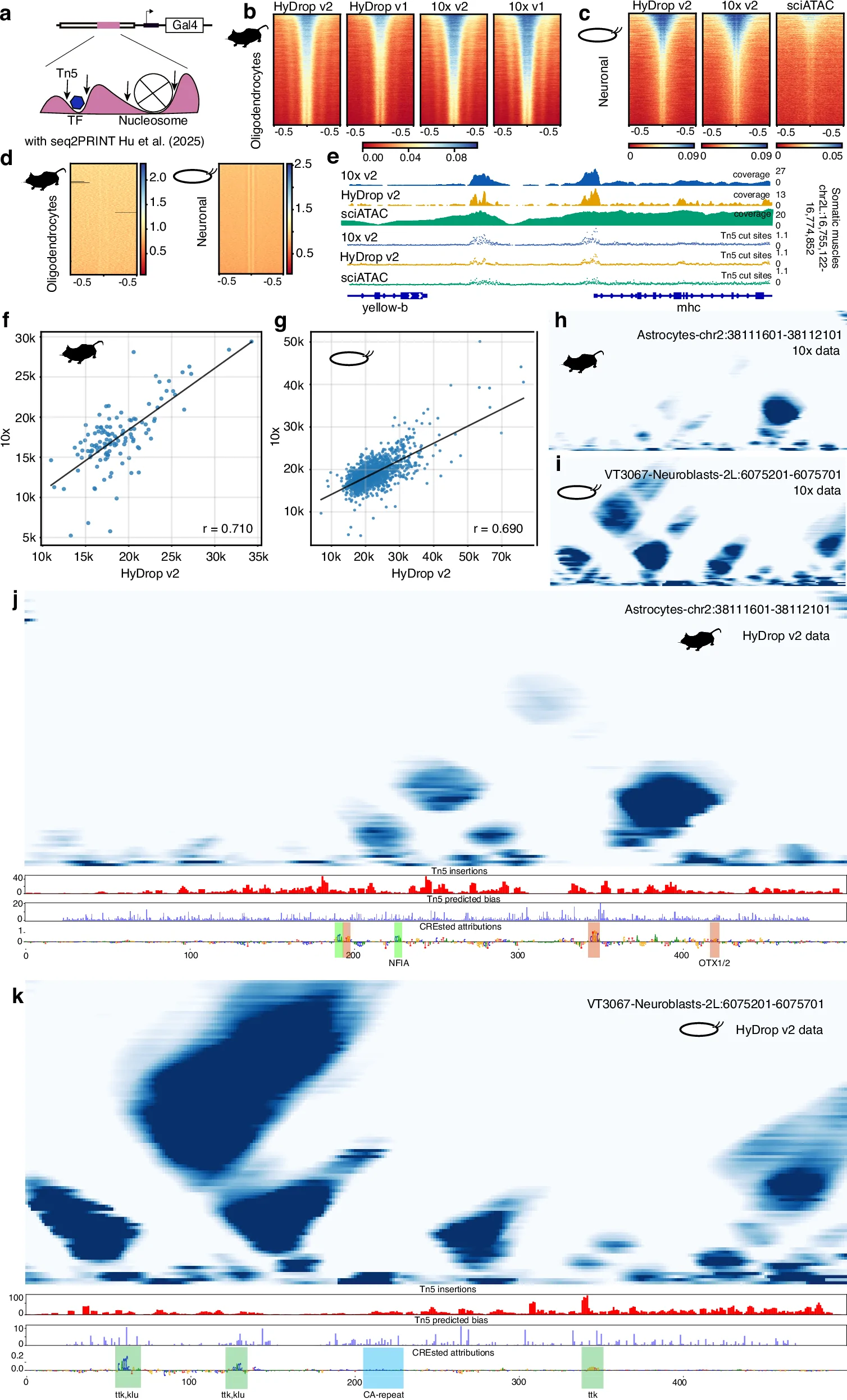
Footprint comparison between scATAC platforms. **a** Schematic overview of possible footprinting in the selected 500 bp region of VDRC library enhancers. The arrows represent Tn5 tagmentation locations. Footprinting is performed with seq2PRINT by Hu et al. (2025). Heat plot showing the regions of accessible chromatin ±0.5 kb around the center of DARs sorted by the highest (blue) to lowest (red) accessibility in for (**b**) HyDrop v2-, HyDrop v1-, 10x v2-, and 10x v1-based data in mouse and (**c**) HyDrop v2-, 10x v2-, and sciATAC-based data in fly. **d** Species-specific Tn5 bias by Hu et al. (2025) plotted on cell type-specific DARs. **e** Genome coverage tracks (upper) and cut site insertion of Tn5 (lower) of cell-type specific DAR down-sampled to the smallest count present (8916 somatic muscle cells). The shown genome tracks are normalized for the fragment count. Source data are provided as a Source Data file. Correlation scatterplot for the sum value of footprints in validated enhancer regions captured in HyDrop v2- and 10x-based data in mouse (**f**) and fly (**g**). Source data are provided as a Source Data file. Multiscale footprint of the region (*mm10* chr2:38111601-38112101) in mouse astrocytes of 10x v2-based data only (**h**) and the 500 bp region in VT3067 in fly neuroblasts in 10x v2-based data only (**i**), **j** Multiscale footprint of the region (*mm10* chr2:38111566-38112137) in mouse astrocytes of HyDrop v2-based data only. Bottom tracks show the measured Tn5 insertion, the predicted Tn5 bias, and the nucleotide contribution scores based on the HyDrop v2-based S2F model. **k** Multiscale footprint of 500 bp region in VT3067 in HyDrop v2-based data only. The bottom tracks show the measured Tn5 insertion, the predicted Tn5 bias, and the nucleotide contribution scores based on the HyDrop v2-based S2F model. DAR: Differentially accessible region, VDRC: Vienna Drosophila Resource Center, TF: Transcription Factor.

Next, we benchmarked the performance of HyDrop v2-based data against 10x-based data to predict TF footprints. To this end, we applied the recently developed seq2PRINT framework^15^. seq2PRINT uncovers footprints of DNA-protein interaction in scATAC and bulk ATAC data after correcting Tn5 bias with a convolutional neural network^15^. The use of a species-specific Tn5 bias model^15^ permits the evaluation of possible footprints of nucleosomes and smaller proteins, such as transcription factors, in *Drosophila* embryo and mouse motor cortex data (Fig. 5a).

We first investigated whether the Tn5 bias differs between HyDrop v2-based and 10x-based data by comparing the Tn5 cut sites across platforms, species, and cell types. Tn5 cut sites in cells obtained by the HyDrop v2 or 10x platform per cell type appear uniformly flanking the center of the peak in the accessible regions in mouse (Figs. 5b, S7a) and fly (Figs. 5c, S7b). The footprint around the center of the peak reflects the seq2PRINT-given Tn5 bias that is consistently observed in each cell type (Figs. 5d, S7c, d). Interestingly, the footprint (the double vertical line) tends to be more visible in *Drosophila*. Performing library preparation with the same Tn5 concentration as in mouse tissue (see Methods) results in a higher insertion ratio of Tn5, relative to the small fly genome of 180 Mb^28^, leading to shorter fragments in *Drosophila* (Fig. S7e) compared to mouse (Fig. S7f). Importantly, just as in the mouse samples, the Tn5 bias does not differ between HyDrop v2 and 10x in *Drosophila* samples (Figs. S7e, f). Comparing 10x- and HyDrop v2-based data with sciATAC-based data, the fragment size appears longer, which can also be observed in the genome coverage of single regions (Fig. 5e top, Fig. S7g top). Looking at the Tn5 cut sites in sciATAC-based data, however, the pattern resembles the 10x- and HyDrop v2-based data more closely in individual genomic regions (Fig. 5e bottom, Fig. S7g bottom) or genome-wide level (Fig. 5c, Fig. S7b). The difference in coverage results in a lack of information in sciATAC-based fly embryo data. This might explain the difficulty of integrating it with 10x- and HyDrop-based data and may play a role in the need for fine-tuning of earlier DL models in *Drosophila*^12^.

We continued by applying the same species-specific correction model^15^ in both datasets. In mouse and in fly, the TF footprints in 10x-based and HyDrop v2-based data correlate well for the genomic regions of the in vivo validated enhancers (Fig. 5f, g). We observed possible footprints coinciding with motif locations predicted by the S2F models that match between HyDrop v2 and 10x v2-based seq2PRINT predictions in mouse (Figs. 5h,j S7h) and fly (Figs. 5i, k, S7i).

## Discussion

To date, scATAC benchmarking papers have not evaluated the suitability of specific scATAC-seq technologies to train sequence-to-function (S2F) deep learning models^9,17^, while DL tool (benchmarking) papers did not compare training data generated on different platforms, read-depth, Tn5 bias, or number of cells^5,11,13–15^. Identifying cell-type-specific regulatory sequences from scATAC-seq data has become a driving force behind the unraveling of gene regulatory networks. Taking confounding factors into account when working with highly complex datasets is essential to eliminate DL model variability to understand enhancer grammar.

By benchmarking HyDrop v2-based data at the level of S2F models against commercial gold standard data, we emphasize the need for quality assessment beyond standard metrics to evaluate the value of a newly developed platform for research and beyond.

First, we generated atlases of the mouse motor cortex with 67k high-quality cells (HyDrop v2) and the developing embryo of age 16-20 h after egg laying of *Drosophila melanogaster* with 340k cells using the HyDrop v2 platform and 266k cells using 10x Genomics. The costs of generating the HyDrop v2 library of the mouse cortex across 35 experimental “runs” accumulate to merely 1/14 of the costs of 10x v2-based library preparation, excluding the sequencing cost. With the improved workflow, a single trained technician can perform 16 HyDrop v2 runs in 1.5 days. Thus, generating >100k cells of the mouse cortex library becomes feasible within a few days, making large-scale data generation with the HyDrop v2 platform possible at low cost and large scale.

After library preparation, the batches are sequenced to a desired read depth per cell. Here, HyDrop v2 libraries reach a comparable number of unique fragments per cell to 10x data but require a higher sequencing depth (i.e., having a lower sequencing efficiency). This might be due to a lower efficiency of ATAC fragment and barcode pairing in the custom setup. The barcoding efficiency arises from the Tn5 fragmentation and PCR polymerase extension. Even though we solved the bead impurities of HyDrop v1 to a large extent, some impurities and performance issues may remain. Other factors influencing the performance might be the nuclear lysis and the number of oligos released from the bead. Comparing the effect of sequencing depth on S2F model performance, however, shows that downsampling to 36kRPC (mouse) only slightly alters the model performance while still being able to capture biologically relevant features in the form of in vivo validated enhancers. Importantly, our libraries appeared largely saturated at this depth, offering a possibility for cost reduction by providing a potential cutoff for sequencing depth when producing data for DL purposes. This negligible effect of sequencing depth in DL tools is in line with earlier findings showing that the number of DARs does not depend on sequencing depth^9^.

Just as HyDrop v1^9^, HyDrop v2 data integrates seamlessly with data from other microfluidics-based platforms. The assay can therefore also be used for protocol optimization before investing in a major experiment, while still integrating with the final data. Eventually, data generated with different droplet-based platforms can be combined for large-scale atlases.

Moving on to the effect of cell number, i.e., amount of training data, we observe that HyDrop v2-based models require a higher number of cells to reach the performance of a 10x-based model. To determine the surplus of HyDrop v2-based cells needed, we evaluated the model performance of three additional HyDrop v2 models with different amounts of training data. Here, doubling the training data set size results in a comparable model performance to 10x-based data. While purely HyDrop v2-based models of the mouse motor cortex require a higher amount of training data, the cost of generating such a data set with 60% more cells is still eight times lower compared to 10x Genomics. Importantly, when evaluating the potential to identify in vivo validated enhancers, the fully downsampled HyDrop v2-based model still identifies a similar number of true positives as the 10x-based model, showing a high on-par performance on the biological level despite slightly lower model performance. The full HyDrop v2-based model (i.e., without reducing the training cell number) scores comparable to better on class- and region-wise comparison of predicted and observed peak height in both mouse and fly. This finding underscores the power of using custom platform-based training data for DL. More cells in the training data set increase the model’s ability to capture validated enhancers, which is in line with previous, but untested, statements that DL model performance strongly depends on the quality and size of training data^5^.

As the size of the atlas (i.e., training data) increases, the difference between HyDrop v2-and 10x-based models becomes smaller. The mouse atlas is relatively small (67,080 HyDrop v2 cells and 19,478 10x cells) and shows more pronounced differences between HyDrop v2 and 10x S2F models. The Drosophila atlas is much larger (340,604 HyDrop v2 cells and 266,736 10x cells), and consequently, the difference between HyDrop v2- and 10x-based S2F models is reduced. This again illustrates that S2F models become more robust as the size of the training data increases.

In the fly, we see an increase in neuronal/CNS cells in the HyDrop v2 dataset, as they are the largest cell group in the last stage embryo^29^, leading to an overrepresentation when more cells are sampled. A similar effect is observed in the mouse cortex data regarding oligodendrocytes. It has, however, previously been shown that S2F models can handle input well that is based on varying cell counts^5,14^.

With the HyDrop v2- and 10x-based models of the late-stage *Drosophila* embryo, we were able to pinpoint the core enhancer region within in vivo tested regions of ~2 kb size. The 10-fold cross-validation of the models shows that there is indeed variability between S2F models in cell type-specific accessibility prediction, but we, nevertheless, observe a large agreement between the cross-validations in enhancer region selection. With motif enrichment analysis and deep learning-based de novo motif discovery, we replicate earlier findings based on commercial data, underscoring the ability of HyDrop v2-based data to uncover biologically relevant information comparable to 10x, just as in mouse.

We also compared both our *Drosophila* S2F models to a previously published model that was trained on sciATAC-seq3 data, a plate-based assay^12,30^. The five models by de Almeida et al. (2023), one per target tissue, had been finetuned with additional in vivo enhancer assays, as opposed to our models, which are trained on scATAC-seq data only. In the mouse, it has been shown that the recent advances in S2F model design allow for improved models without the need for additional finetuning based on additional data modalities^5^. Besides differences in model design, de Almeida et al. (2023) have trained their models on data of 10-12 h of embryo development^12^, while our models are trained on a slightly later developmental stage of *Drosophila* development. The synthetic enhancer sequences designed for gut, epidermis, and muscle tissue^12^ are identified as target sequences matching the respective cell types in our multi-class model. The high level of annotation in our data allows for specifying the synthetic enhancer for the muscle to be active in somatic muscle, which is also confirmed by the enhancer’s in vivo validation score^12^. Here, de novo motif discovery driven by the S2F HyDrop v2-based model uncovers additional motifs important in somatic versus visceral muscles.

Finally, we investigated the genome-wide Tn5 bias in HyDrop v2-based and 10x-based data across cell types and species. To our knowledge, no benchmarking study has compared the Tn5 bias between different platforms. We find that microfluidic platforms (HyDrop v2 and 10x) using their respective standard Tn5 produce identical Tn5 biases, making the data usable for S2F models^13,14^ and footprinting with a standardized bias correction^15^. The larger fragment size in sciATAC-based data, however, might underlie the need for finetuning of the sciATAC-based S2F model by de Almeida et al. (2023) and the difficulty of integrating sciATAC data with HyDrop v2- and 10x v2-based data of the same developmental time stage. In contrast to sciATAC, HyDrop v2, and 10x v2-based data in *Drosophila* resemble the mouse genome coverage. Mouse genome coverage tracks of 10x data were previously used for successful model generation in mouse^5,11^, cell lines^10^, or the adult fly^6,10^. This finding underscores the need for a priori evaluation of the input data beyond cell number and sequencing depth for DL applications. Here, the use of cut sites might offer a possible solution, as already seen in some mouse models^14^. When evaluating predicted TF footprints in mouse and fly with seq2PRINT^15^, we identify similar multi-scale footprints in HyDrop v2- and 10x-based data, in mouse and fly.

Additional comparisons of other scATAC platforms for DL purposes might be needed to benchmark S2F modeling across a diverse range of experimental techniques. Another plate-based platform called SPATAC was used for zebrafish^31^ data generation. In the recent CREsted implementation, the authors show that SPATAC data yields enough information to train S2F models and design synthetic enhancers^14^. To our knowledge, no data for *Drosophila* or mouse cortex generated with SPATAC is available to compare with droplet- and other plate-based platforms.

By providing an evaluation of the effect of read depth, cell count, and Tn5 bias on S2F modeling, we exemplify the importance of close interactions between data generation technologies and machine learning. With the increased availability of  DL  tools, future benchmarking studies may be needed to reevaluate newly available bioinformatics tools on their performance on large-scale datasets, ideally composed of data generated on different platforms for cost-effective use of resources.

## Methods

### Ethical approval

The experiments on mice were conducted in line with KU Leuven’s ethical guidelines and approved by the Ethical Committee for Animal Experimentation at the Ethics Committee Research UZ/KU Leuven (EC Research) with the protocol number P007/2021. The use of the cell lines was approved by the Ethics Committee Research UZ/KU Leuven (EC Research) under project number S63316.

### Barcoded hydrogel bead manufacturing and storage

The microfluidic droplet generators for HyDrop v2 were produced following the HyDrop v1 protocol^17^ modified as described below. A detailed protocol can be found at https://www.protocols.io/view/hydrop-v2-bead-generation-amp-ligation-barcoding-8epv5n11dv1b/v2.

Dissolvable hydrogel beads for HyDrop v2 are synthesized similarly to previously published protocols^17,32^ and barcoded by three rounds of split-and-pool ligation reactions^33,34^. For synthesizing 2–3 mL batch of beads, 1.1 mL of Bead Monomer Mix (8.5–10% acrylamide (%T) (Sigma), 2–4% bisacryloylcystoylamine (%C) (Sigma), 10% Tris-buffered saline with EDTA and Triton X-100 (TBSET) (10 mM Tris-HCl pH 8; Thermo Fisher, 137 mM NaCl; Thermo Fisher, 2.7 mM KCl; Sigma, 10 mM EDTA; Thermo Fisher, 0.1% Triton X-100; Sigma), 4 μM acrydite primer, 0.6% ammonium persulfate; Sigma) was encapsulated into 60 μm diameter droplets in HFE-7500 Novac oil (Fluorochem, F051243) with EA-008 surfactant (RAN Biotech, 008-FluoroSurfactant-2wtH-50G) with 0.5% TEMED. Flow rates: monomer: 400 and oil: 750. The resulting emulsion was collected into 2 low bind 1.5 mL Eppendorf, layered with 200 μL mineral oil, and incubated at 65 °C for 14 hours, then paused at 12 °C. Excess mineral oil and the emulsion oil were removed, and three washes with 1 mL of droplet-breaking solution (20% PFO in HFE) were performed. Beads were pelleted at 1000 x *g*, 4 °C for 60 seconds and washed three times in 1 mL of 1% SPAN-80 in hexane (0.2 µm syringe filter). Beads were sequentially washed three times with TBSET and three times with TET buffer. Bead QC was performed as for HyDrop v1 previously (Zilionis et al., 2017; De Rop et al., 2022), and beads were filtered using a 70 μm filter EASYstrainer (Greiner, 542070) to exclude large bead contamination. The beads were stored in TET buffer at 4 °C.

For barcoding round one, on the day of barcoding, the beads were first washed with TET buffer by spinning down (3 minutes at 1000 x *g*, gentle braking), discarding supernatant, and resuspension in TET buffer. After centrifugation, the TET buffer was removed, and the beads were washed two times with pre-ligation buffer (10 mM Tris-HCl, pH 8.0, 30 mM NaCl, 1 mM MgCl_2_ 0.1% Tween-20) for 1 minute at 1000 x *g* with gentle braking. After washing, the supernatant was removed to obtain a compacted pellet of beads. A Hamilton microlab STAR robot was used for liquid handling in the deep well 96-well plates during barcoding. 22 µL of compacted beads was aliquoted in each well of the 96-well plate. Next, 16 µL of 2× ligation-primer buffer (100 mM Tris-HCl pH 7.5, 20 mM MgCl_2_) and 4 µL oligo mix cassette (200 µM) were added. The plate was spun, the contents mixed by vortexing, spun again, and placed on a PCR block (pre-heated to 75 °C with heated lid at 105 °C). To anneal the oligos, the heating was turned off after 3 minutes at 75 °C, which allowed gradual cooling to room temperature (about 2.5 hours). During this cooling process, the plate is taken off the PCR-block and vortexed every 30 minutes at 2000 RPM to resuspend the beads. When the temperature reached 33 °C, the plate was left at room temperature for 10 minutes. The Mantis (Formulatrix) was used to aliquot 10 µL ligase mix (50 mM Tris-HCl, pH 7.5, 10 mM MgCl_2_, 5.1 mM ATP, 100 U/µL T4 ligase (New England Biolabs, M0202)) to each well. Plates were sealed, vortexed in a vortex shaker, and centrifuged for 30 seconds at 1000 x *g*. Then the beads were mixed on a thermoblock shaker for 30 seconds at 2000 RPM. Ligation was performed for 60 minutes at 25 °C with shaking alternating between 1000 and 1600 RPM for 30 seconds each. Afterwards, the plate was incubated overnight at 16 °C on a thermoblock while being shaken at 1000 RPM. After the overnight incubation, the plate was kept at 4 °C for at least 30 minutes before we proceeded with the inactivation of the ligation by performing a cleanup step on the Hamilton with 40 mLSTOP-25 (10 mM Tris-HCl pH 8, 25 mM EDTA, 0,10% Tween-20, 100 mM KCl). The beads were transferred to a 50 mL falcon and incubated for 20 minutes. Afterward, the beads were spun down (3220 x *g* for 7 minutes, gentle braking), the supernatant was removed, and the beads were transferred to a 15 mL tube. Next, the beads were washed once with 15 mL of STOP-10 (10 mM Tris-HCl pH 8, 10 mM EDTA, 0.10% Tween-20, 100 mM KCl) and three times with 15 mL of TET buffer (centrifugation at 1000 x *g* for 1 minute). The second round of barcoding was started after two washes with pre-ligation buffer (centrifugation at 1000 x *g* for 1 minute) after which we transferred 22 µL compacted beads to each well of a 96-well plate.

For the round 2 barcoding, the 4 µL of the barcode 2 oligo cassette is added to 16 µL of 2× ligation-primer buffer in a 96-well plate, and annealing is achieved again with a PCR-block. Afterwards, the plate is spun to collect the condensate. The annealed cassettes were then transferred to the bead plate. Ligation was performed as discussed in the barcode step one.

Barcoding step 3 was done like step 1, with the exception that 23 µL of compacted beads was aliquoted in each well and mixed with 17 µL of 2× ligation-primer buffer and 3 µL of barcode 3 oligo mix (500 µM).

The barcoded beads are cleaned one last time on the Hamilton with STOP-25 buffer as described above and denatured with denaturation buffer (0.1 N NaOH, 1.68% Brij-35) for 10 min at room temperature at rotation (1 minute at 1000 x *g*). The beads are then washed with the neutralization buffer (100 mM Tris HCl pH 8, 10 mM EDTA, 0.1% Tween-20, 100 mM NaCl) for 10 min at room temperature with rotation (1 minute at 1000 x g). Finally, the beads are washed 3 times with TET buffer (1 minute at 1000 x *g*) and filtered twice using 70 um cell strainers.

The barcoded and cleaned beads are then incubated overnight at 4 °C in lysis buffer (125 mM Tris-HCl pH 7, 150 mM NaCl, 12.5 mM MgCl_2_, 1.25% Triton X-100, 0.4% BSA). The beads are washed twice more with lysis buffer (1 minute at 1000 x *g*) and aliquoted into capped PCR-strip tubes (40 µL) and stored at –80 °C.

### Sanger sequencing of barcoded beads

To test the purity of our barcoded HyDrop v2 beads, 1 µL of finalized barcoded beads was taken from their storage at −80 °C, and they were pelleted and washed three times with 0.04% BSA in PBS in a 1.5 mL microcentrifuge tube (centrifuge for 1 minute at 500 x *g*). The beads were diluted with PBS/BSA under a stereo microscope (Leica S8 APO) on a petri dish (Falcon 351008) until individual beads could be picked with a Stripper® pipette and 75 µm capillaries (MXL3-STR and MXL3-75, CooperSurgical). Individual beads were transferred consequently over three drops (5 to 10 µL) to ensure no other beads were present in the capillary. Next, single beads, in 0.5 µL of PBS/BSA, were transferred to PCR tubes containing 1.5 µL PBS/BSA. Afterwards, the capillary was washed three times in a large volume of PBS/BSA.

To the picked beads in the PCR tube, 18 µL PCR master mix was added (final concentrations: 1× KAPA HiFi hot start ready mix (Roche Cat. No. KK2602), 30 mM DTT, 1 µM ATAC-QC oligo) and the following PCR program was used: 72 °C – 3 mins, 98 °C – 30 s, 22 cycles of 98 °C – 10 s; 59 °C – 30 s; 72 °C – 30 s; and finally the amplification products were cooled to 4 °C. This product was purified using 1.5× Ampure XP beads, and the washed beads were resuspended in 10 µL of EB buffer (Qiagen). 8 µL of the eluate was used in the next PCR reaction (total volume is 20 µL with final concentrations: 1× KAPA HiFi hot start ready mix, 1 µM P7 indexing primer, 1 µM P5-Partial primer). Sanger sequencing was done by LGC Genomics using the P7 primer.

To test HyDrop v1 bead purity, the same protocol was followed, but different oligos were used (see https://www.protocols.io/view/sanger-sequencing-of-barcoded-beads-5jyl8xpzdv2w/v1).

### Husbandry and sample extraction

#### Mouse

Cortical brain tissue of six female C57B/6J mice aged P60 was used. Sex was not considered, as the study focuses on method development and data quality assessment rather than sex-specific analyses. The mice were housed under standard housing conditions in a pathogen-free facility with a 14 hr light, 10 hr dark light cycle from 7 to 21 hr at 40–70% humidity. Mice used in the study were 60 days old. Animals were euthanized using 0.05 mL/g BodyWeight. The chest cavity was opened, and the brains were flushed with 5 mL PBS through the left ventricle of the heart, followed by decapitation. Brains were dissected out, and cortices were collected. The motor cortex is dissected, snap-frozen in liquid nitrogen, and stored at -80 °C.

##### Nuclei extraction

Mouse nuclei for HyDrop-ATACv2 and 10x Genomics Single Cell ATAC v2 were extracted from mouse cortex samples that were snap-frozen in liquid nitrogen and stored at −80 °C. We transferred ~1 cm^3^ of frozen mouse cortex to 500 μL of ice-cold homogenization buffer (10 mM Tris-HCl, pH 7.5, 10 mM NaCl, 3 mM MgCl_2_, 320 mM sucrose, 0.1 mM EDTA, 0.5% BSA, 0.1% IGEPAL CA-630, 1× complete protease inhibitor, and 1 mM DTT) in a Dounce Homogenizer (KIMBLE, 1 mL). The tissue was left to thaw for 2 minutes before it was homogenized with 10 strokes of the loose pestle and 5 strokes of the tight pestle. Before the end of a 5-minute incubation, the homogenate was filtered through a 70 μm cell strainer (Corning), and the homogenizer and strainer were washed with homogenization buffer to obtain a final volume of 1 mL. The filtrate was transferred to a 1.5 mL DNA LoBind tube (Eppendorf) and centrifuged at 500 x *g* for 5 minutes. The supernatant was discarded, and the pellet was topped up to 520 μL with wash buffer 1 (10 mM Tris-HCl pH 7.5, 10 mM NaCl, 3 mM MgCl_2_, 320 mM sucrose, 0.1 mM EDTA, 0.5% BSA, 1× complete protease inhibitor, and 1 mM DTT) and resuspended. Next, we added 520 μL gradient medium (50% Optiprep, 10 mM Tris-HCl pH 7.5, 1 mM CaCl_2_, 5 mM MgCl_2_, 75 mM sucrose, 1× complete protease inhibitor, and 1 mM DTT) and mixed the entire volume by gentle pipetting. In a 2 mL DNA LoBind tube (Eppendorf) we layered 790 μL Optiprep cushion (29% Optiprep, 31 mM Tris-HCl pH 8, 77.5 mM KCl, 15.5 mM MgCl_2_, 129.2 mM sucrose) at the bottom and 1040 μL of the sample on top, gently without disrupting the cushion. The tube was then centrifuged at 9000 x *g* for 20 minutes at 4 °C. After the centrifugation, the debris on top was carefully removed with a 1 mL tip, and the rest of the supernatant was also carefully removed until 50 μL was left in the tube. The pellet was gently resuspended before we mixed it with 50 μL 2× permeabilization buffer (20 mM Tris-HCl pH 7.5, 20 mM NaCl, 6 mM MgCl_2_, 0.2% Tween-20, 0.02% IGEPAL CA-630, 0.02% Digitonin, 2% BSA, and 2 mM DTT) and left the nuclei suspension for 2 minutes on ice before we stopped the permeabilization by mixing it with 1 mL of wash buffer 2 (10 mM Tris-HCl pH 7.5, 10 mM NaCl, 3 mM MgCl_2_, 0.1% Tween-20, 1% BSA). The nuclei were pelleted by centrifugation at 500 x *g* for 5 minutes at 4 °C. The nuclei were resuspended in PBS with 0.04% BSA for HyDrop-ATAC v2 and in Diluted Nuclei Buffer for 10x Genomics Single Cell ATAC v2 processing.

#### Fly

The DGRP fly lines (DGRP-639, DGRP-502, DGRP-409) were purchased from Bloomington (25199, 28204, and 28278, respectively). No ethical regulations have to be considered when working with *Drosophila melanogaster*.

All flies were raised on a yeast-based medium and kept at 25 °C on a 12 h/12 h day/night light cycle. For embryo collection, flies were transferred to cages fixed to a plate with a normal yeast-based medium 24 hours before embryo collection. On the day of embryo collection, the plate was exchanged for a juice plate. The juice was prepped by adding 20 g agar and 22 g sucrose to 300 mL distilled water, boiling, and then mixing in 100 mL apple juice, 10 mL 95% ethanol, and 5 mL 100% glacial acetic acid. The flies laid eggs for four hours. Sixteen hours later, the embryos aged between 16 and 20 hours were collected from the juice plate with a small brush and transferred to a 70 µm nylon mesh filter. To dechorionate the embryos, the embryos were treated for 2 min with 5% bleach and then washed 4 times with 0.1% Triton X-100 in 1× PBS. After a final wash in 1× PBS, the embryos were transferred to a 1.5 mL tube, spun down for 2 min at 500 *g*, supernatant was removed, and the embryo pellet was snap frozen on dry ice and stored at −80 °C.

##### Nuclei extraction

Nuclei were isolated by using an adapted protocol based on the nuclei isolation protocol for single-cell ATAC sequencing (10x Chromium CG000169-RevE). Briefly, the embryos were resuspended in 500 µLcold lysis buffer (10 mM Tris-HCl pH 7.4, 10 mM NaCl, 3 mM MgCl_2_, 0.1% Tween-20, 0.1% IGEPAL CA-630, 0.01% Digitonin, 1% BSA), transferred to a Dounce homogenizer and incubated on ice for 5 minutes. The tissue was disrupted by 25 strokes with the loose pestle, incubated on ice for 10 minutes, and disrupted by 25 strokes with the tight pestle. To remove debris, the solution was filtered on a 10 µm nylon mesh filter and washed with 1 mLwash buffer (10 mM Tris-HCl, pH 7.4, 10 mM NaCl, 3 mM MgCl_2_, 0.1% Tween-20, 1% BSA). Nuclei were collected by centrifugation at 500 g for 5 mins at 4 °C, supernatants were carefully removed, and nuclei were resuspended in 50µL 1× Diluted Nuclei Buffer (10x Chromium). Nuclei quality and concentration were assessed by the LUNA-FL Dual Fluorescence Cell Counter.

#### Cell lines

For the barnyard test of HyDrop v2, cells from a human breast cancer cell line (MCF-7; RRID:CVCL_0031, female) and mouse melanoma cells were mixed. The cell lines were not authenticated nor tested for mycoplasma contamination.

For a detailed description of cell culture and cell dissociation, see De Rop (2022). In brief, MCF-7 cells (NCI-DTP Cat# MCF7, RRID:CVCL_0031) were cultured in RPMI1640 (ThermoFisher 11875093) medium supplemented with 10% FBS (ThermoFisher 10270–106), 1% penicillin/streptomycin (Life Technologies 15140122), and 10 ug/mL insulin (Sigma Aldrich I9278) and passaged twice per week. Mouse melanoma cells were cultured in DMEM (ThermoFisher 13345364) supplemented with 10% FBS and 1% penicillin/streptomycin and passaged once per week. All cell lines tested negative for mycoplasma prior to use. Cells were washed in PBS and dissociated into single-cell suspensions by adding 1.5 mL of 0.05% trypsin (Life Technologies 25300054) and waiting for 5 minutes. The single-cell suspension was centrifuged at 500 rcf for 5 min at 4 °C, and the resulting pellet was resuspended in PBS. This PBS wash was repeated once more, and the single-cell suspension was processed further.

##### Nuclei extraction

For the barnyard, the cell lines were mixed to equal parts and a pellet of one million dissociated cells or fewer was incubated on ice in 200 μL of ATAC lysis buffer (1% BSA, 10 mM Tris-HCl pH 7.5, 10 mM NaCl, 0.1% Tween-20, 0.1% NP-40, 3 mM MgCl_2_, 70 μM Pitstop in DMSO, 0.01% digitonin) for 5 minutes. 1 mL of ATAC nuclei wash buffer (1% BSA, 10 mM Tris-HCl pH 7.5, 0.1% Tween-20, 10 mM NaCl, 3 mM MgCl_2_) was added and the nuclei were pelleted at 500 x g, 4 °C for 5 minutes. The resulting pellet was resuspended in 100 μL of ice-cold PBS and filtered with a 40 μm strainer (Flowmi).

### HyDrop-ATAC v2 library preparation

A detailed description of the protocol can be found here https://www.protocols.io/view/hydrop-v2-atac-x54v97mmpg3e/v1.

#### Mouse

Between 7500 and 18000 nuclei, resuspended in PBS (supplemented with 0.04% BSA), were tagmented for one hour at 37 °C in 15 μL ATAC reaction mix (10% DMF, 10 mM Tris-HCl pH 7.4, 5 mM MgCl_2_, 5 ng/µL Tn5, 70 µM Pitstop in DMSO, 0.1% Tween-20, 0.01% Digitonin). Tagmented nuclei were placed on ice, and 95 µL of PCR mix (1.25× Phusion HF buffer, 15% Optiprep, 1.25 mM dNTPs, 75 mM DTT, 0.0625 U/µL Phusion HF polymerase, 0.0625 U/μL Deep Vent polymerase, 0.0125 U/μL ET SSB) was added. Next, 110 μL of nuclei mixed in PCR mix was co-encapsulated with 45 μL of HyDrop-ATACv2 beads in HFE-7500 Novac oil with EA-008 surfactant (RAN Biotech) using the Onyx microfluidics platform (Droplet Genomics). The resulting emulsion was split over two PCR tubes and was placed in a thermocycler with the following program: 72 °C – 15 mins, 98 °C – 3 min, 12 cycles of 98 °C – 10 s; 55 °C – 30 s; 72 °C – 1 min; and a final extension of 72 °C – 5 min, followed by a hold stage at 4 °C. To break the emulsion and remove excess oil, 125 μL of recovery agent (20% PFO in HFE) was added, and the PCR tubes were inverted 10 times to mix before the oily phase was removed. Next, we added 180 μL GITC mix (5 M GITC, 25 mM EDTA, 50 mM Tris-HCl pH 7.4), 10 μL Dynabeads, and 10 μL 2.4 M DTT to each of the aliquots and mixed everything by pipetting up and down 10 times and incubating for 10 minutes. Dynabeads were pelleted on a magnet and washed twice with 80% ethanol. Elution was done in 50 μL of EB-DTT-Tween (24 mM DTT, 0.1% Tween-20 in EB (10 mM Tris-HCl pH 8.5)). Next, a 1.2× Ampure bead purification was performed according to the manufacturer’s recommendations. Elution was done with 40 μL of EB-DTT (10 mM DTT in EB). The library was completed by amplifying the eluate in a total volume of 100 μL PCR mix (1× KAPA HiFi, 1 μM index i7 primer, 1 μM Universal P5 primer) with the following program: 95 °C – 3 mins, 12 cycles of 98 °C – 10 s; 63 °C – 30 s; 72 °C – 1 min; and a final extension of 72 °C – 1 min, followed by a hold stage at 4 °C. The final libraries were subjected to 0.4×–1.2× double-sided Ampure purification and eluted in 20 μL elution buffer (Qiagen).

#### Fly

Analogous to mouse nuclei, the HyDrop v2 protocol was performed on the lysed Drosophila nuclei, resuspended in 1x nuclei buffer. A total of 80,000 nuclei were resuspended in 40 μL of ATAC reaction mix (10% DMF, 10% Tris-HCl pH 7.5, 5 mM MgCl_2_, 4.7 ng/μL Tn5, 70 μM Pitstop in DMSO, 0.1% Tween-20, 0.01% digitonin) and incubated at 37 °C for 1 hr. Tagmented nuclei were placed on ice and counted with the LUNA-FL Dual Fluorescence Cell Counter. Per HyDrop reaction, a total of 37,500 tagmented nuclei were used in an 80 µl PCR reaction (1.37× Phusion HF buffer, 1.37% PEG-8000, 0.68 mM dNTPs, 89 mM DTT, 0.04 U/µL Phusion HF polymerase, 0.04 U/μL Deep Vent polymerase, 0.01 U/μL ET SSB). Next, 80 μL of nuclei mixed in PCR mix was co-encapsulated with 35 μL of HyDrop-ATACv2 beads in HFE-7500 Novac oil with EA-008 surfactant (RAN Biotech) using the Onyx microfluidics platform (Droplet Genomics). The resulting emulsion was placed in a thermocycler with the following program: 72 °C – 20 mins, 98 °C – 3 mins, 12 cycles of 98 °C – 10 s; 58 °C – 30 s; 72 °C – 1 min; and a final extension of 72 °C – 5 min, followed by a hold stage at 4 °C. To break the emulsion and remove excess oil, 125 μL of recovery agent (20% PFO in HFE) was added, and the PCR tube was inverted 10 times to mix before the oily phase was removed. Next, we added 180 μL GITC mix (5 M GITC, 25 mM EDTA, 50 mM Tris-HCl pH 7.4), 10 μL Dynabeads Silane One, and 10 μL 2.4 M DTT, and mixed everything by pipetting up and down 10 times and incubating for 10 minutes. Dynabeads were pelleted on a magnet and washed twice with 80% ethanol. Elution was done in 100 μL of EB-DTT-Tween (24 mM DTT, 0.1% Tween-20 in EB (10 mM Tris-HCl pH 8.5)). Next, a 1.1× Ampure bead purification was performed according to the manufacturer’s recommendations. Elution was done with 40 μL of EB-DTT (10 mM DTT in EB). The library was completed by amplifying the eluate in a total volume of 100 μL PCR mix (1× KAPA HiFi, 1 μM index i7 primer, 1 μM Universal P5 primer) with the following program: 95 °C – 3 mins, 10 cycles of 98 °C – 10 s; 63 °C – 30 s; 72 °C – 1 min; and a final extension of 72 °C – 1 min, followed by a hold stage at 4 °C. The final libraries were subjected to 0.4×–1.2× double-sided Ampure purification and eluted in 20 μL elution buffer (Qiagen).

scATAC-seq libraries were prepared according to the Chromium Single Cell ATAC reagent kits v2 user guide (10x Genomics, CG000496 Rev B). Briefly, the transposition reaction was prepared by mixing the desired number of nuclei with ATAC Buffer and ATAC Enzyme and was then incubated for 30 minutes at 37 °C. Tagmented nuclei were partitioned into nanoliter-scale gel bead-in-emulsions (GEMs). DNA linear amplification was then performed by incubating the GEMs under the following thermal cycling conditions: 72 °C – 5 min, 98 °C – 30 s, 12 cycles of 98 °C – 10 s; 59 °C – 30 s; 72 °C – 1 min, and finally a hold stage at 4 °C. GEMs were broken using Recovery Agent, and the resulting DNA was purified by sequential Dynabeads and SPRIselect reagent beads cleanups. Libraries were indexed by PCR using a Single Index kit N set A and incubated under the following thermal cycling conditions: 98 °C – 45 s, seven cycles of 98 °C – 20 s; 67 °C – 30 s; 72 °C – 20 s; and a final extension of 72 °C – 1 min, followed by a hold stage at 4 °C. Sequencing libraries were subjected to a final bead cleanup with SPRIselect reagent.

### Sequencing

HyDrop-ATAC v2 libraries were sequenced on an Illumina NextSeq2000 or NovaSeqX system using at least 43 cycles for read 1 (ATAC paired-end mate 1), 10 cycles for index 1 (sample index), 41 cycles for index 2 (HyDrop-ATAC v2 barcode), and 44 cycles for read 2 (ATAC paired-end mate 2).

### scATAC data processing

All in-house generated 10x and HyDrop samples were processed using the PUMATAC pipeline^9^ v0.0.1 (RRID:SCR_026624), built on Nextflow^35^ v21.04.3 (RRID:SCR_024135). With PUMATAC, we aligned the samples to the mm10 and dm6 reference genomes for mouse and fly, respectively, to write fragment files and perform quality assessment. For an in-depth description of the pipeline, see De Rop et al. (2023)^9^ and De Winter et al.^8^.

#### Barnyard

A mixed library of 5000 mouse melanoma and 5000 MCF-7 cells was generated following the standard HyDrop v2 library protocol as described above for the mouse cortex. The raw data were processed using the PUMATAC pipeline^9^ and aligned to a combined reference genome for mm10 and GRCh38. Cut-offs for TSS enrichment and the number of unique fragments per cell were set using the Otsu algorithm. Lastly, the fragments belonging to each genome were counted and compared.

#### Mouse samples

10x v2 and 10x v1 fastq files were downloaded from 10x Genomics database. The samples were processed using the PUMATAC pipeline^9^, together with the in-house generated data. Cut-offs were set per sample using the Otsu threshold for TSS enrichment and the number of unique fragments per cell. Next, fragments overlapping earlier published mouse candidate cis-regulatory regions^19^ were combined into a count matrix using pycisTopic^36^ (v2.0a0, RRID: SCR_026618). Potential doublets were removed using Scrublet (RRID:SCR_018098) with an automatic threshold set based on the expectation of 10% doublets. Cells were clustered based on initial pycisTopic^36^ Latent Dirichlet Allocation (LDA) using 80 topics. Based on this initial clustering, a new set of consensus regions was generated using MACS2, overlapping fragments recounted, and assembled into a second count matrix. Again, doublets were removed as previously, and LDA was performed with now 130 topics to capture all possible cell types. Artifacts attributed to batch effects were corrected using Harmony^37^, giving the sequencing technique as a variable instead of the sample identifier. Next, based on Leiden resolution 2.5, the cells were annotated using differentially accessible regions (DARs) of previously published datasets^20,38^. The newly annotated cell types were subdivided based on the sequencing technique of each sample. On the new variable, DARs were calculated based on imputed chromatin accessibility. Here, only regions passing one-versus-all Wilcoxon rank-sum tests with a 0.05 adjusted p-value are included. The top 1000 DARs per cell type per sequencing technique were investigated with motif enrichment analysis with pyCisTarget^7^ (v1.0a2, RRID:SCR_026626). Using pycisTopic^36^ to call peaks per cell type in pseudobulk with MACS2, peak BED files per cell type were generated. Additionally, the top 1000 DARs were compared between techniques. Here, the percentage of overlap is defined as the same regions being identified as DAR in a given cell type in the 10x v2 and HyDrop v2 data, divided by the total number of DARs of that specific cell type. Due to the processing of the consensus peaks in pycisTopic^36^, the regions are all 500 bp wide.

For BigWig files generation per cell type/sequencing technique, the lowest number of cells in one cell type in the respective sparsest sequencing technique was set as maximum. An equal number of cells for that cell type was randomly selected per remaining sequencing technique. Only cell types with more than 100 cells in each sequencing technique were included further on. Next, BigWig files for fragment coverage and Tn5 cut sites were generated for the downsampled, equal number of cells per technique with scATAC Fragment Tools^37^ (RRID:SCR_026643) normalizing the genome coverage (divided by the number of fragments. They were visualized with the integrative genome viewer (IGV, RRID:SCR_011793)^39^. The carrot plots (i.e., heat maps) were generated with deepTools2^40^ (v3.5.3; RRID:SCR_016366) plotting the accessibility per cell type and sequencing technique (BigWig) on the cell type-specific peak files (i.e., all regions accessible in that respective cell type). This was done for BigWigs based on fragment coverage and Tn5 cut sites.

For the comparison of quality metrics across microfluidic techniques, the samples were downsampled to 36,000 reads per cell (RPC) as indicated in the respective Figs. The plots were generated based on PUMATAC quality control evaluation with cell names defined based on previous annotations of the full sequencing depth count matrix.

#### Drosophila samples

Analogous to the mouse cortex data processing, the 10x v2 and HyDrop v2 data were processed with the PUMATAC pipeline^9^. Cut-offs were set per sample using the Otsu threshold with a maximum cut-off of 800 unique fragments and a TSS enrichment of 2. Fragments overlapping candidate regions of the earlier published Drosophila fly embryo dataset^30^ were combined into a count matrix using pycisTopic^36^ and clustered with 120 topics. Based on this initial clustering, a new set of consensus regions was generated with MACS2, overlapping fragments recounted and assembled into a new count matrix. LDA was performed in pycisTopic^36^ with 220 topics and clusters annotated with DARs from the published Drosophila embryo atlas^30^ on Leiden resolution 1.2. Correction of batch effects was performed with Harmony^37^ using the respective wet lab technique (sequencing technique and sample preparation parameter) as a variable. Analogous to the mouse data, the annotated cell types were subdivided among HyDrop v2 and 10x v2. On the new variable, DARs were calculated based on imputed chromatin accessibility. Here, only regions passing one-versus-all Wilcoxon rank-sum tests with 0.05 adjusted p-value were included. The top 1000 DARs per cell type per sequencing technique were investigated with motif enrichment analysis with pyCisTarget^8^. Additionally, the top 1000 DARs were compared between techniques. Here, the percentage of overlap is defined as the same regions being identified as DAR in a given cell type in and 10x v2 and HyDrop v2 data, divided by the total number of DARs of that specific cell type. Due to the processing of the consensus peaks in pycisTopic^36^, the regions are all 500 bp wide. Using pycisTopic^36^ to call peaks per cell type in pseudobulk with MACS2, peak BED files per cell type were generated.

For BigWig files generation per cell type/sequencing technique, the lowest number of cells in one cell type in the respective sparsest sequencing technique was set as the maximum. An equal number of cells for that cell type was randomly selected per remaining sequencing technique. Only cell types with more than 1000 cells in each sequencing technique were included further on. Next, BigWig files for fragment coverage and Tn5 cut sites were generated for the downsampled, equal number of cells per technique with scATAC Fragment Tools^37^, normalizing the genome coverage (divided by the number of fragments. They were visualized with the integrative genome viewer (IGV; RRID:SCR_011793)^39^. Just as in mouse, the carrot plots (i.e., heat maps) were generated with deepTools2^40^ for BigWigs based on fragment coverage and Tn5 cut sites.

For the comparison of quality metrics between HyDrop v2 and 10x v2, the samples were downsampled to 12,000 reads per cell (RPC) as indicated in the respective Figs. The plots were generated based on PUMATAC quality control evaluation, with cell names defined based on previous annotations of the full sequencing depth count matrix.

For the sciATAC data, the available fragment files of two experiments of embryos of 16 to 20 hours after egg laying from the publicly available data from the embryo atlas^30^ were downloaded. They were scored in two rounds, with their own consensus peaks in the second processing round. Here, the maximum cutoff was just as in 10x and HyDrop samples, set to TSS enrichment of two and 800 unique fragments. The data is clustered using pycisTopic^36^ with 220 topics and previous annotations^30^ assigned. Again, DARs were calculated and motif enrichment analysis was performed with pyCisTarget^8^ on the top 1000 DARs. Coverage and cut site BigWig files were calculated analogous to 10xv2 and HyDrop v2 fly data. No downsampling took place for sciATAC data.

### Model training

#### Mouse

Samples generated with the HyDrop v2 platform were extracted from the count matrix generated for the integrated samples. Again, using pycisTopic^36^, LDA was performed with 100 topics. No batch correction of the HyDrop v2 beads was needed. For downstream training of the HyDrop v2-based sequence model, pseudobulk profiles were generated in pycisTopic^36^ with MACS2 peak calling based on grouping cells in motor cortex cell types previously annotations of the full data set.

The sequence model was trained with the CREsted package^14^ (v.1.3.0RRID:SCR_026617) following the default peak regression parameters. Briefly, peaks were resized to 2114 bp length and normalized using the top 2% of the peaks. Next, the data was augmented with a sequence shift of 3 bp (to both sites) stochastically during training and the use of the reverse complement previous to model training using the dilated CNN model architecture.

Next to HyDrop v2-based data, we combined the 10x v1- and 10x v2-based data into a 10x dataset that serves as training data for the sequence-to-function models. The model was trained using the standard CREsted peak regression pipeline, by first pretraining on all consensus peaks and fine-tuning on a set of cell type-specific peaks. Just as in the HyDrop v2 model training, the same validation and training splits were handled, followed by resizing to 2114 bp length. The peaks were also normalized using the top 3% of the peaks, and the data was augmented. Again, the dilatedcnn model architecture was used for the model training.

We trained models with an identical model architecture and identical hyperparameters on HyDrop v2- and 10x-based data consisting of several experiments with varying numbers of cells, and number of reads in the preprocessed data. Additionally, we performed k-fold cross validations (k = 10) for each model. Each chromosome was split into k equal parts, and identical train and test regions per fold were used between Hydrop v2- and 10x-based data. For each scenario, we performed baseline model training on all peaks, followed by a fine-tuning stage on cell-type-specific peaks. Average performance metrics were then calculated over the test regions per the finetuned fold model. When validating external datasets, we used the average predictions over the folds of the finetuned models in our analyses. In total, we trained 220 models starting from the same hyperparameters (11 scenarios x 2 stages x 10 folds).

For downstream analysis, the average of the 10-fold cross-validation models was taken for the full HyDrop v2-based and 10x-based model. Recurrent sequence patterns were identified in the top 2,000 regions that overlap between 10x- and HyDrop v2-base data sets per cell type with *tfmodisco-lite* within the default parameters of the CREsted package.

Patterns between cell types and techniques were compared with the function *crested.tl.modisco.process_patterns* based on tomtom with the default parameters for similarity threshold of 3.5, trimming patterns based on an information content of 0.1, and discarding patterns with a threshold of 0.2.

##### In vivo validated enhancers recovery curves

A set of 122 functionally validated On-Target, mouse genomic enhancer regions in the mouse motor cortex was taken from a dataset provided by Ben-Simon et al.^20^. We evaluated the HyDrop v2 and 10x mouse motor cortex models by taking their predictions for these regions and calculating specificity through the *crested.pp._utils._calc_proportion* function. The specificity scores per model were then used to assess precision and recall, as was done in previous work^5^. Similarly, from the scATAC-seq peak heights, we calculated specificity scores to obtain precision and recall scores. Ground truth labels were obtained from the annotation of the validated enhancers.

As the 10x dataset had three cell types not found in the HyDrop dataset (SstChodl, Sncg, and VLMC), we merged them into the Sst, Vip, and Endo classes, respectively, by taking the max prediction and peak heights between the merged classes. The HyDrop v2 dataset, on the other hand, has an L4IT class not present in the 10x data, which is why it was merged with the L5IT class, also based on maximal prediction and peak height between the two classes.

#### Fly

Samples generated with the HyDrop v2 platform and 10x v2 were extracted from the count matrix generated for the integrated samples into two count matrixes. Again, using pycisTopic^36^, LDA was performed with 220 topics for HyDrop v2 and 200 topics for 10x v2. For downstream training of sequence models per sequencing technique, pseudobulk profiles were generated in pycisTopic^36^ with MACS2 peak calling based on grouping cells in previously annotated cell types from the full data set, excluding unannotated clusters.

The S2F model was trained with the CREsted package following a version of the default peak regression parameters adapted for fly. Briefly, the data was divided into training, validation, and test sets. The regions in chromosome 2 R were evenly divided into two to use as validation and test sets, and the remaining chromosomes were used as the training set. The original region length of 500 bp was kept. Peak heights were normalized per chromosome to a target mean accessibility of 0.5. After normalization, z-scores of peak heights were calculated per region. For each cell type, the default of the top 3000 regions with the highest z-scores was kept, and the accessibility values of all the other regions were set to zero for that cell type. CosineMSELoss (from crested.tl.losses) was used along with the default optimizer and metrics from the default CREsted peak regression configuration. The *deeptopic_cnn* model architecture was used with the following optional parameters: *filters* = *500, conv_do* = *0.5*. Since the default *deeptopic_cnn* model architecture has an output activation that’s incompatible with the regression task, it was replaced with a *softplus* activation. Two forms of augmentations were used: Before training, the training data was augmented using reverse complementation, which is achieved by adding the reverse complement of each sequence to the training dat,a keeping the same target vector as the original sequence. During the training, each input region was stochastically shifted up to 3 bp in either direction, still keeping the original target vector.

We identified the top 2,000 highest predicted regions per cell type per model and calculated the contribution scores of regions that are in the intersection of HyDrop v2 and 10x v2 data sets for the respective cell type. Later, these input sequences and contribution scores were used as input to *tfmodisco lite*-software (RRID:SCR_024811), using the available wrappers in CREsted package to find the most important patterns identified by 10x and HyDrop v2 models per cell type. An importance threshold of 5 was handled to select the most important motifs displayed

##### 10-fold cross-validation and selection of 500 bp regions in 2 kb window

To evaluate the ability of our S2F models to capture biologically relevant information, we made use of validated enhancers downloaded from https://enhancers.starklab.org/ and lifted over to dm6 genome annotation with UCSC. The original cell type annotations were manually translated into the cell types used in this paper (S3). Each ~2 kb enhancer region (merged for the annotated cell types) was scanned with a sliding window approach of 500 bp windows in steps of 10 bp over the whole enhancer with the 10-fold cross-validated models. Then, the 500 bp regions showing the highest CREsted predicted accessibility for all matching annotated cell types were selected based on the highest correlations between predicted and ground truth insertions.

##### sciATAC-seq-based models

The trained models were obtained from^10^. The enhancer activity models are used for all the analysis. The 501 bp synthetic DNA sequences were acquired from Almedia et al.^12^ The sequences were flanked on each side by 250 bp random sequences to acquire a 1001 bp sequence. The nucleotide contribution scores were calculated for one replicate model for each of the ten cross-validation folds, from the relevant tissue (except for the epidermis, where none of the provided replicates of fold 5 were working). The final contribution score was calculated by averaging all the contribution scores per sequence. The visualization of the contribution scores was done in the CREsted software.

### Footprinting with PRINT

Footprint scores calculations were carried out using the default PRINT v3 procedure as seen in https://ruochiz.com/scprinter_doc/index.html (scPRINTER python implementation of PRINT^8^). For both mouse and fly, we used the estimated Tn5 bias files provided by the original authors. As Tn5 insertion inputs, we used the Hydrop v2 and 10x v2 fragment files preprocessed by the PUMATAC pipeline^9^ for in-house generated data and cellranger-arc (10x Genomics) for public mouse multiome cortex data^41^.

For the mouse, we inspected the footprints of validated enhancer regions from the BICCN challenge^5^. For fly, we calculated footprints for all Drosophila embryo enhancers^22^ active in stages 15–16 downloaded from https://enhancers.starklab.org/ and lifted over to the dm6 genome annotation with UCSC. Each ~2 kb enhancer region was scanned with a sliding window approach of 500 bp windows in steps of 10 bp over the whole enhancer. Then, the 500 bp regions showing the highest CREsted predicted accessibility for all matching annotated cell types were selected for further analysis based on the highest correlations between predicted and ground truth insertions.

### Reporting summary

Further information on research design is available in the Nature Portfolio Reporting Summary linked to this article.

## Supplementary information

Description of Additional Supplementary Files

Supplementary Dataset 1

Supplementary Dataset 2

Reporting Summary

Transparent Peer Review file

Supplementary material

## Source data

Source Data

Source Data

Source Data

Source Data

Source Data

Source Data

Source Data

## Data Availability

The sequencing data and count matrix generated in this study have been deposited in the Gene Expression Omnibus database under the accession code GSE293575. For mouse cortex data, this includes the public data from 10x Genomics (https://www.10xgenomics.com/datasets/fresh-cortex-from-adult-mouse-brain-p-50-1-standard-1-1-0, https://www.10xgenomics.com/datasets/8k-adult-mouse-cortex-cells-atac-v1-1-chromium-x-1-1-standard, https://www.10xgenomics.com/datasets/8k-adult-mouse-cortex-cells-atac-v2-chromium-controller-2-standard) and earlier published data of De Rop et al. (2022) with raw data available at GSE175684 (https://www.ncbi.nlm.nih.gov/geo/query/acc.cgi). The scATAC coverage bigwigs can be downloaded at https://ucsctracks.aertslab.org/papers/hydrop_v2_paper/ or https://zenodo.org/communities/aertslab_hydrop_v2_paper/. The sciATAC-seq data of the Drosophila embryo age 16-20 h after egg laying were downloaded from Calderon et al. (2022, https://www.ncbi.nlm.nih.gov/geo/query/acc.cgi?acc=GSE190149). All processed fragment files are available at https://resources.aertslab.org/papers/hydrop_v2/. Source data are provided with this paper.

## Code availability

The code used to develop the model, perform the analyses, and generate results in this study is publicly available and has been deposited in GitHub at https://github.com/aertslab/HyDrop_v2_paper, under CC-BY license. The specific version of the code associated with this publication is mentioned in the Key Resource Table (Supplementary Data 2) and archived in Zenodo, accessible via https://zenodo.org/records/17434493^42^. A detailed explanation of the PUMATAC pipeline to process HyDrop v2 and 10x data can be found at https://github.com/aertslab/PUMATAC. Detailed instructions on CREsted can be found at https://github.com/aertslab/CREsted and https://crested.readthedocs.io/en/latest/changelog.html. For Seq2PRINT (scPRINTER in python implementation), we refer to the tutorial found here https://github.com/buenrostrolab/scPrinter by Hu et al. (2025).
