# Supplementary material for: Evaluating single-cell ATAC-seq atlasing technologies using sequence-to-function modeling
Source: Nat Commun. 2026 Jan 22;17:1951. doi: 10.1038/s41467-026-68742-4 (PMC12929571; doi:10.1038/s41467-026-68742-4)
Supplement: Supplementary file 1 [file 41467_2026_68742_MOESM1_ESM.pdf]

Description of Additional Supplementary Files:

Supplementary Data 1: VTID enhancers stage 15 and 16 by Kvon et al. (2014), annotated according to HyDrop v2 Drosophila annotation

Supplementary Data 2: Key resource table
