# Supplementary material for: Evaluating single-cell ATAC-seq atlasing technologies using sequence-to-function modeling
Source: Nat Commun. 2026 Jan 22;17:1951. doi: 10.1038/s41467-026-68742-4 (PMC12929571; doi:10.1038/s41467-026-68742-4)
Supplement: Supplementary file 2 [file 41467_2026_68742_MOESM4_ESM.pdf]

nature portfolio

Corresponding author(s): stein.aerts@kuleuven.be
NCOMMS-25-30735A

Last updated by author(s): Oct 22, 2025

# Reporting Summary

Nature Portfolio wishes to improve the reproducibility of the work that we publish. This form provides structure for consistency and transparency in reporting. For further information on Nature Portfolio policies, see our Editorial Policies and the Editorial Policy Checklist.

## Statistics

For all statistical analyses, confirm that the following items are present in the figure legend, table legend, main text, or Methods section.

| n/a | Confirmed | |
|---|---|---|
| ☐ | ☒ | The exact sample size (*n*) for each experimental group/condition, given as a discrete number and unit of measurement |
| ☐ | ☒ | A statement on whether measurements were taken from distinct samples or whether the same sample was measured repeatedly |
| ☐ | ☒ | The statistical test(s) used AND whether they are one- or two-sided<br>*Only common tests should be described solely by name; describe more complex techniques in the Methods section.* |
| ☐ | ☒ | A description of all covariates tested |
| ☐ | ☒ | A description of any assumptions or corrections, such as tests of normality and adjustment for multiple comparisons |
| ☐ | ☒ | A full description of the statistical parameters including central tendency (e.g. means) or other basic estimates (e.g. regression coefficient) AND variation (e.g. standard deviation) or associated estimates of uncertainty (e.g. confidence intervals) |
| ☐ | ☒ | For null hypothesis testing, the test statistic (e.g. *F*, *t*, *r*) with confidence intervals, effect sizes, degrees of freedom and *P* value noted<br>*Give P values as exact values whenever suitable.* |
| ☒ | ☐ | For Bayesian analysis, information on the choice of priors and Markov chain Monte Carlo settings |
| ☒ | ☐ | For hierarchical and complex designs, identification of the appropriate level for tests and full reporting of outcomes |
| ☐ | ☒ | Estimates of effect sizes (e.g. Cohen's *d*, Pearson's *r*), indicating how they were calculated |

*Our web collection on statistics for biologists contains articles on many of the points above.*

## Software and code

Policy information about availability of computer code

| | |
|---|---|
| Data collection | NA |
| Data analysis | A detailed explanation of the PUMATAC pipeline v0.0.1 to process HyDrop v2 and 10x data can be found at https://github.com/aertslab/PUMATAC. All data analysis and model training scripts are available at https://github.com/aertslab/HyDrop_v2_paper. The S2F models were trained using CREsted v1.3.0. Detailed instructions on CREsted can be found at https://github.com/aertslab/CREsted and https://crested.readthedocs.io. For Seq2PRINT (scPRINTER in python implementation, v3 used), we refer to the tutorial found here https://github.com/buenrostrolab/scPrinter by Hu et al. (2025). Additional software that are mentioned throughout the method section include: tfmodisco-lite v2.2.1 as part of CREsted, pycistopic v2.0a0, Integrative Genome viewer (2013), pyCisTarget v1.0a2, Nextflow v21.04.3 as part of PUMATAC v0.0.1, DeepTools2 v3.5.3 as part of CREsted, Keras v3.0 in the CREsted implementation, TensorFlow v2.19.0 as part of the CREsted implementation and PyTorch v2.6.0 as part of the CREsted implementation. All software/tools/algorithms/packages used in the study can also be found back in the Key Resource table S5 including the version number and identifier and are archived in Zenodo, accessible via https://zenodo.org/records/17434493. |

For manuscripts utilizing custom algorithms or software that are central to the research but not yet described in published literature, software must be made available to editors and reviewers. We strongly encourage code deposition in a community repository (e.g. GitHub). See the Nature Portfolio guidelines for submitting code & software for further information.

## Data

Policy information about availability of data

All manuscripts must include a data availability statement. This statement should provide the following information, where applicable:

- Accession codes, unique identifiers, or web links for publicly available datasets
- A description of any restrictions on data availability
- For clinical datasets or third party data, please ensure that the statement adheres to our policy

The sequencing data and count matrix generated in this study have been deposited in the Gene Expression Omnibus database under the accession code GSE293575. For mouse cortex data, this includes the public data from 10x Genomics (https://www.10xgenomics.com/datasets/fresh-cortex-from-adult-mouse-brain-p-50-1-standard-1-1-0, https://www.10xgenomics.com/datasets/8k-adult-mouse-cortex-cells-atac-v1-1-chromium-x-1-1-standard, https://www.10xgenomics.com/datasets/8k-adult-mouse-cortex-cells-atac-v2-chromium-controller-2-standard) and earlier published data of De Rop et al. (2022) with raw data available at GSE175684. The scATAC coverage bigwigs can be downloaded at https://ucsctracks.aertslab.org/papers/hydrop_v2_paper/ or https://zenodo.org/communities/aertslab_hydrop_v2_paper/. The sciATAC-seq data of the Drosophila embryo age 16-20h after egg laying were downloaded from Calderon et al. (2022). All processed fragment files are available at https://resources.aertslab.org/papers/hydrop_v2/.

## Research involving human participants, their data, or biological material

Policy information about studies with human participants or human data. See also policy information about sex, gender (identity/presentation), and sexual orientation and race, ethnicity and racism.

| | |
|---|---|
| Reporting on sex and gender | NA |
| Reporting on race, ethnicity, or other socially relevant groupings | NA |
| Population characteristics | NA |
| Recruitment | NA |
| Ethics oversight | NA |

Note that full information on the approval of the study protocol must also be provided in the manuscript.

# Field-specific reporting

Please select the one below that is the best fit for your research. If you are not sure, read the appropriate sections before making your selection.

☒ Life sciences ☐ Behavioural & social sciences ☐ Ecological, evolutionary & environmental sciences

For a reference copy of the document with all sections, see nature.com/documents/nr-reporting-summary-flat.pdf

# Life sciences study design

All studies must disclose on these points even when the disclosure is negative.

| | |
|---|---|
| Sample size | No formal sample size determination was performed. For Drosophila, we aimed to generate a number of cells comparable to those obtained with the 10x Genomics platform under standardized conditions. For mouse, the target was to capture >100 cells per cell type, which was largely achieved. Exact numbers are provided in Supplementary Tables 3–5. |
| Data exclusions | We only included cell types of the mouse motor cortex M1 as described in the the Results and Methods. As the data generated with the HyDrop v1 but also the public datasets from 10x Genomics are based on the dissected motor cortex M1, we excluded additional cells in HyDrop v2 data sets coming from additional brain regions. Here, the dissection was conducted broader leading to other cell types in the data set that could not have been properly compared to a matching cell type in the other data sets. This criterium was pre-established to the cell types present in M1. |
| Replication | Replication of data generation using 7 bead batches of HyDrop v2 beads. The standardized protocol is available at protocols.io, mentioned in the Methods section. Here, all replicates were successful leading to data generation. There are fluctuations observed in sequencing efficiency between the bead batches as shown in Figure S1f |
| Randomization | No formal randomization was performed. For mouse, samples were derived from six animals used throughout the study. For Drosophila, approximately 100 embryos spanning 16–20 h after egg laying were randomly collected and pooled and pooled per experiment. |
| Blinding | Blinding was not feasible or relevant, as the study involved automated computational analyses of existing datasets without subjective assessment or experimental intervention. |

# Reporting for specific materials, systems and methods

We require information from authors about some types of materials, experimental systems and methods used in many studies. Here, indicate whether each material, system or method listed is relevant to your study. If you are not sure if a list item applies to your research, read the appropriate section before selecting a response.

## Materials & experimental systems

| n/a | Involved in the study |
|---|---|
| ☒ | ☐ Antibodies |
| ☐ | ☒ Eukaryotic cell lines |
| ☒ | ☐ Palaeontology and archaeology |
| ☐ | ☒ Animals and other organisms |
| ☒ | ☐ Clinical data |
| ☒ | ☐ Dual use research of concern |
| ☒ | ☐ Plants |

## Methods

| n/a | Involved in the study |
|---|---|
| ☒ | ☐ ChIP-seq |
| ☒ | ☐ Flow cytometry |
| ☒ | ☐ MRI-based neuroimaging |

# Eukaryotic cell lines

Policy information about cell lines and Sex and Gender in Research

| | |
|---|---|
| Cell line source(s) | For the barnyard test of HyDrop v2, cells from a human breast cancer cell line (MCF-7; RRID: CVCL_0031) and mouse melanoma cells were mixed. |
| Authentication | Melanoma cell lines were derived by physical resection of primary cutaneous lesions as in Dankort et al., 2009 but not further authenticated. |
| Mycoplasma contamination | The cell lines were not tested for mycoplasma contamination |
| Commonly misidentified lines (See ICLAC register) | The cell lines were not authenticated. |

# Animals and other research organisms

Policy information about studies involving animals; ARRIVE guidelines recommended for reporting animal research, and Sex and Gender in Research

| | |
|---|---|
| Laboratory animals | Cortical brain tissue of six female C57B/6J mice aged P60 was used. The mice were housed under standard housing conditions in a pathogen-free facility with a 14 hr light, 10 hr dark light cycle from 7 to 21 hr at 40-70% humidity. Mice used in the study were 60 days old. |
| Wild animals | NA |
| Reporting on sex | NASex was not considered, as the study focuses on method development and data quality assessment rather than sex-specific analyses. |
| Field-collected samples | NA |
| Ethics oversight | The experiments on mice were conducted in line with KU Leuven's ethical guidelines and approved by the Ethical Committee for Animal Experimentation at the Ethics Committee Research UZ / KU Leuven (EC Research) with the protocol number P007/2021. |

Note that full information on the approval of the study protocol must also be provided in the manuscript.

# Plants

| | |
|---|---|
| Seed stocks | NA |
| Novel plant genotypes | NA |
| Authentication | NA |
