# Supplementary material for: Evaluating single-cell ATAC-seq atlasing technologies using sequence-to-function modeling
Source: Nat Commun. 2026 Jan 22;17:1951. doi: 10.1038/s41467-026-68742-4 (PMC12929571; doi:10.1038/s41467-026-68742-4)
Supplement: Supplementary file 3 [file 41467_2026_68742_MOESM5_ESM.pdf]

nature portfolio

Peer Review File

Evaluating single-cell ATAC-seq atlasing technologies using sequence-to-function modeling

Corresponding Author: Professor Stein Aerts

Parts of this Peer Review File have been redacted as indicated to remove third-party material.

This file contains all reviewer reports in order by version, followed by all author rebuttals in order by version.

Version 0:

Reviewer comments:

Reviewer #1

(Remarks to the Author)
This study introduces HyDrop v2, an improved, open-source microfluidic droplet platform for scATAC-seq. Compared to commercial systems, HyDrop v2 enhances sensitivity, reproducibility, and cost-efficiency, enabling the generation of large-scale single-cell chromatin accessibility atlases required for training S2F deep learning models. The method represents a valuable, accessible alternative to commercial platforms, particularly for labs with limited resources. However, several critical issues need to be addressed before further consideration. For example, the current version lacks a clear scientific narrative, does not control for key confounders in the AI evaluation, and misses the level of rigor expected for claims about model performance. The work could be valuable if reframed as a carefully controlled benchmark study of platform-generated data for AI tasks. To reach that level, it needs tighter experimental design, cleaner definitions, and more disciplined analysis.

Major Concerns
1. On line 192. It is unclear whether the authors ensured a fair comparison between methods regarding cellular diversity. Given that more cells were processed with HyDrop v2, it is unsurprising that more diversity was captured. The authors should explicitly state whether subsampling or normalization was performed to ensure comparability. A controlled analysis showing that HyDrop v2 captures equivalent diversity to 10x v2 given the same number of cells would be more convincing.

2. Lines 215 & 307, Fig. 2b & 4d. The current figures only show examples at single loci, which are not representative of genome-wide performance. The authors may consider includING genome-wide comparisons of DARs and fragment distributions, and reporting the total number of DARs and TFBS identified by each method. Extend the comparison beyond isolated regions and include summary statistics across the genome.

3. Lines 240–241. While the authors claim that HyDrop v2 models perform similarly to those based on 10x data, the visual evidence (Figure 3b) suggests that the 10x model performs better in certain classes. The authors should acknowledge this difference and provide quantitative metrics to support or qualify their claims.

4. Line 308, Fig. S5d. The HyDrop v2 peak within the hemocyte data differs notably from 10x v2, with an apparent lack of read coverage in specific regions. This discrepancy warrants further investigation—are these technical artifacts, biological variability, or data quality issues? Additionally, the sciATAC profile appears irregular and may suggest underlying quality issues.

5. Line 315, Fig. 4c. The FRIP is a key quality metric in scATAC-seq. The lower FRIP scores observed in HyDrop v2 (mostly between 0.3–0.4) compared to 10x v2 (most above 0.4) raise concerns about data quality and signal-to-noise ratio.

6. Line 473. The claim that HyDrop v2 footprints ‘largely correspond’ to those in 10x data lacks concrete support. The examples are scattered across several figures, making it difficult to assess. The authors may consider providing quantitative comparisons of footprint across genome, and consolidating results into a single comprehensive panel or summary figure other than from panels from different figures.

Other major concerns about the approaches

1. The paper's core scientific focus is unclear. The methods section centers on improving the HyDrop v2 platform, while the results section shifts gears to evaluating deep learning (S2F) models trained on the data. It's not clear whether the main contribution is engineering or modeling, and right now it reads like two separate papers stitched together. If the S2F performance is meant to validate the platform, the authors need to make that reasoning explicit.

2. The comparison between S2F models trained on HyDrop v2 and 10x Genomics data lacks basic experimental rigor. Model performance can be affected by architecture, training strategies, label definitions, and class imbalance—not just input data. Without proper controls or ablation, it's not convincing to attribute observed performance differences (or similarities) to the data source. A side-by-side setup with identical models, cross-dataset evaluation, and clear statistical analysis is necessary.

3. Generalization is not tested. The S2F models are trained and evaluated on HyDrop v2 data from only two settings: mouse cortex and fly embryo. If the claim is that HyDrop v2 enables training of robust S2F models, this should be tested on additional tissues, organisms, or at least some external datasets. Otherwise, it's hard to assess the practical range of the platform's utility.

4. Terminology is used inconsistently. “HyDrop v2” is sometimes the platform, sometimes the data, and sometimes the model. That's a problem. The authors should make a clean distinction between the system, the dataset (scATAC-seq profiles), and the models (S2F predictors). Right now the language adds unnecessary ambiguity.

5. The modeling section lacks key technical details. There's no architectural description, no mention of input processing, hyperparameters, or training-validation splits. For a paper that makes claims about model performance, this is not acceptable. Even though the authors used CREsted, readers still need enough information to reproduce or evaluate the results. Code and weights should be released as well.

6. There is no methodological innovation on the AI side. The authors rely on existing pipelines (CREsted, tfmodisco, seq2PRINT), and that's fine, but it should be clearly acknowledged. The value here lies in platform-driven application, not algorithm design. Right now the manuscript doesn't make that distinction clear.

7. The authors argue that the combination of deep learning's data requirements and the sparsity of scATAC-seq data necessitates large-scale datasets “across species, genotypes, conditions, and developmental stages.” However, this reasoning is not sufficiently justified. While it is true that deep learning models benefit from large datasets and that scATAC-seq data is inherently sparse, this does not logically entail that diversity across such biological axes is required. In fact, integrating data from multiple species or genotypes introduces potential domain shifts that may complicate model training rather than ameliorate sparsity or data scarcity. Unless the authors' goal is to build cross-species generalizable S2F models (which is not the stated focus here), the need for such heterogeneous data sources should be either better motivated with empirical or theoretical support, or more precisely framed as an optional advantage rather than a core requirement.

Minor Concerns
1. Figure placement and citation are disorganized. For example, Figure 1b appears after 1d, and Figure S1f is cited before S1a. Similarly, Figure 3g is never cited. Please review the entire manuscript and ensure figures are cited sequentially and clearly aligned with the text.

2. Fig. 1f. Surprisingly, 10x v1 shows higher FRIP than 10x v2. This observation needs explanation. Could this be due to batch effects, sequencing depth, or differences in preprocessing?

3. Fig. 2d. Some cell-type-specific ACRs do not show high overlap between platforms. Please comment on potential causes.

4. Line 235. The decision to forgo batch correction needs justification. Given the number of batches and platforms compared, batch effects could confound the interpretation.

5. Line 265. This paragraph lacks a visual aid reference. A figure or table should be cited to support the results presented.

6. Fig. S5a. For clarity, split the comparison plots between HyDrop v2 and 10x into separate panels, as overlapping density plots are hard to interpret.

7. Lines 313, 411. Typo noted: "Figure 43" and "Figure S73" should be corrected.

8. Lines S6a–c. The color bar scales for correlation values should be standardized across comparisons to enable direct visual interpretation.

Reviewer #2

(Remarks to the Author)
In this manuscript, Dickmänken et al. developed HyDrop v2 ATAC, which is an improved version of the HyDrop v1 published previously from the same labs. The authors showed that HyDrop v2 had improved bead barcode accuracy. The data generated by v2 is better than v1 and is comparable to the commercial 10x genomics platform. They showcased the application of HyDrop v2 ATAC by generating high-quality chromatin accessibility atlases of mouse and fly. Then they showed that the data can be used to train S2F models for biological discoveries. HyDrop v2 ATAC seems to be a useful

method and is good at generating high quality scATAC-seq data with much reduced cost, which is valuable in the current age genomic studies.

The current manuscript is a little bit confusing. When I first read the title and the abstract, I thought this manuscript is about HyDrop v2, the method. However, the main text spent too much effort on using the data to train S2F deep learning models and compared to models trained by the 10x data. The manuscript should either focus on the description of the improvements over v1 or the new findings in by HyDrop v2 data. My comments are mainly from the viewpoint of a potential user. In this case, the data quality and the easy-to-use/implement features are more important, and they are currently not sufficiently discussed in the paper. Specific comments are listed below which I hope to improve the manuscript:

1. The authors described the improvement of bead barcode generation compared to v1, which was good. The authors also claimed v2 was more sensitive than v1. Then what makes v2 more sensitive? Were there any other improvements on the ATAC chemistry? Was the improved sensitivity purely due to the improved bead barcode generation procedures? This should be clearly described.

2. For the comparisons to v1, 10x and sci-ATAC-seq shown in Figures 1f, 4c and S1b, the metrics were heavily dependent on cell types, sequencing depth and preprocessing pipelines. Are those things the same across different methods? For example, simply downloading the count matrices from the 10x genomics website and compare to the v2 data is not sufficient.

3. While common metrics like unique nuclear fragments and FRiP are good to assess the method sensitivity, they are only able to show cells that survive the droplet system. At a similar cell number, is there any distortion of cell type/subtype proportions of v2 compared to other methods? For example, by downsampling to similar numbers of cells, are there any cell clusters only present in one platform, not the other(s)?

4. From a user point of view, what exactly is the niche of HyDrop v2 ATAC and when should I use it? The lower cost is probably one factor to consider, but this is only true for labs who can build or already have the droplet device. In addition, sample multiplexing like txci-ATAC-seq and scifi-ATAC-seq would also reduce cost, which seems to be an easier and more straightforward way of lowering the cost. For a broader audience, please provide some general guidance on choosing HyDrop v2 and the commercial 10x genomics.

5. The authors claimed that the 110k mouse motor cortex atlas cost 1080.6 euros with v2, and would have cost 1398.29 euros with v1 and 15,330.70 with 10x. A breakdown of the cost per reagent to see where the major cost comes from would be helpful.

6. The authors showed that the Trithorax motif was successfully detected by models from HyDrop ATAC v2 and sci-ATAC, but not 10x. I'm not sure how to interpret the result. First, the authors pointed to Figure 5i to support this claim, but it is not entirely clear where I'm supposed to look at. Is it the label "Tri" that we should be focused on? Some highlight would be helpful. In the position labelled "Tri", some bases have positive scores, though fairly small compared to the Grh ones, in the HyDrop v2 and sci-ATAC tracks, and some bases have negative scores in the 10x track. Is this the evidence? The figures were blurry and I'm unable to read the small logos after zooming in. Some explanation would be nice. Second, perhaps more important, could the authors speculate and discuss why the motif is missed in the 10x model? It cannot be data quality, since the quality of the date sets were comparable. Was it due to cell numbers or something else? This is where some knowledge can be learned.

7. The authors also claimed the droplet generated in house is better than Atrandi, and points to Figure S1d,e. However, Figure 1d is a table with numbers. Neither the main text nor the figure legend explained what the expected numbers would be, and why those numbers tell us that the in-house one is better than the Atrandi one.

8. The writing of the manuscript felt rushed. There are many texts with unclear logic, some poor choices of reference and some typos, all of which make the reading not very pleasant:

8.1) Line 46: the authors use Ref1 (Buenrostro2015) to support the claim that cell type identify is encoded in CREs, which is a lazy choice. It was the paper that invented scATAC-seq, but it is not the first to demonstrate that cell identity is encoded in enhancers. I'm sure there are better choices to make this point.

8.2) Lines 54 and 56: when the authors introduce scATAC-seq, only Buenrostro2015 was cited, but people generally think it was Buenrostro2015 and Cusanovich2015 (doi: 10.1126/science.aab1601) together invented scATAC-seq, and they should be cited together.

8.3) The authors claimed to have 7 batches of beads for the v2 benchmarking shown in Figures 1f, S1b, S1d. They sometimes were labelled A,B,C,D,E,F,I and sometimes bathes 1,2,3,4,5,6,7. I assume they are the same? Better make them consistent.

8.4) Many figures and figure panels were poorly organised. For example, in line 127, after Figure 1a was mentioned, Figure 1d was followed. Figure 1b is not referenced at all in the main text, and Figure 1c is referenced after Figure 1d. Similarly, in line 171, Figure 1e was followed by Figure 1h, and Figures 1f and g were only referenced later. In line 194, Figure 1e was followed by Figure 4a. In line 313, Figure 43 was mentioned. I did not list all of them, please check and reorganise the figures, so that the readers will not jump back and forth.

8.5) Both sciATAC and sci-ATAC were used. Please be consistent.

8.6) In Figure S1g, there is no data after 700bp for 10x v2, no data after 400 bp for HyDrop. Are those correct?

8.7) Marker gene names should be in italic format. For example, genes mentioned in lins 531, 532 and 1157. I did not list all of them.

Reviewer #3

(Remarks to the Author)
In Dickmänken et al, the authors benchmark an improvement on their previous technology (Hydrop v1, De Rop 2022) and use it to perform snATAC-seq and machine learning on the subsequent accessible chromatin profiles. Hydropv1 had already introduced the lower pricing, but had problems at the level of the barcode stability and complexity. This improved method, which addresses these previous limitations, is meant to be both a time-saving and cost-saving technology compared to commercial platforms (10x Genomics, primarily) and to other opens source methods such as sciATAC. As per it's name, Hydrop belongs to the microfluidic, bead-based methods for single-nuclei capture and barcoding. The authors combine improvements in their previously developed technology with previously developed machine-learning algorithms to train S2F models of cell-type specific CREs. As such, the of the major strength of this work is to 'democratize' the use of snATAC at a more reasonable cost which could open its use for more groups and to more cell types/conditions as scale can be achieved cheaply without compromising quality. The work proposes a series of elegant benchmarks by which to compare Hydropv2 vs other methods to show their equivalency, both in mouse brain and in the Drosophia embryo. The comparison of Hydropv2 to the 10x platform is well done and the use of it to train S2F models where large datasets are necessary underscores the importance of having scalable technical platforms. In the Drosophila the manuscript nicely shows that the generated data + the S2F models can recapitulate in vivo enhancer activity with their predictions, as well as being able to predict synthetic enhancer activity in specific tissues. The authors show the power of using S2F models which contain all the cell-types to predict the specificity (or not) of an enhancer. Despite all these positive points and the well-done work, the manuscript doesn't reflect a major advancement in how snATAC is either performed or analyzed on the technical level—rather the major advancement is in price/nucleus. Codes, protocol and data are nicely provided, making it a good, open-access reference source.

Some aspects of the manuscript could be improved for clarity by figure and associated text:

Figure 1:
- For clarity – in Figure 1, is the 10x data from the 10x Genomic's website used (cited in the data availability section) and the data from BICC data used? Also reference to Refs #12 and 24 which have additional datasets.
- 1h – I'm not sure that I agree there is no batch effect. The effect doesn't overwhelm the cell type specific features that cause clustering, but those are generally driven by a clear set of open enhancers. However, it's also clear that we don't end up with a even distribution of beads between the different clouds, and the paper doesn't analyze, to my recall, how the peak calls very per bead batch. I assume the seven batches were tested with the same pool of cells, but this was not super clear on first reading.

Figure 2:
• For the purposes of what Fig2a is meant to show, I think it should also be shown colored by experiment type; i.e. put S2A in the main text, as that's the integration that actually makes the point, not the one colored by cell type. In S2A, we see a population that is captured only by 10x and the HydropV1. Ideas about that this is?
• Do you capture the same cell types – my feeling was that is was predominantly a problem of the labeling discrepancy between the 10x dataset and the Hydrov2 dataset.
• I find it not intuitive that the scaling on the x-axis is not the same for the chosen DAR examples. Also, would It be possible to add called peaks from macs as an additional bar above each sample. This would give us a better idea if the data is generating similar MACs peak calls and thus would yield similar downstream results.
• For all plots with "percentage overlap of DARs" – it wasn't clear to me what the percentage overlap is based on. Start and end sites of the peak? Signal in peak? Genes it's annotated to? For 2d, in cell types with less strong concordance, is there are relationship with the number of cells captured (for example the L5_IT).

Figure 3:
- For clarity in reading, I think it could be useful to point out in the text how many nuclei/cells were used for the BICCN model using the 10x data to avoid having to cross-reference a lot of papers.
- Possibly a rendering problem, but Figure 3f is difficult to read because of the color choice.
- Reference to panel 3g is missing from text, and from legend how to interpret the heatmap was not exactly clear. Presumably related to the predictions use to calculate the AP and related to 3f.
- Possible edit for clarity: 3j is referenced in the main text in the context of the TF footprints, which are been assessed in the mouse and the Drosophila. I think thiis is referred two in the lines 268-271 which corresponds on the mouse section. Consider moving all the foot printing together?

Figure 4
- Consider adding Figure A from S5. This is the data that help us understand claims about the batch effects. Same comments as the mouse data apply here, except it's much easier to see as you have comparable numbers of nuclei. Since

here you are really starting with the same samples, it would be possible to do an estimation of cell-type capture per technology.
- There is a change in the efficiency of the sequencing (i.e HyDropv2 needs more reads to get to the same depth). Within the error of the 10x this doesn't seem meaningful to me (and from a cost perspective is not important) but I am more curious is you have a sense of why. I ask this in the case of thinking about "non-ideal" tissues – i.e. postmortem samples from patients, surgical discard etc. where already the noise tends to be larger and the data quality decreases.
- The scoring of 4f is generally better than in the mouse, suggesting that really the differences are more experiment to experiment (as the mouse data was not collected at the same time as the mouse Hydrop) and highlights the concordance of the data nicely. There is about 80% overlap in the two technologies on average, which is great. Do the authors have a sense about what the 20% non-overlapping represents? Technical noise, differences in the Tn5 and other reagents etc?

Figure 5:
- Are the 340,604 nuclei from 5A the same as from 4b? The difference essentially is the projection with or without 10x data in the same space?
- From the lengend, a little hard to interpret the data related to the Kvon et al data (how is the ground truth in 5f scored?)
- Line 445-446: "Possible variations between models can be attributed to model variability, as shown in the 10-fold cross-validation (Figure 5d)" – circular sentence; I think the authors means that models trained on both datasets have similar confidence intervals and thus accuracy ranges?

Reviewer #4

(Remarks to the Author)
I co-reviewed this manuscript with one of the reviewers who provided the listed reports. This is part of the Nature Communications initiative to facilitate training in peer review and to provide appropriate recognition for Early Career Researchers who co-review manuscripts.

Version 1:

Reviewer comments:

Reviewer #1

(Remarks to the Author)
The author has addressed most of my concerns.

Reviewer #2

(Remarks to the Author)
The authors have taken significant effort to reorganise the manuscript. My previous concerns were mainly about the writing the technical aspects of Hydrop V2 itself which have now been successfully addressed. Therefore, I have no further concerns.

Reviewer #3

(Remarks to the Author)
The authors have adequately addressed my concerns and have improved the clarity and the readability of the text and the figures, as well as the usability of the techniques they discuss.

Reviewer #4

(Remarks to the Author)
I co-reviewed this manuscript with one of the reviewers who provided the listed reports. This is part of the Nature Communications initiative to facilitate training in peer review and to provide appropriate recognition for Early Career Researchers who co-review manuscripts.

Open Access This Peer Review File is licensed under a Creative Commons Attribution 4.0 International License, which permits use, sharing, adaptation, distribution and reproduction in any medium or format, as long as you give appropriate credit to the original author(s) and the source, provide a link to the Creative Commons license, and indicate if changes were made.
In cases where reviewers are anonymous, credit should be given to 'Anonymous Referee' and the source.
The images or other third party material in this Peer Review File are included in the article's Creative Commons license, unless indicated otherwise in a credit line to the material. If material is not included in the article's Creative Commons license and your intended use is not permitted by statutory regulation or exceeds the permitted use, you will need to obtain permission directly from the copyright holder.
To view a copy of this license, visit https://creativecommons.org/licenses/by/4.0/

REVIEWER COMMENTS

Reviewer #1 (Remarks to the Author):

This study introduces HyDrop v2, an improved, open-source microfluidic droplet platform for scATAC-seq. Compared to commercial systems, HyDrop v2 enhances sensitivity, reproducibility, and cost-efficiency, enabling the generation of large-scale single-cell chromatin accessibility atlases required for training S2F deep learning models. The method represents a valuable, accessible alternative to commercial platforms, particularly for labs with limited resources. However, several critical issues need to be addressed before further consideration. For example, the current version lacks a clear scientific narrative, does not control for key confounders in the AI evaluation, and misses the level of rigor expected for claims about model performance. The work could be valuable if reframed as a carefully controlled benchmark study of platform-generated data for AI tasks. To reach that level, it needs tighter experimental design, cleaner definitions, and more disciplined analysis.

We thank Reviewer 1 for the constructive suggestions. We significantly rewrote the manuscript, improved the narrative, added many additional analyses, and focused more on a carefully controlled benchmark study as suggested. We now trained models with 10-fold cross-validation to address model variability. We believe that our new structure provides a clearer story and we hope that the reviewer agrees.

Major Concerns

1. On line 192. It is unclear whether the authors ensured a fair comparison between methods regarding cellular diversity. Given that more cells were processed with HyDrop v2, it is unsurprising that more diversity was captured. The authors should explicitly state whether subsampling or normalization was performed to ensure comparability. A controlled analysis showing that HyDrop v2 captures equivalent diversity to 10x v2 given the same number of cells would be more convincing.

We have addressed this concern by subsampling the mouse cortex data to only focus on M1 motor cortex. We trained models with identical model architectures and hyperparameters on HyDrop v2- and 10x-based data, and we varied the training data in terms of the number of cells and the number of reads in the preprocessed data. For each scenario, we performed baseline model training on all peaks, followed by a fine-tuning stage on cell-type-specific peaks. Additionally, we performed k-fold cross validations (k=10) for each model, wherein each fold model was validated on different test regions, ensuring the same train-test splits between HyDrop v2- and 10x-based data. When investigating these models with respect to the inputs, such as in the case of the sequence contribution plots, or, when validating on different datasets, we used the average predictions over the ten folds to reduce variability.
In total, we trained 220 models starting from the same hyperparameters (11 scenarios x fine tuning and baseline x 10 folds). Reducing the number of cells of the training data set mainly impacts the average performance across all predictions (macro AP), while reducing the sequencing depth per cell does not impact the precision and recall (Fig. S3a). Based on these findings, we determined the number of additional HyDrop v2-based cells needed to generate a model comparable to one trained on 10x-based data (Fig. 2d).

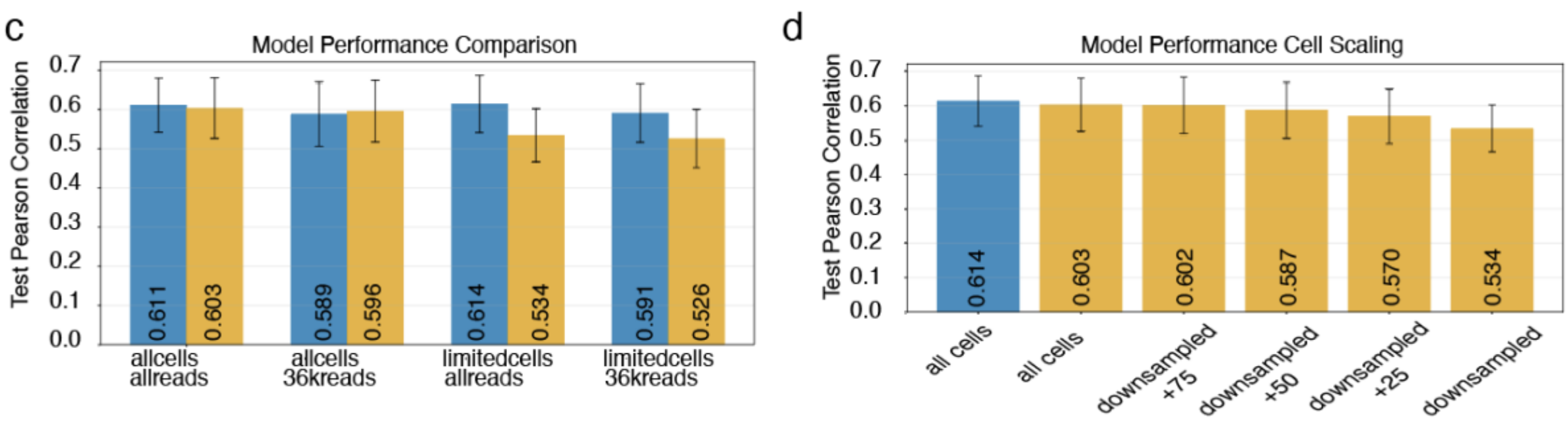

In addition to model performance parameters, we evaluate how well the S2F models trained on different datasets predict experimentally-validated enhancer sequences as active (Fig. 2b). The S2F models trained on the data at full read depth (Fig. 2e) or downsampled to 36kRPC (Fig. S3b) score comparably between 10x-based training data and HyDrop v2-based training data, both at the full and reduced number of cells. Thus, even though we see small differences in model performance (Figs. 2b-c), all S2F models trained on different scenarios of HyDrop v2-based data identify genomic enhancer sequences that were previously validated as active enhancers *in vivo.*

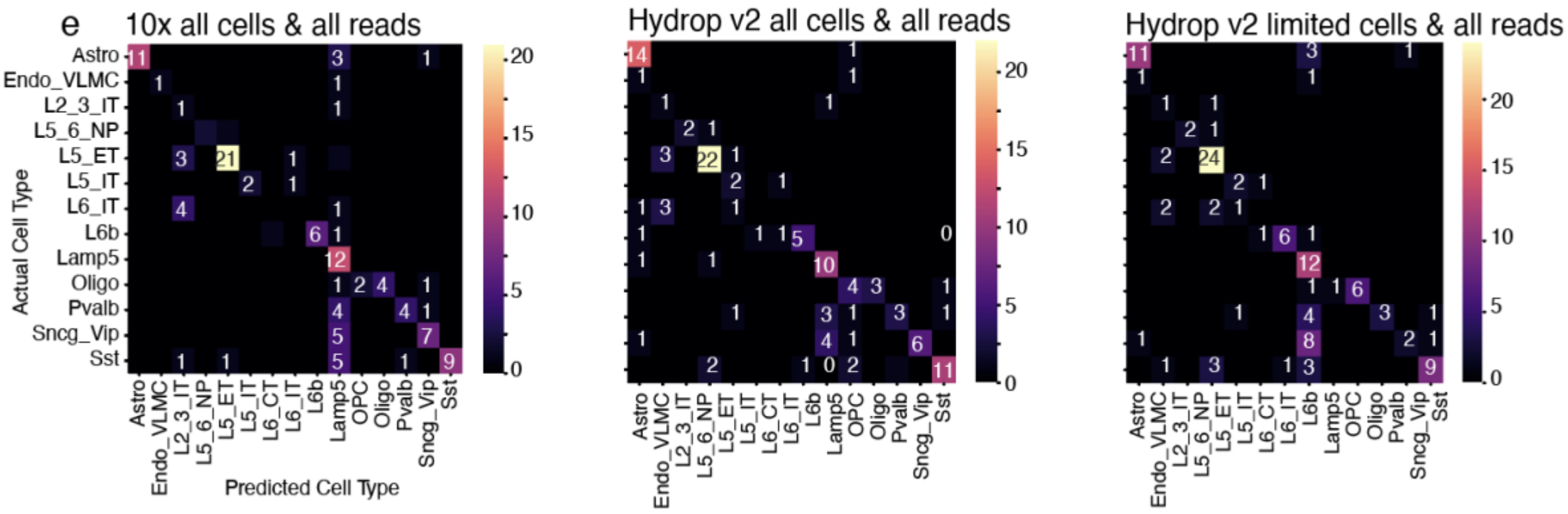

For fly, no dissection was performed, and whole animals were processed, making a straightforward comparison of the same cell types possible, as already shown in the first version of the manuscript. Here, the HyDrop v2-based dataset (340,604 cells) contains 27% more cells than the 10x-based dataset (266,726 cells) while both datasets are sequenced comparably deep (10x v2: Mdn=13.21kRPC, SD=2.09; HyDrop v2: Mdn=15.88kRPC, SD=6.81). Following our insights on in-depth model comparisons applied to the mouse motor, we focused on training two identical models with the full data set and read depth for both 10x- and HyDrop v2-based data, each with 10-fold cross-validation. Overall, both fly embryo models score identically in model performance and enhancer identification.

2. Lines 215 & 307, Fig. 2b & 4d. The current figure 5cs only show examples at single loci, which are not representative of genome-wide performance. The authors may consider includING genome-wide comparisons of DARs and fragment distributions, and reporting the total number of DARs and TFBS identified by each method. Extend the comparison beyond isolated regions and include summary statistics across the genome.

The carrot plots in Figures S2e and g show a genome-wide comparison of fragment coverage in DARs accessible in various cell types. We decided to also keep the examples of single loci for illustration. The correlation of TFBS/motifs identified by motif enrichment between HyDrop v2 and 10x-based data is shown in Figure S2h and i, to also include an in-depth sequence comparison that is not based on S2F models. In addition to the fragment coverage at the genome-wide level, we included the genome-wide cut sites (insertion position of the Tn5 enzyme in the accessible chromatin, see Figs. 5b-c, S7a-b); a more detailed explanation is given in Question 4.

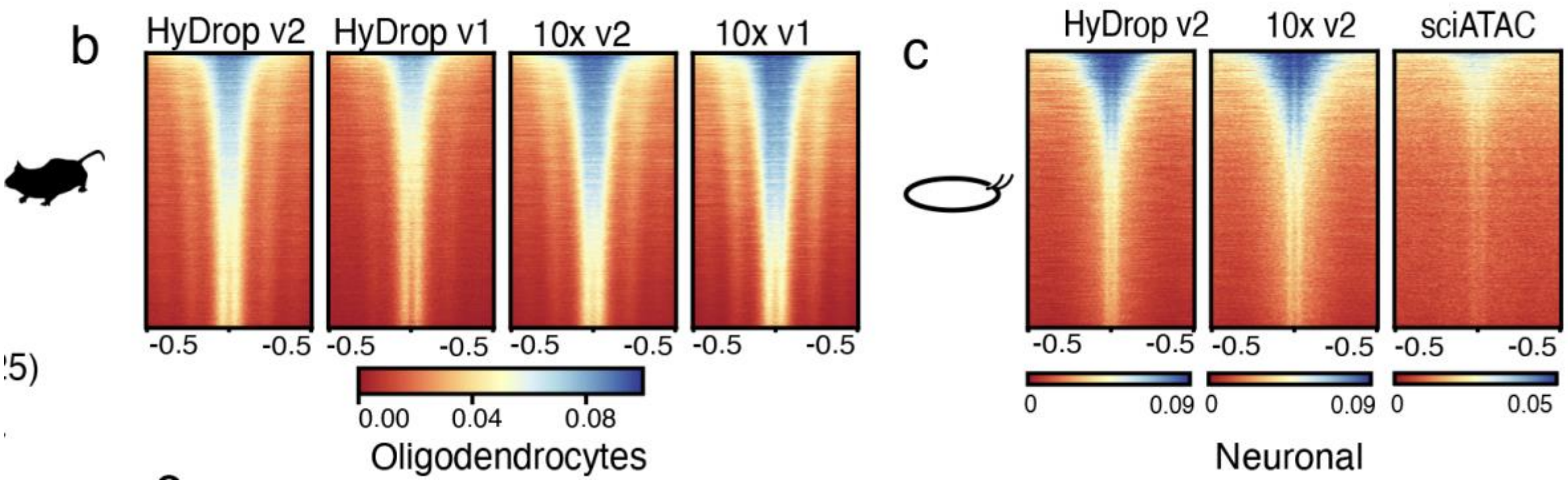

Figure 5b-c

3. Lines 240–241. While the authors claim that HyDrop v2 models perform similarly to those based on 10x data, the visual evidence (Figure 3b) suggests that the 10x model performs better in certain classes. The authors should acknowledge this difference and provide quantitative metrics to support or qualify their claims.

We refer to our answer to Question 1.
We acknowledge in the manuscript (Results section 1) that the individual per-cell fragment count (reflecting the sensitivity of the assay) is lower for HyDrop compared to 10x with HyDrop v2 samples reaching 93.8% of the unique fragments (log) count of 10x v2-based data on average at a sequencing depth of 36k reads per cell. Nevertheless, the S2F models are either equivalent (for fly) or nearly equivalent (for mouse), and by simply adding more cells in HyDrop (which is easy and cost-effective), any small advantage of 10x can be easily compensated. We believe that this is a very important finding and message, because for research projects that aim to generate training data for ML models, custom assays that are more cost-effective provide equivalent insights. We explain this in the Results and Discussion, as it represents the crux of the paper.

4. Line 308, Fig. S5d. The HyDrop v2 peak within the hemocyte data differs notably from 10x v2, with an apparent lack of read coverage in specific regions. This discrepancy warrants further investigation—are these technical artifacts, biological variability, or data quality issues? Additionally, the sciATAC profile appears irregular and may suggest underlying quality issues.

There are indeed differences in coverage observed on the individual locus level between the techniques. Using the same genotype and collection window should minimize the biological effect in the fly embryo data. To minimize the effect of technical artifacts, we also show the genome coverage in differentially accessible regions per cell type (see Question 2) that show a close resemblance between 10x and HyDrop v2-based data.

For sciATAC-seq, we agree that the differences are much more pronounced. Particularly, sciATAC-seq profiles show a much wider fragment coverage that is clearly visible across individual loci (shown for somatic muscles differentially accessible region (DAR)):

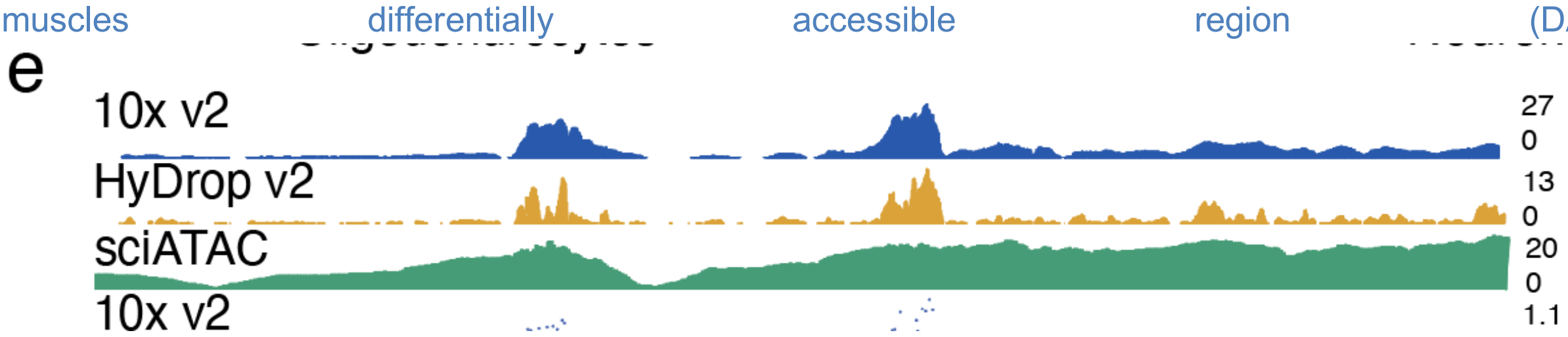

On the genome-wide level, this is also reflected in the carrot plots (Figs. 2c), and by the fragment size distribution, showing much larger fragments in sciATAC data (Fig. Sf).

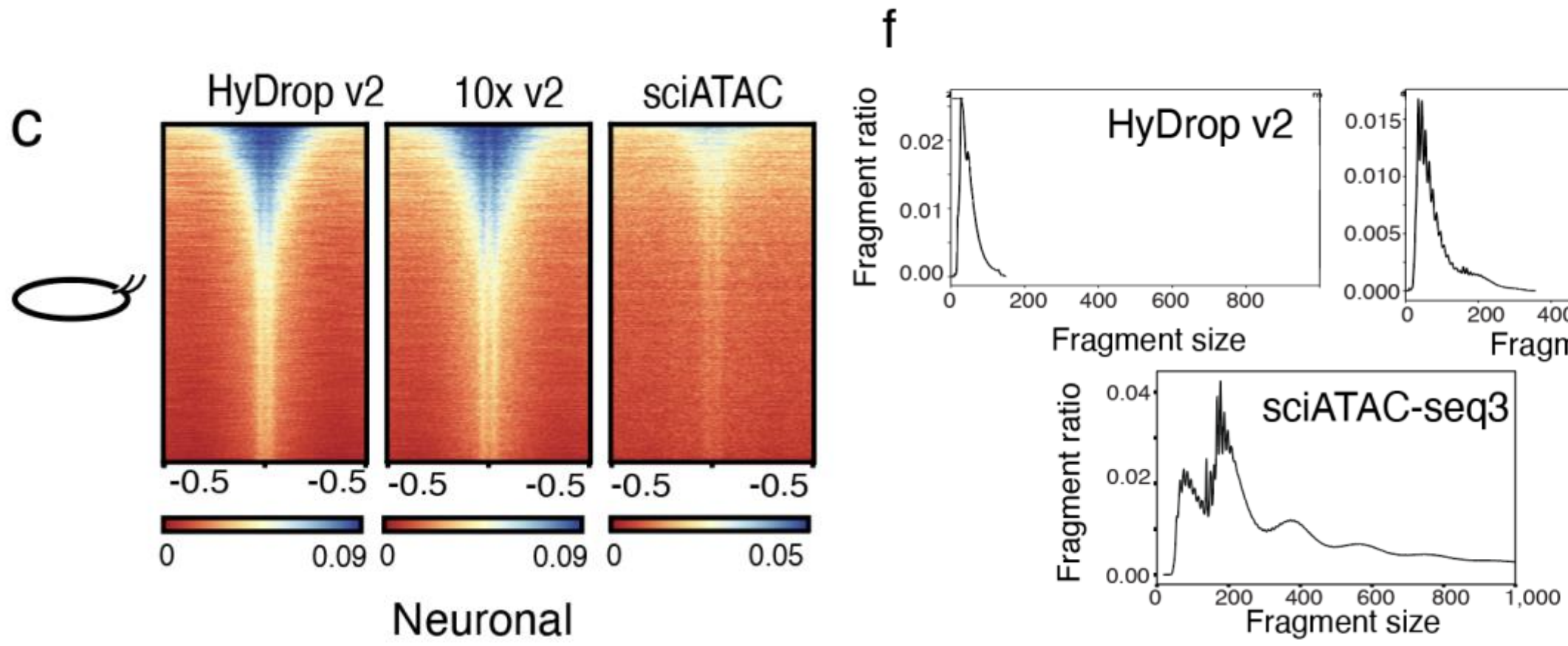

We discovered that the individual insertions of Tn5 (cutsites) of sciATAC-seq better resemble 10x and HyDrop v2-based data (to our knowledge, this has not been shown before). Cut sites for somatic muscles DAR shown below:

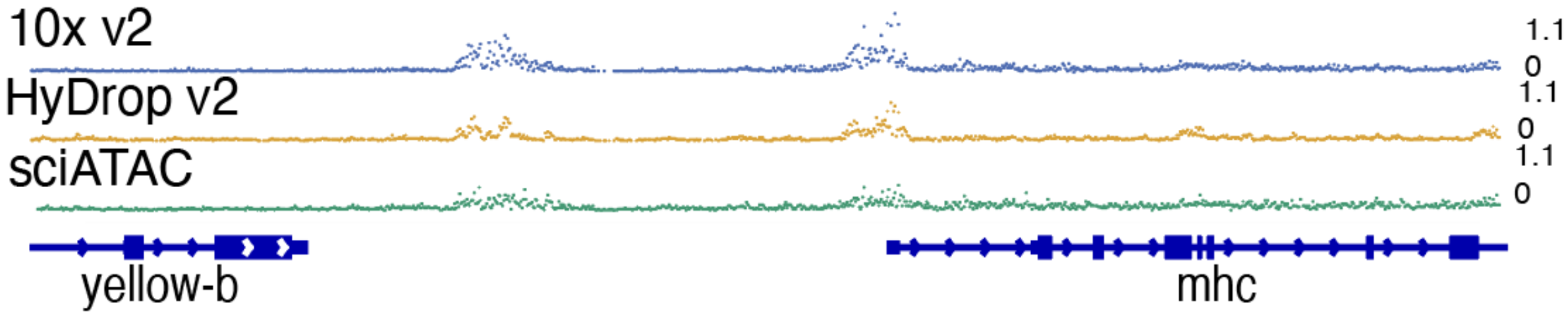

In other words, we find that the obvious quality issue in sciATAC data can be partially mitigated by analyzing the cut sites instead of the coverage information. For the mouse, the differences between fragment sizes are not as outspoken (Fig. S7e).

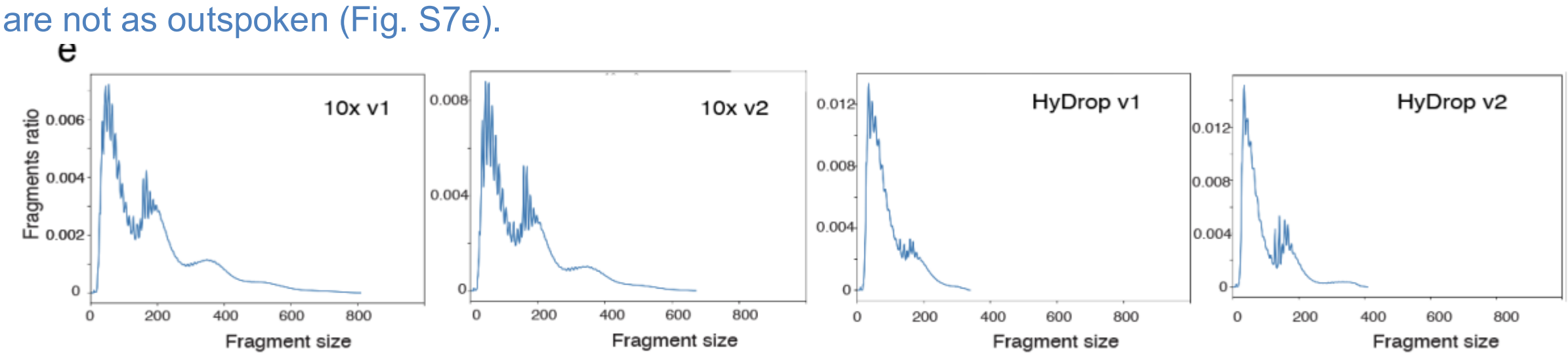

5. Line 315, Fig. 4c. The FRIP is a key quality metric in scATAC-seq. The lower FRIP scores observed in HyDrop v2 (mostly between 0.3–0.4) compared to 10x v2 (most above 0.4) raise concerns about data quality and signal-to-noise ratio.

There might be a slight misunderstanding. We do not aim to prove that HyDrop v2 has similar QC metrics compared to 10x. The commercial 10x kit is highly optimized and yields the highest quality data. However, we show here that once a cheap, custom method like HyDrop v2 reaches a critical sensitivity and quality (for example, compared to an earlier 10x version), it yields equivalent atlas-level quality and equivalent biological insight, particularly when the data (HyDrop or 10x) is used to train S2F models. Therefore, such S2F models and consequent biological insight can now be achieved at a much-reduced cost, and by any research lab, with limited resources (up to 14x reduced: 9,337 euros for 67k cells with 10x v2 platform divided by 658 euros for the same amount of cells by HyDrop v2 platform). We believe that this is a very interesting new finding and will help the field to build large, organism-wide atlases or large-scale perturbation atlases that are not achievable by individual labs using the commercial kits.

Having said that, it is still interesting to observe that HyDrop-v2 has much improved in the basic QC metrics: Compared to the previously published HyDrop v1 atlas of the mouse cortex, we observe a 9.14% increase in the recovered unique fragments (log), now reaching 93.8% of the unique fragments (log) count of 10x v2-

based data on average at a sequencing depth of 36k reads per cell (RPC, Fig. S2a). In the fly embryo atlas, HyDrop v2-based data also reaches on average 94.1% of the unique fragments (log) count compared to 10x v2-based data at 12kRPC (Fig. S2b).
Regarding the FRIP in mouse data, at 36kRPC, HyDrop v2 data reaches 97.01% (M=.5, SD=.09) of the FRIP of 10x v2 data (M=.53, SD=.09), while HyDrop v1 only reaches 81.8% (M=.43, SD=.1) of the 10x v2 FRIP score.
Additional numbers can be found in the supplementary note, where we summarized the improvement steps taken and the key QC metrics in mouse and in fly. By focusing on evaluation metrics beyond the classical quality metrics and actually using DL tools, we introduce a benchmarking that is closer to the research reality and highlights that training DL models is possible on cost-effective custom-made platforms, democratising research.

6. Line 473. The claim that HyDrop v2 footprints 'largely correspond' to those in 10x data lacks concrete support. The examples are scattered across several figures, making it difficult to assess. The authors may consider providing quantitative comparisons of footprint across genome, and consolidating results into a single comprehensive panel or summary figure other than from panels from different figures.

Thank you for this suggestion, we agree. We added scatterplots showing the correlation of footprints across all validated enhancers in the mouse motor cortex and in the fly embryo (Figure 5f-g).

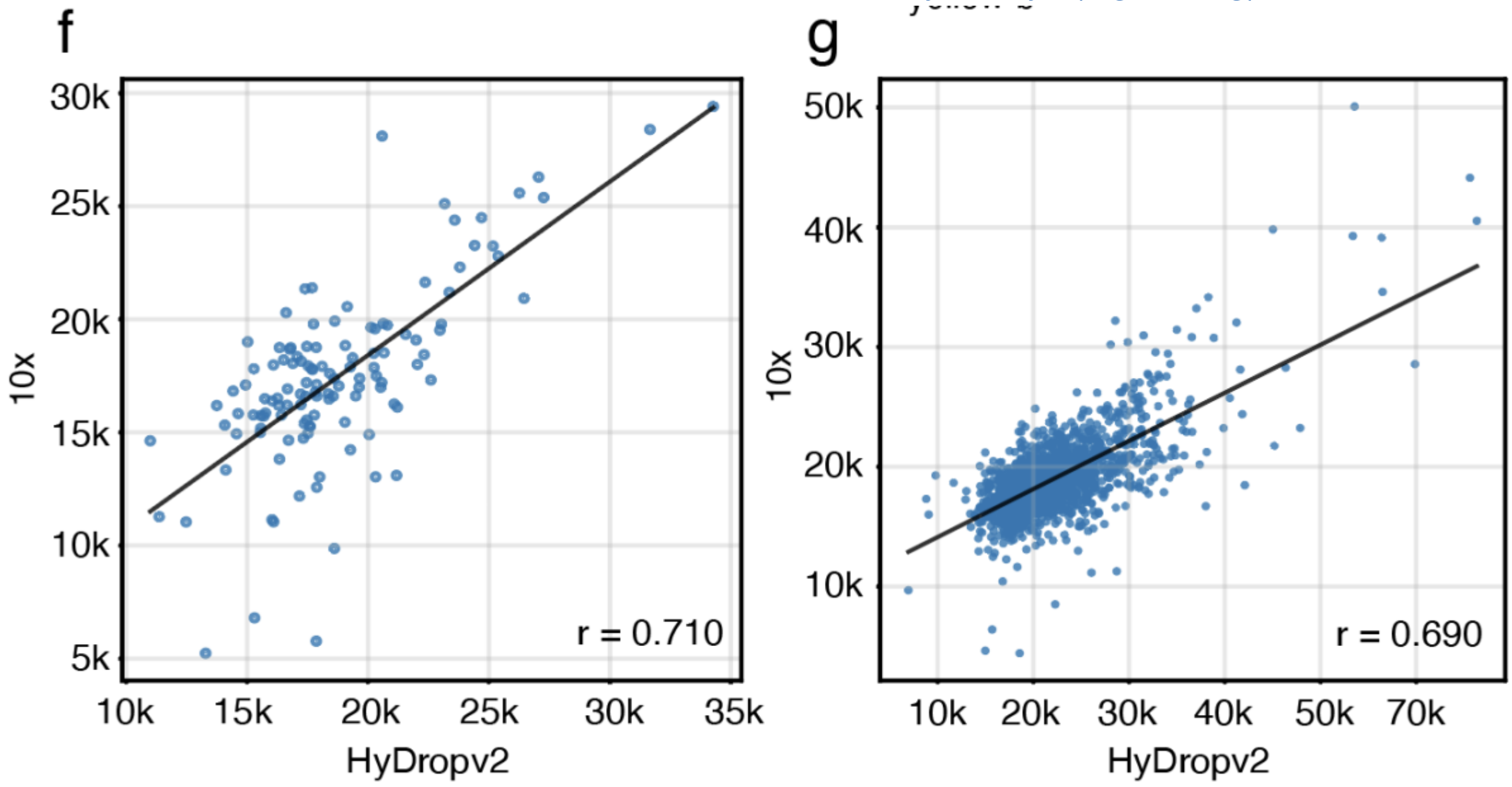

Other major concerns about the approaches
1. The paper's core scientific focus is unclear. The methods section centers on improving the HyDrop v2 platform, while the results section shifts gears to evaluating deep learning (S2F) models trained on the data. It's not clear whether the main contribution is engineering or modeling, and right now it reads like two separate papers stitched together. If the S2F performance is meant to validate the platform, the authors need to make that reasoning explicit.

Thank you for the valuable feedback. We have rewritten the paper following this suggestion, changed the title, and created a better narrative. The main message is that new data generation technologies can be benchmarked using AI models (this is, to our knowledge, the first time that this is shown). This message has also become stronger by extending the deep learning section with quantitative comparisons of 220 models. In addition, we present an improved HyDrop-ATAC protocol (less focus, more in Methods and supplement), and apply the AI-based benchmark to compare HyDrop-ATAC with 10x Genomics. We believe that the two fit very well together, and we hope that the reviewer finds the scientific focus clearer in the

revised version. Our focus is to synergize between tech development and sequence-to-function AI models, and to make the generation of S2F training data more accessible for a broader audience.

2. The comparison between S2F models trained on HyDrop v2 and 10x Genomics data lacks basic experimental rigor. Model performance can be affected by architecture, training strategies, label definitions, and class imbalance—not just input data. Without proper controls or ablation, it's not convincing to attribute observed performance differences (or similarities) to the data source. A side-by-side setup with identical models, cross-dataset evaluation, and clear statistical analysis is necessary.

To address the model imbalance, we focused on re-sampling the mouse data with (1) focusing on M1 cell types, (2) include the same amount of cells across techniques, (3) compare 10x with HyDrop v2 with the same protocol of data generation and taking out the earlier data from Johansen et al. (2024, 10.1101/2024.08.21.609075), (3) train models on downsampled reads and full sequencing depth for a full comparison of S2F parameters. For each scenario, we performed baseline model training on all peaks, followed by a fine-tuning stage on cell-type-specific peaks. Additionally, we performed k-fold cross validations (k=10) for each model and used the average predictions over the folds of the finetuned models in our analyses. In total, we trained 220 models starting from the same hyperparameters (11 scenarios x finetuning and baseline x 10 folds).
For the fly data, the HyDrop v2-based dataset (340,604 cells) contains 27% more cells than the 10x-based dataset (266,726 cells) while both datasets are sequenced comparably deep (10x v2: Mdn=13.21kRPC, SD=2.09; HyDrop v2: Mdn=15.88kRPC, SD=6.81). Following the previous insights of the mouse motor cortex in-depth model comparison, we focused on training two identical models with the full data set and read depth for both 10x- and HyDrop v2-based data, each with 10-fold cross-validation as eluded in Question 1 of the first section.

3. Generalization is not tested. The S2F models are trained and evaluated on HyDrop v2 data from only two settings: mouse cortex and fly embryo. If the claim is that HyDrop v2 enables training of robust S2F models, this should be tested on additional tissues, organisms, or at least some external datasets. Otherwise, it's hard to assess the practical range of the platform's utility.

We respectfully disagree that our findings, obtained on two very different biological systems and species, would require additional tissues or organisms, in the same paper, to support our conclusions. To apply the same comparison on additional tissues or organisms would require a significant effort, and when we compare to other papers that present new technologies, or papers that benchmark technologies (e.g., Johansen et al. (2024), Pampari et al. (2025), De Rop et al. (2023), Hu et al. (2025), Hingler et al. (2024)), we already go significantly beyond, with two rather than 1 species/system, and with an entirely new fly embryo atlas with >600K cells being the largest embryo atlas to date of *Drosophila melanogaster*.

4. Terminology is used inconsistently. "HyDrop v2" is sometimes the platform, sometimes the data, and sometimes the model. That's a problem. The authors should make a clean distinction between the system, the dataset (scATAC-seq profiles), and the models (S2F predictors). Right now the language adds unnecessary ambiguity.

For additional clarity, we refer to the platform as "HyDrop v2", while the data as "HyDrop v2 data" and the model as "HyDrop v2-based model".

5. The modeling section lacks key technical details. There's no architectural description, no mention of input processing, hyperparameters, or training-validation splits. For a paper that makes claims about model performance, this is not acceptable. Even though the authors used CREsted, readers still need enough information to reproduce or evaluate the results. Code and weights should be released as well.

Thanks, we have incorporated important details of data preprocessing and model training into the results section 2. The details can be found in the Methods.
Briefly, for mouse, we trained models with an identical model architecture and identical hyperparameters on HyDrop v2- and 10x-based data consisting of several experiments with varying numbers of cells, and number of reads in the preprocessed data. Additionally, we performed k-fold cross validations (k=10) for each model. Each chromosome was split into k equal parts and identical train and test regions per fold were used between Hydrop v2- and 10x-based data. For each scenario, we performed baseline model training on all peaks, followed by a finetuning stage on cell-type-specific peaks. Average performance metrics were then calculated over the test regions per finetuned fold model. When validating on external datasets we used the average predictions over the folds of the finetuned models in our analyses.
The code for all computational analyses is now available at https://github.com/aertslab/HyDrop_v2_paper, and all trained models and preprocessed data files can be found on Zenodo.

6. There is no methodological innovation on the AI side. The authors rely on existing pipelines (CREsted, tfmodisco, seq2PRINT), and that's fine, but it should be clearly acknowledged. The value here lies in platform-driven application, not algorithm design. Right now the manuscript doesn't make that distinction clear.

Agreed, we used existing S2F modeling frameworks (CREsted), architectures, TF footprinting methods (seq2PRINT), and motif discovery methods (pyCisTarget). We acknowledge this and reference them. Also in relation to Question 5 above, we added more details on the model itself in the text (while referring to the CREsted by Kempynck et al. (2025) and footprinting paper by Hu et al. (2025)). Additionally, Figures 2b and 4b depict the architecture of the models while also referring to the CREsted paper (logo) while we refer in Figure 5 directly to seq2PRINT paper as well.

REDACTED

Figure 2b

REDACTED

Figure 4b

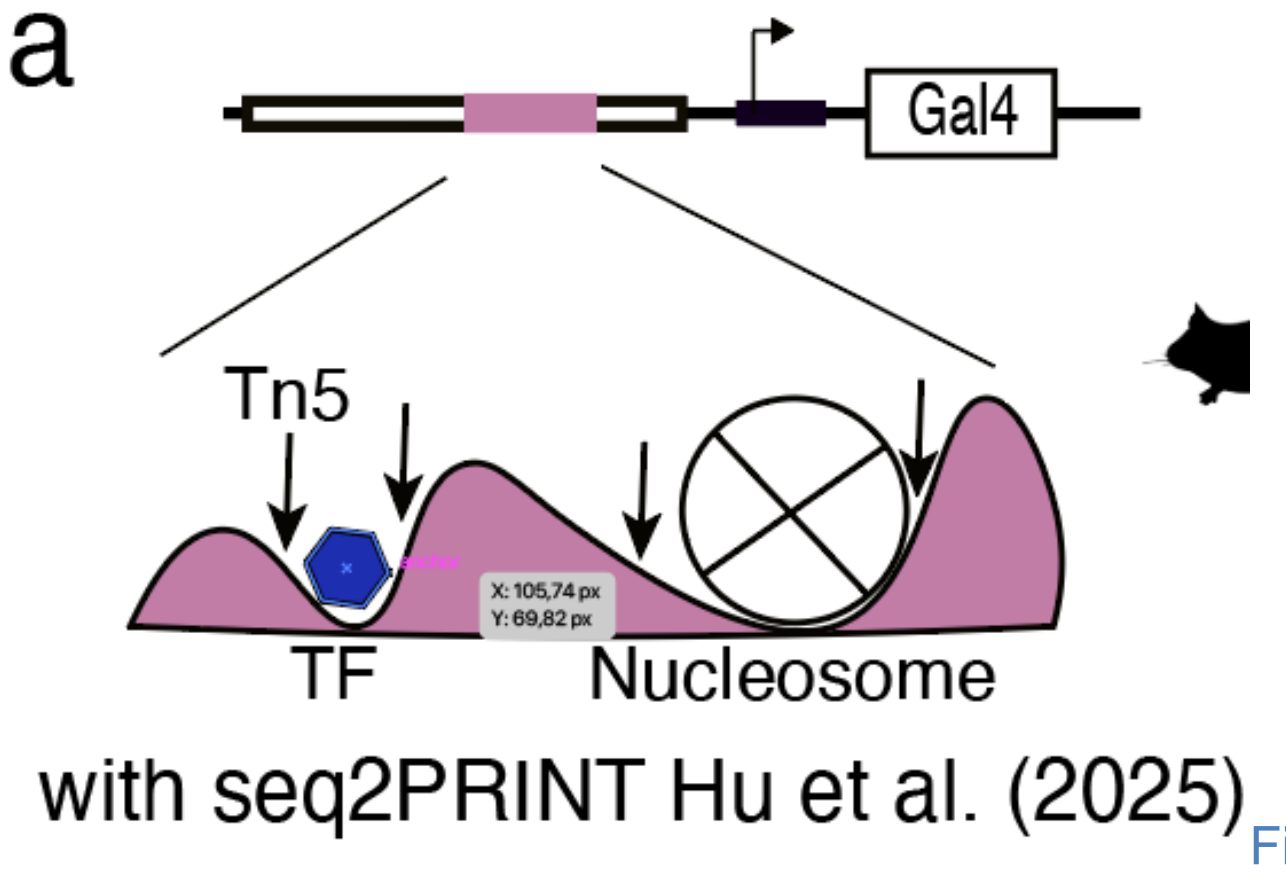

Figure 5a

7. The authors argue that the combination of deep learning's data requirements and the sparsity of scATAC-seq data necessitates large-scale datasets "across species, genotypes, conditions, and developmental stages." However, this reasoning is not sufficiently justified. While it is true that deep learning models benefit from large datasets and that scATAC-seq data is inherently sparse, this does not logically entail that diversity across such biological axes is required. In fact, integrating data from multiple species or genotypes introduces potential domain shifts that may complicate model training rather than ameliorate sparsity or data scarcity. Unless the authors' goal is to build cross-species generalizable S2F models (which is not the stated focus here), the need for such heterogeneous data sources should be either better motivated with empirical or theoretical support, or more precisely framed as an optional advantage rather than a core requirement.

Yes, we agree. We do not focus on building cross-species generalizable S2F models. There are two aspects we find important related to the 'large data' requirement. Firstly, minimal data quality and number of cells are needed in order to properly model enhancer logic using chromatin accessibility data. In earlier work, we have quantified this as 100-200 cells (Hecker & Kempynck, Science 2025), and also alternative methods like ChromBPNet have shown that a minimum of (pseudobulked) coverage is needed (for example, 5 million reads in total, cumulative across all pseudobulked cells). For rarer cell types (for example, layer 5 ET neurons in the mouse cortex), the total atlas needs to be large enough before enough cells are obtained for the rarer cell types. The second aspect of this statement relates to the breadth of deciphering the genomic regulatory code. The S2F modeling field has seen great success across a variety of cell types and systems. It is a promising avenue to have access to *all* cell types of an animal, including all developmental states. For a fruit fly, this would quickly amount to more than 5 million cells, and for a mouse, this would be at least an order of magnitude larger.

Minor Concerns

1. Figure placement and citation are disorganized. For example, Figure 1b appears after 1d, and Figure S1f is cited before S1a. Similarly, Figure 3g is never cited. Please review the entire manuscript and ensure figures are cited sequentially and clearly aligned with the text.

We adapted the flow of the paper and carefully matched the figure order to the text in both main and supplementary figures.

2. Fig. 1f. Surprisingly, 10x v1 shows higher FRIP than 10x v2. This observation needs explanation. Could this be due to batch effects, sequencing depth, or differences in preprocessing?

In the previous version 10x v1 (M=.58, SD=.11) indeed showed a higher FRIP than 10x v2 (M=.53, SD=.09). The 10x v1 samples downloaded from the 10x genomics website include, in contrast to the in-house and downloaded 10x v2 samples, tissue that is processed when fresh instead of frozen. We observed in other experiments as well that fresh tissue tends to outperform frozen one, but the experimental process is more difficult to organise when dissection needs to be immediately followed by sample preparation for sequencing. Due to the differences in library preparation, we opted to compare the more recent 10x v2

platform to HyDrop v1 and HyDrop v2. For additional clarity regarding key QC metrics, we added the supplementary note on HyDrop v2 technical improvements.

3. Fig. 2d. Some cell-type-specific ACRs do not show high overlap between platforms. Please comment on potential causes.

In the previous version, a few cell types (e.g., limbic system) were low or not present in all datasets. This can be attributed to differences in tissue dissection, as the HyDrop v2 material was dissected in-house, and 10xv2 was partly based on public data sets. To account for that, we perform subsampling of the same cell types to only include mouse motor cortex (M1) cell types. For all the analyses shown in the paper, this new subset was used.

4. Line 235. The decision to forgo batch correction needs justification. Given the number of batches and platforms compared, batch effects could confound the interpretation.
Batch correction is only done based on the technique / wet lab protocol, but not the batch itself. Earlier work supports that large wet lab differences are best corrected for using the platform as a variable when applying harmony (De Rop et al., 2023).

5. Line 265. This paragraph lacks a visual aid reference. A figure or table should be cited to support the results presented.
To clearly show the used data and the DL models that were trained, we incorporated Figures 2b and 4b for mouse and fly, respectively (See Question 6 in the previous section)

6. Fig. S5a. For clarity, split the comparison plots between HyDrop v2 and 10x into separate panels, as overlapping density plots are hard to interpret.
We re-edited the supplementary figures and focused on the integration across batches, as shown in the mouse data (Fig. S1)

7. Lines 313, 411. Typo noted: "Figure 43" and "Figure S73" should be corrected.
Thanks, this has been corrected.

8. Lines S6a–c. The color bar scales for correlation values should be standardized across comparisons to enable direct visual interpretation.
As we redid the model analysis, we now included more meaningful model comparison parameters (Figs. 2, S3, S5)

Reviewer #2 (Remarks to the Author):

In this manuscript, Dickmänken et al. developed HyDrop v2 ATAC, which is an improved version of the HyDrop v1 published previously from the same labs. The authors showed that HyDrop v2 had improved bead barcode accuracy. The data generated by v2 is better than v1 and is comparable to the commercial 10x genomics platform. They showcased the application of HyDrop v2 ATAC by generating high-quality chromatin accessibility atlases of mouse and fly. Then they showed that the data can be used to train S2F models for biological discoveries. HyDrop v2 ATAC seems to be a useful method and is good at generating high quality scATAC-seq data with much reduced cost, which is valuable in the current age genomic studies.

The current manuscript is a little bit confusing. When I first read the title and the abstract, I thought this manuscript is about HyDrop v2, the method. However, the main text spent too much effort on using the data to train S2F deep learning models and compared to models trained by the 10x data. The manuscript should either focus on the description of the improvements over v1 or the new findings in by HyDrop v2 data. My comments are mainly from the viewpoint of a potential user. In this case, the data quality and the easy-to-use/implement features are more important, and they are currently not sufficiently discussed in the paper. Specific comments are listed below which I hope to improve the manuscript:

We thank Reviewer 2 for the clear comments and suggestions. Based on the integrated feedback, we decided to refocus the message of the paper on benchmarking scATAC platforms, including our improved HyDrop-ATAC technique, with regard to S2F modeling. In other words, when the goal of a single-cell or spatial technology is to generate (a lot) of training data to train ML models, what is the best strategy, and can cheaper, custom techniques yield equivalent models, leading to equivalent biological insight, compared to commercial (and more expensive) methods? So far, only basic quality metrics have been assessed in benchmarking papers of scATAC platforms (De Rop et al., 2023), while papers evaluating DL tools only include data from one platform and/or model organism instead of different species (e.g., Kempynck et al. (2025), Johansen et al. (2024), Hu et al. (2025)). We have rewritten large parts of the paper, and we hope that the reviewer finds the new version less confusing.

1. The authors described the improvement of bead barcode generation compared to v1, which was good. The authors also claimed v2 was more sensitive than v1. Then what makes v2 more sensitive? Were there any other improvements on the ATAC chemistry? Was the improved sensitivity purely due to the improved bead barcode generation procedures? This should be clearly described.

Several factors can improve the sensitivity in capturing ATAC fragments by the barcoded sequences in a bead, including bead stability, droplet stability, purity of the barcodes within a single bead (otherwise, the contaminating barcodes take away fragments, which will get assigned to the wrong cell, or to ‘empty’ cells), the efficiency of hybridizing the ATAC fragment to the barcoded sequence within the droplet, and the efficiency of amplification of the captured fragments. The purity of the barcodes within a bead was significantly improved in the v2 barcoding strategy and was validated by single-bead Sanger sequencing. In addition, we also adapted the buffers slightly, which might have improved the nuclear lysis and the oligo number released from the bead, both within in droplet; factors that also contribute to efficient ATAC fragment to barcode pairing.
We improved the description of the different modifications and their expected impact in the Supplementary note. We also describe the limitations, such as the sequencing efficiency which is higher for HyDrop v2 compared to HyDrop v1, but still lower than 10x in the Discussion.

2. For the comparisons to v1, 10x and sci-ATAC-seq shown in Figures 1f, 4c and S1b, the metrics were heavily dependent on cell types, sequencing depth and preprocessing pipelines. Are those things the same across different methods? For example, simply downloading the count matrices from the 10x genomics website and compare to the v2 data is not sufficient.

We agree that this is important. We actually did not download the count matrices from the 10x website. We downloaded the raw FASTQ files, and processed all data in parallel, both newly generated FASTQ files downloaded FASTQ files with exactly the same preprocessing pipeline.
Regarding the sequencing depth, we effectively controlled for this by downsampled the fragment files in both fly and mouse to the same sequencing depth across platforms, 12kRPC and 36kRPC, respectively. This has been adapted and clarified in the manuscript.

3. While common metrics like unique nuclear fragments and FRiP are good to assess the method sensitivity, they are only able to show cells that survive the droplet system. At a similar cell number, is there any distortion of cell type/subtype proportions of v2 compared to other methods? For example, by downsampling to similar numbers of cells, are there any cell clusters only present in one platform, not the other(s)?

In the mouse, the cell type selection can be heavily influenced by tissue dissection (for example, of a small piece of the striatum or hippocampus is included in the cortex dissection, the cell types from these contaminating regions can be either present or absent). To address this issue, we focus purely on the cell types from the M1 cortex that are present in both atlases. As there is less 10x-based (v1 and v2) data available compared to the data we generated with the HyDrop v2 platform, we downsampled the HyDrop v2 data to the same number of cells per cell type before training S2F models to also rule out the influence of the number of cells. The tables show the cell counts from both platforms as well as the limiting cell count at full read depth. This cell count at 36kRPC as well as the read count at full depth are shown in the manuscript (Table S1 and S2). We trained several models with the same parameters on the whole cell count (at 36kRPC and full read depth) as well as on the downsampled amount of cells (at 36kRPC and full read depth).

**annotation**	**10x_count**	**HyDrop_v2_count**	**Dataset with less cells**
Astro	2095	3807	10x
Endo_VLMC	424	484	10x
L2_3_IT	3189	9586	10x
L5_6_NP	280	1156	10x
L5_ET	523	2131	10x
L5_IT	2814	8631	10x
L6_CT	1643	5780	10x
L6_IT	1563	5453	10x
L6b	128	1041	10x
Lamp5	279	1121	10x
Micro_PVM	1030	4229	10x
OPC	911	2929	10x

Oligo	3287	14550	10x
Pvalb	580	3074	10x
Sncg_Vip	368	1326	10x
Sst	364	1782	10x

In fly, no dissection is performed, and the whole animal is sequenced. All cell types are found by both techniques to a similar extent (here, the HyDrop v2 dataset includes overall only 27% more cells). The table below shows the cell count as well as the differences per cell type. We included the tables as S3 in the manuscript. We see a vast increase in neuronal cells in the HyDrop v2 dataset as they are the largest cell group in the last stage embryo (Wang et al., 2025), leading to an overrepresentation when more cells are sampled.

**annotation**	**10x_count**	**HyDrop_v2_count**	**Dataset with more cells**	**Amount of additional cells in %**
**Epidermis**	40843	36833	10x_v2	10.89
**Fat_body**	17681	19985	HyDrop_v2	13.03
**Glia**	5372	5022	10x_v2	6.97
**Head_Ectoderm**	3117	4059	HyDrop_v2	30.22
**Hemocytes**	3808	3617	10x_v2	5.28
**Hindgut**	5582	6682	HyDrop_v2	19.71
**Malpighian_tubule**	3776	4904	HyDrop_v2	29.87
**Midgut**	32156	37442	HyDrop_v2	16.44
**Midgut_acidification**	2209	3283	HyDrop_v2	48.62
**NA_lowQ**	51090	77174	HyDrop_v2	51.06

**Neuroblasts**	6945	11134	HyDrop_v2	60.32
**Neuronal**	31561	61027	HyDrop_v2	93.36
**PNS_sens_neurons**	3876	7895	HyDrop_v2	103.69
**Pharnyx**	11747	10697	10x_v2	9.82
**Primordium_all**	6627	7770	HyDrop_v2	17.25
**Salivary_gland**	3853	5373	HyDrop_v2	39.45
**Somatic_muscles**	14431	15269	HyDrop_v2	5.81
**Tracheal_system**	6873	7504	HyDrop_v2	9.18
**Visceral_muscles**	5495	5803	HyDrop_v2	5.61
**Yolk**	3396	4063	HyDrop_v2	19.64
**muscle_attachement_Strip e**	6288	5068	10x_v2	24.07

Also note that the results of our motif enrichment correlated well between techniques, supporting the comparability of cell type recovery as seen in Figure S2h-i.

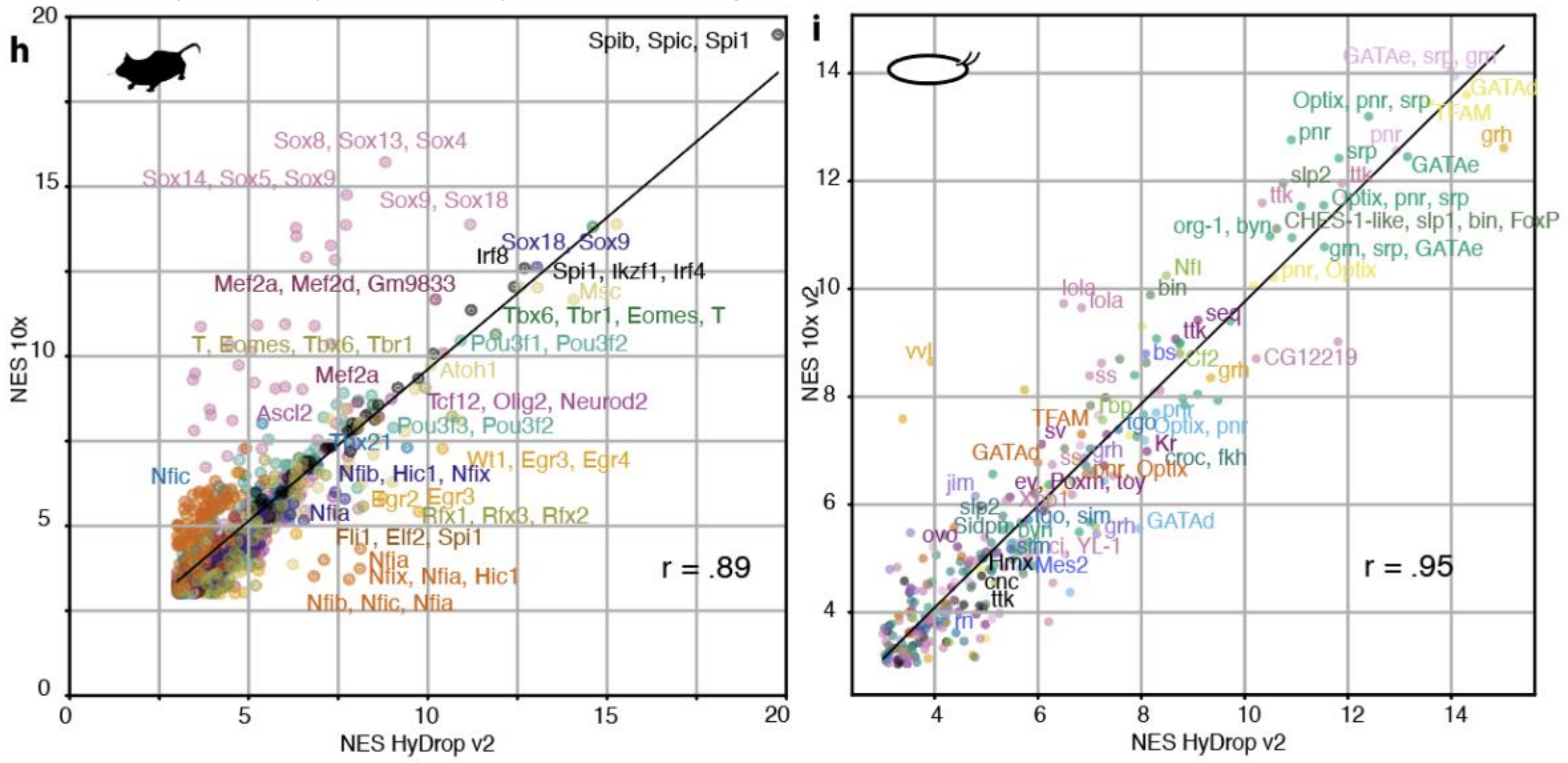

Figure S2h-i

4. From a user point of view, what exactly is the niche of HyDrop v2 ATAC and when should I use it? The lower cost is probably one factor to consider, but this is only true for labs who can build or already have the droplet device. In addition, sample multiplexing like txci-ATAC-seq and scifi-ATAC-seq would also reduce cost, which seems to be an easier and more straightforward way of lowering the cost. For a broader audience, please provide some general guidance on choosing HyDrop v2 and the commercial 10x genomics.

As shown in the HyDrop v1 paper (De Rop et al. 2022), the microfluidic setup is relatively straightforward, also when compared to other platforms mentioned in De Rop et al. (2023). However, we understand that it might require more effort to set it up and produce the beads. The droplet generation device costs around 20-30k (ONYX, available from Atrandi Biosciences), while the Chromium is more expensive. Thus, for large atlassing efforts (including within companies), this can be worth it. We provide detailed protocols that make the generation of the beads and microfluidics chips feasible.
Upscaling with a Chromium device and barcoded Tn5 as seen in OAK seq (Wu et al., 2024) might be an alternative, but this has not yet been tested on a broader level. Here, OAK is also estimated to be compatible with the HyDrop setup as mentioned by Wu et al. (2024). Furthermore, techniques like scifi-ATAC-seq combine Tn5 barcoding with 10x Genomics, and are therefore also applicable to HyDrop-ATAC (first index nuclei with different Tn5 barcodes, then pool and use for the droplet microfluidics part the HyDrop-ATAC protocol instead of the 10x Genomics kit and Chromium). This is actually a very interesting scenario that future studies could investigate to even further upscale the data generation and even further reduce costs. In our view, this does not 'compete' with HyDrop-ATAC itself, but is complementary to it, and could actually synergize with HyDrop-ATAC.

When compared to pure plate-base assays, that do not use droplet microfluidics, such as sciATAC-seq, we find that HyDrop-ATAC has higher sensitivity and quality. In addition, plate-based combinatorial indexing (Wu et al., 2024) is not necessarily cheaper, as there are indirect costs of automating the platforms and the laborious nature of the workflow which can add up to the costs of the assay. In addition the sciATAC-seq workflow requires access to barcoded Tn5 which can be quite expensive with commercial options. The in-house synthesis option of the Tn5 would require deep technical know-how of protein synthesis and establishment of extensive synthesis workflow. In contrast, a trained technician can process >100k cells within 1.5 days using the HyDrop v2 setup. Important for application to stored patient samples, the current data generation was based on snap-frozen tissue, achieving high-quality output data as shown in this paper.

To include plate-based data in the benchmarking, we added in-depth comparisons of the sciATAC fly embryo data. We are aware that sciATAC is not ultra-high throughput, but so far, no comparable public datasets of fly or mouse generated with scifi-atac, txci-atac-seq, or similar are available. Importantly, sciATAC-seq profiles show a much wider fragment coverage that is clearly visible across individual loci (shown for somatic muscles differentially accessible region (DAR)):

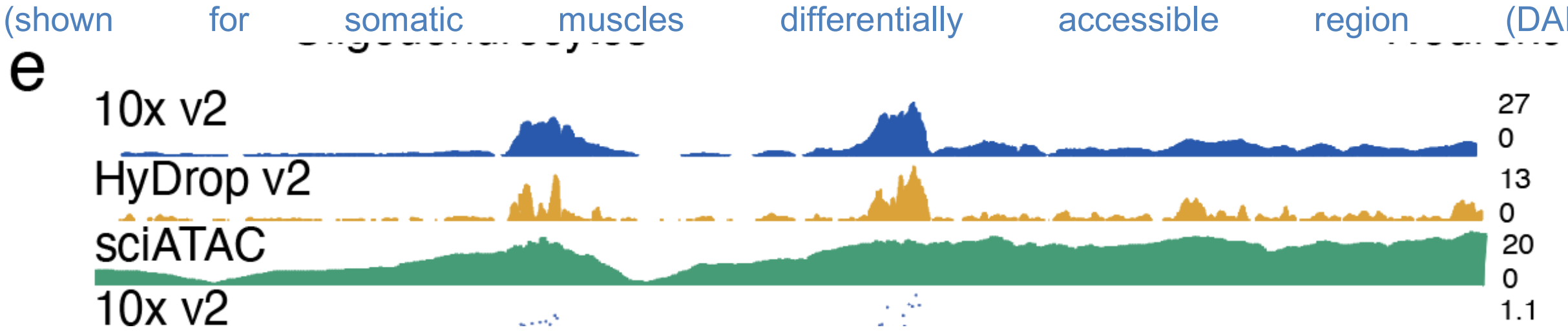

On the genome-wide level, this is also reflected in the carrot plots (Figs. 2c), and by the fragment size distribution, showing much larger fragments in sciATAC data (Fig. Sf).

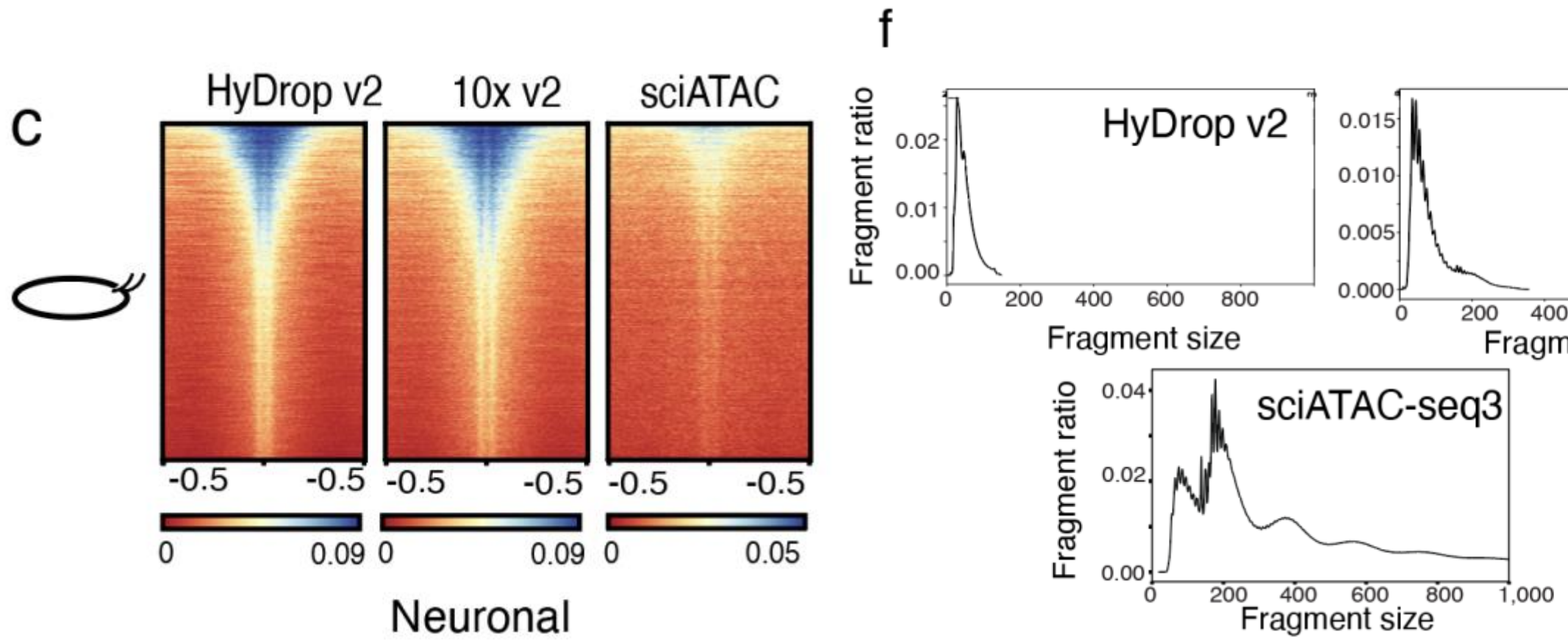

We discovered that the individual insertions of Tn5 (cutsites) of sciATAC-seq better resemble 10x and HyDrop v2-based data (to our knowledge, this has not been shown before). Cut sites for somatic muscles DAR shown below:

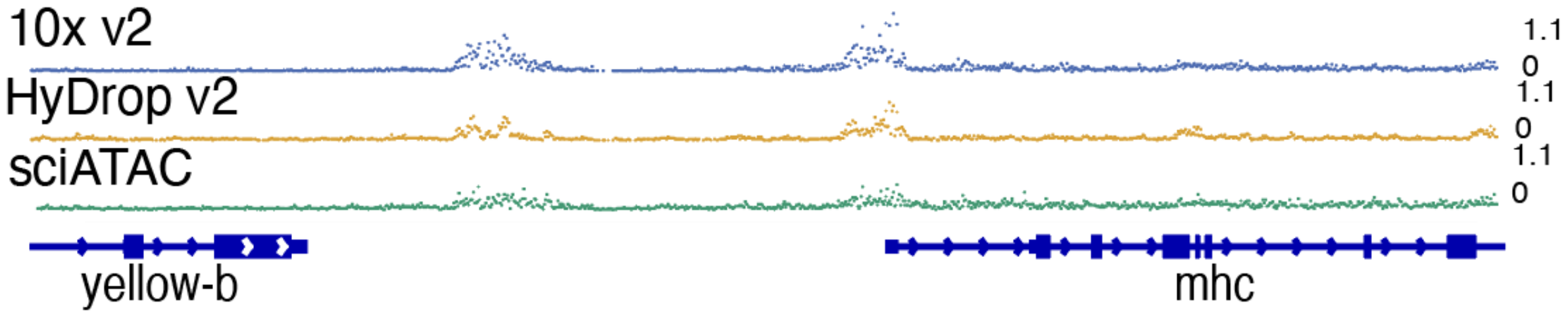

In other words, we find that the obvious quality issue in sciATAC data can be partially mitigated by analyzing the cut sites instead of the coverage information. This, however, is not yet incorporated into standard scATAC processing.

For the mouse, the differences between fragment sizes are not as outspoken (Fig. S7e).

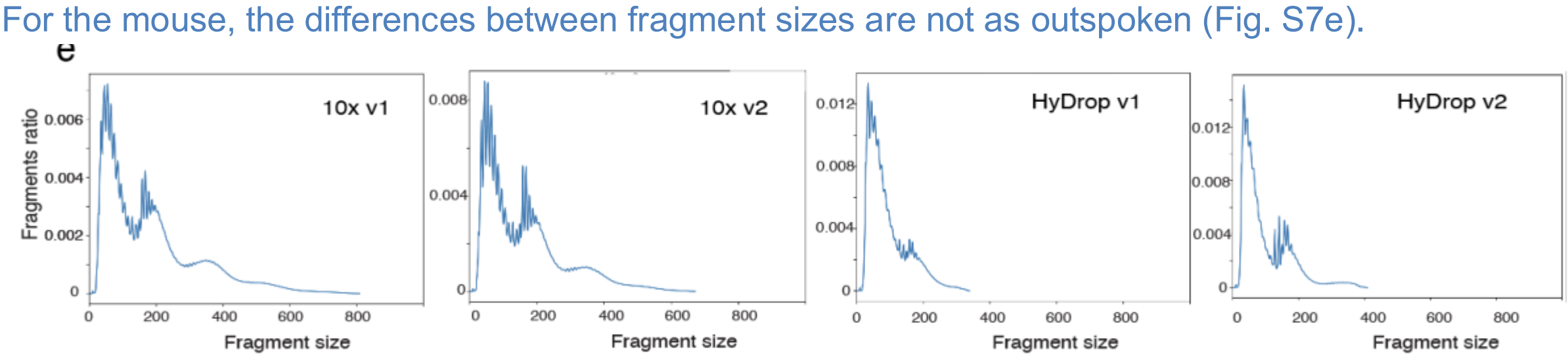

5. The authors claimed that the 110k mouse motor cortex atlas cost 1080.6 euros with v2, and would have cost 1398.29 euros with v1 and 15,330.70 with 10x. A breakdown of the cost per reagent to see where the major cost comes from would be helpful.

We added two tables of the cost breakdown to the supplementary material: the costs for the HyDrop v2 beads (Table S6) and for the HyDrop v2 reagents (Table S7). The table for the beads shows a total of 60 reactions, resulting in a cost per reaction of 27.44 euros. Together with the reagents to conduct the scATAC barcoding, sequencing 10-12k cells costs approximately 100 euro.

6. The authors showed that the Trithorax motif was successfully detected by models from HyDrop ATAC v2 and sci-ATAC, but not 10x. I'm not sure how to interpret the result. First, the authors pointed to Figure 5i to support this claim, but it is not entirely clear where I'm supposed to look at. Is it the label "Tri" that we should be focused on? Some highlight would be helpful. In the position labelled "Tri", some bases have positive scores, though fairly small compared to the Grh ones, in the HyDrop v2 and sci-ATAC tracks, and some bases have negative scores in the 10x track. Is this the evidence? The figures were blurry and I'm unable to read the small logos after zooming in. Some explanation would be nice. Second, perhaps more important, could the authors speculate and discuss why the motif is missed in the 10x model? It cannot be data quality,

since the quality of the date sets were comparable. Was it due to cell numbers or something else? This is where some knowledge can be learned.

We agree that there is some variability in nucleotide importance score; thus, the motifs discovered per model in the *synthetic* enhancer sequence. Small differences for individual loci are usually attributable to model variability or but could also be caused by the design of the enhancer sequence, as it is not a genomic one to which the Reviewer is referring. The synthetic sequences were not designed by us but by De Almeida et al (2023). An effect of the cell count for that specific class is unlikely, as there are only 5.81% more cells present. As the Hydrop v2 model, however, in the fly contains overall 27% more cells, this might underlie a better learning effect for somatic muscle cells in contrast to other cell types. However, the model parameters of both models are on par. For clarity, we added highlights to the motifs in the synthetic sequence.

When looking at the genomic sequences, as in F4i, we see the same motifs appearing in the individual locus (Fig. 4i), the ttk motif is found by both models. Additionally, the overall motif importances (identified by *tfmodisco lite*) are similar to the same between the models.

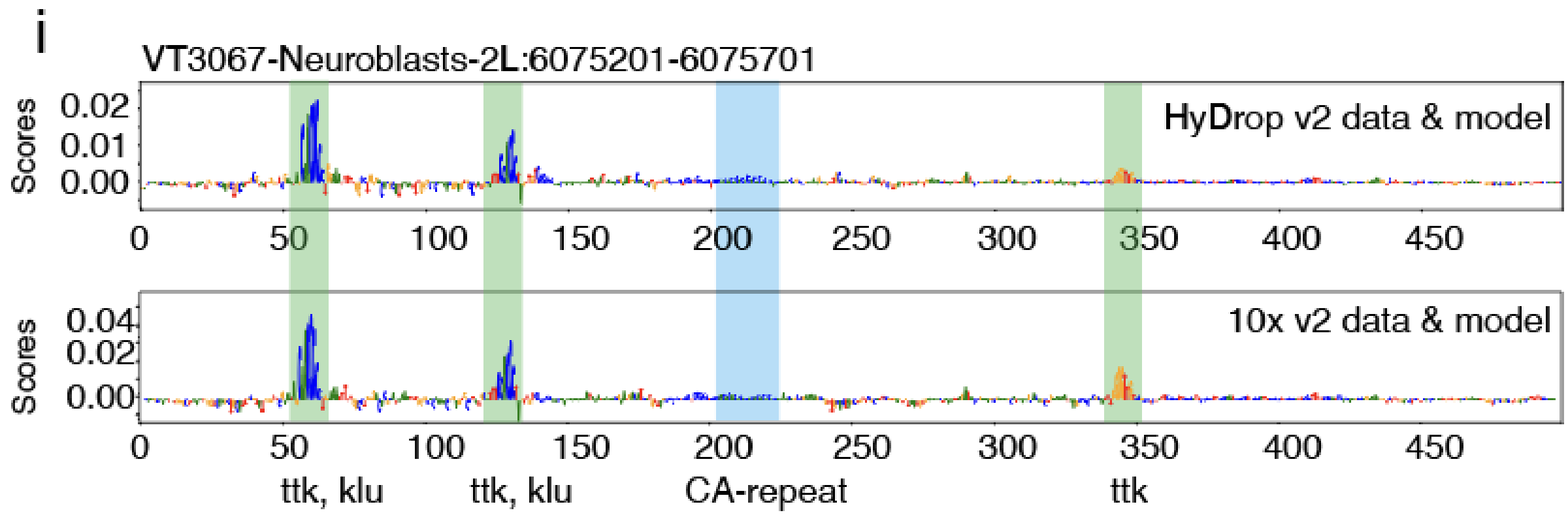

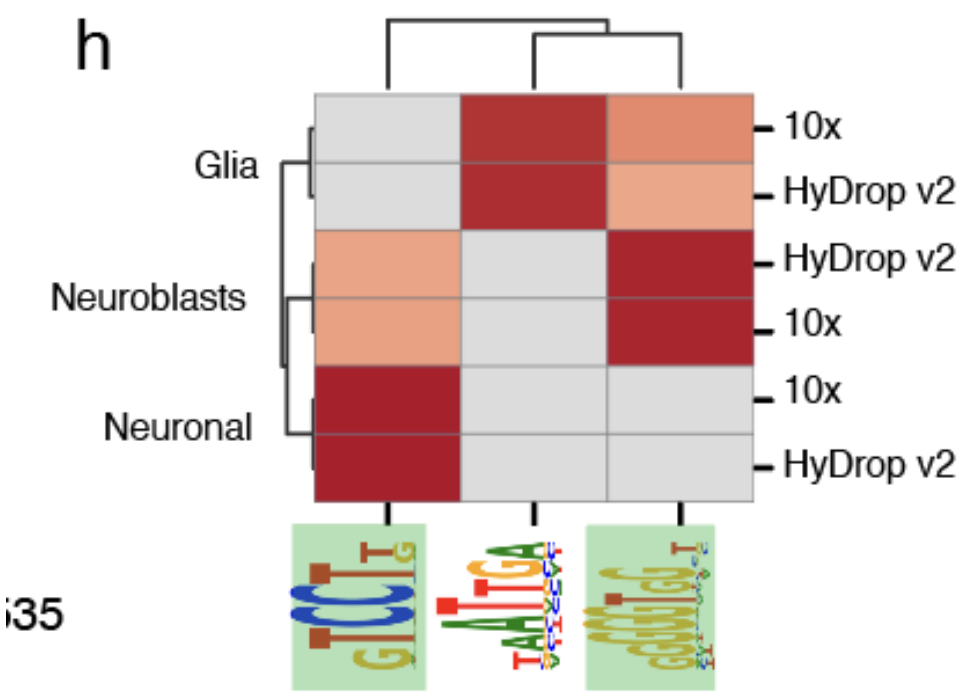

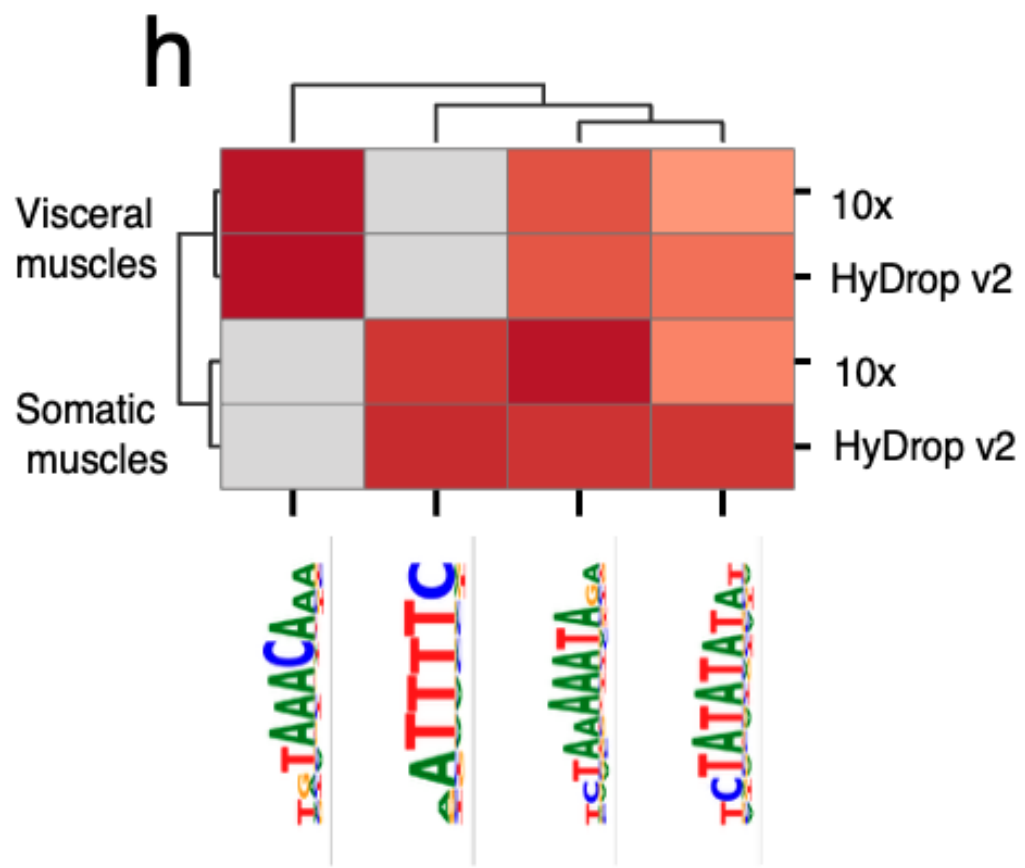

7. The authors also claimed the droplet generated in-house is better than Atrandi, and points to Figure S1d,e. However, Figure 1d is a table with numbers. Neither the main text nor the figure legend explained what the expected numbers would be, and why those numbers tell us that the in-house one is better than the Atrandi one.

We first wanted to switch to the Atrandi chips to generalize the bead production. However, when using the Atrandi chip to generate the beads, the flow was not stable in our hands. This resulted in a variable bead size and, more importantly, deformed beads. The deformed and 'sticky' beads were even more pronounced after freezing (Fig. S1e). In S1e, some beads appear brighter/more see-though as they are less solid which again impairs the microfludic flow during the experiment. As a result of the impaired flow rate, solidity issues, and freezing artifacts, we changed the bead production to the in-house bead generator. We added this clarification to the figure description and the supplementary text.

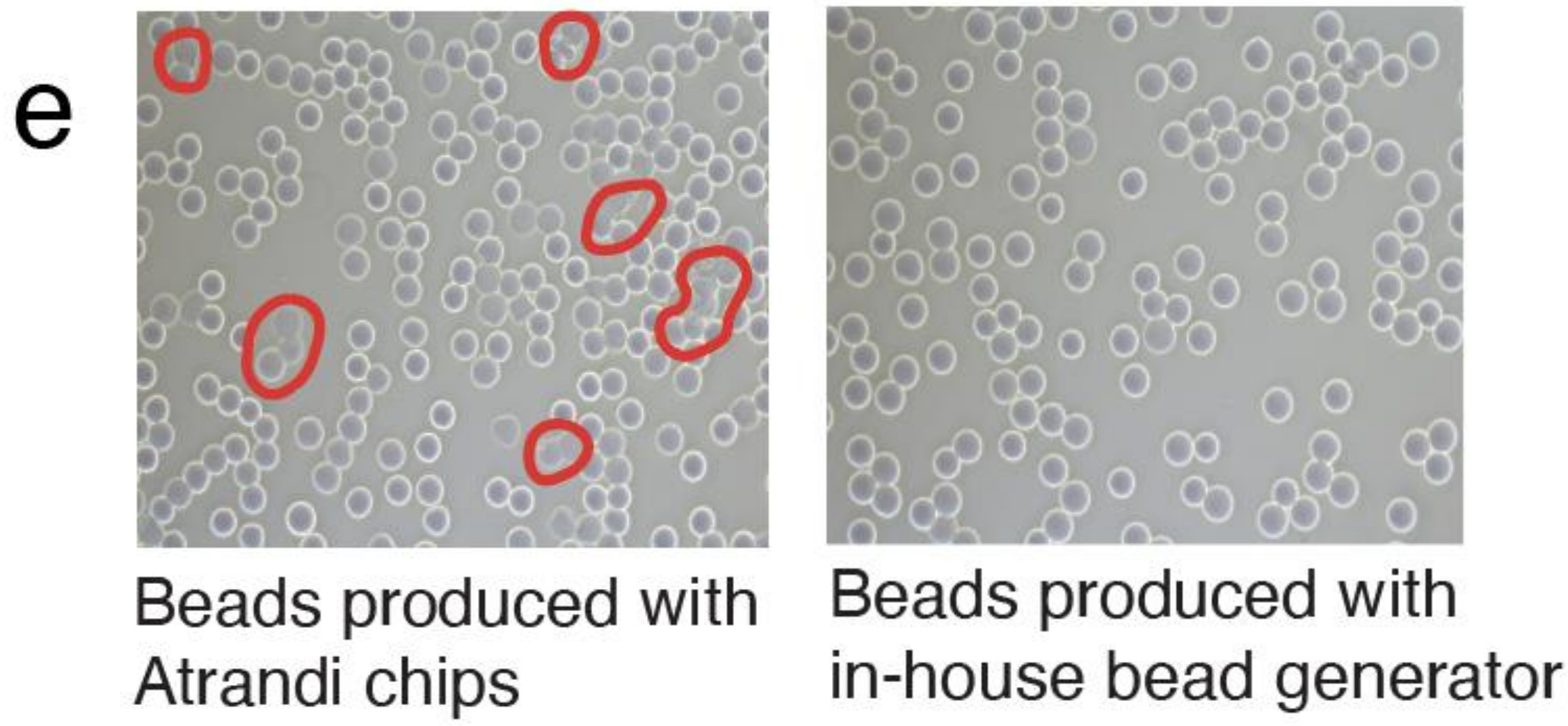

8. The writing of the manuscript felt rushed. There are many texts with unclear logic, some poor choices of reference and some typos, all of which make the reading not very pleasant:

8.1) Line 46: the authors use Ref1 (Buenrostro2015) to support the claim that cell type identify is encoded in CREs, which is a lazy choice. It was the paper that invented scATAC-seq, but it is not the first to demonstrate that cell identity is encoded in enhancers. I'm sure there are better choices to make this point.
We agree and added the book The Regulatory Genome: Gene Regulatory Networks In Development And Evolution. (Elsevier, 2010). By Davidson, E. H., to the reference list.

8.2) Lines 54 and 56: when the authors introduce scATAC-seq, only Buenrostro2015 was cited, but people generally think it was Buenrostro2015 and Cusanovich2015 (doi: 10.1126/science.aab1601) together invented scATAC-seq, and they should be cited together.
We agree and have adapted the citations.

8.3) The authors claimed to have 7 batches of beads for the v2 benchmarking shown in Figures 1f, S1b, S1d. They sometimes were labelled A,B,C,D,E,F,I and sometimes bathes 1,2,3,4,5,6,7. I assume they are the same? Better make them consistent.
This has been adapted in Figure S1.

8.4) Many figures and figure panels were poorly organised. For example, in line 127, after Figure 1a was mentioned, Figure 1d was followed. Figure 1b is not referenced at all in the main text, and Figure 1c is referenced after Figure 1d. Similarly, in line 171, Figure 1e was followed by Figure 1h, and Figures 1f and g were only referenced later. In line 194, Figure 1e was followed by Figure 4a. In line 313, Figure 43 was mentioned. I did not list all of them, please check and reorganise the figures, so that the readers will not jump back and forth.
We carefully reorganised the text and adapted the order of the main and supplementary figures accordingly.

8.5) Both sciATAC and sci-ATAC were used. Please be consistent. We adapted this in the manuscript.

8.6) In Figure S1g, there is no data after 700bp for 10x v2, no data after 400 bp for HyDrop. Are those correct?
Yes, these are normal fragment size plots generated by pyCistopic.

8.7) Marker gene names should be in italic format. For example, genes mentioned in lins 531, 532 and 1157. I did not list all of them.

We adapted this in the manuscript and added abbreviations for the transcription factors to the Figure text in Figure 4 for additional clarity.

Reviewer #3 (Remarks to the Author):

In Dickmänken et al, the authors benchmark an improvement on their previous technology (Hydrop v1, De Rop 2022) and use it to perform snATAC-seq and machine learning on the subsequent accessible chromatin profiles. Hydropv1 had already introduced the lower pricing, but had problems at the level of the barcode stability and complexity. This improved method, which addresses these previous limitations, is meant to be both a time-saving and cost-saving technology compared to commercial platforms (10x Genomics, primarily) and to other opens source methods such as sciATAC. As per it's name, Hydrop belongs to the microfluidic, bead-based methods for single-nuclei capture and barcoding. The authors combine improvements in their previously developed technology with previously developed machine-learning algorithms to train S2F models of cell-type specific CREs. As such, the of the major strength of this work is to 'democratize' the use of snATAC at a more reasonable cost which could open its use for more groups and to more cell types/conditions as scale can be achieved cheaply without compromising quality. The work proposes a series of elegant benchmarks by which to compare Hydropv2 vs other methods to show their equivalency, both in mouse brain and in the Drosophia embryo. The comparison of Hydropv2 to the 10x platform is well done and the use of it to train S2F models where large datasets are necessary underscores the importance of having scalable technical platforms. In the Drosophila the manuscript nicely shows that the generated data + the S2F models can recapitulate in vivo enhancer activity with their predictions, as well as being able to predict synthetic enhancer activity in specific tissues. The authors show the power of using S2F models which contain all the cell-types to predict the specificity (or not) of an enhancer. Despite all these positive points and the well-done work, the manuscript doesn't reflect a major advancement in how snATAC is either performed or analyzed on the technical level—rather the major advancement is in price/nucleus. Codes, protocol and data are nicely provided, making it a good, open-access reference source.

We thank Reviewer 3 for the positive feedback. The major advancement is that we integrate sequence-to-function models into technology benchmarking, or in other words, that we assess the power of different technologies in their capacity (and cost) to generate high-quality training data for deep learning models. To our knowledge, this is the first time that such a benchmarking study has been performed, and we believe that this represents a new way of thinking, going beyond per-cell assessments of quality metrics to atlas-level and model-level assessment. Significant additional advancements and novelties include: (i) a much improved HyDrop-v2 protocol with significantly better sensitivity and scale compared to HyDrop-v1; (ii) the largest Drosophila scATAC-seq atlas of the embryo with 600,000 cells; (iii) new CREsted models for the mouse brain and the fly embryo that can be used by the community; (iv) the first comparison of TF footprints between scATAC-seq technologies.

Some aspects of the manuscript could be improved for clarity by figure and associated text:

Figure 1:
- For clarity – in Figure 1, is the 10x data from the 10x Genomic's website used (cited in the data availability section) and the data from BICC data used? Also reference to Refs #12 and 24 which have additional datasets.

Thanks for noticing that this was not clear. We improved the description of the data sets in the text. Indeed, for the mouse cortex we used the 10x datasets from the website, but starting from the raw FASTQ files. Note that in the revised version, we left out the BICCN data as the sample preparation and platform are not directly comparable, while we are aiming to reduce influencing factors as much as possible.

- 1h – I'm not sure that I agree there is no batch effect. The effect doesn't overwhelm the cell type specific features that cause clustering, but those are generally driven by a clear set of open enhancers. However, it's also clear that we don't end up with a even distribution of beads between the different clouds, and the paper doesn't analyze, to my recall, how the peak calls very per bead batch. I assume the seven batches were tested with the same pool of cells, but this was not super clear on first reading.

For batch correction, harmony was performed with the pyCisTopic tool on the variable of the platform/experimental technique. It has been shown that this is effective in correcting for technical batch effect (De Rop et al., 2023). Figure S1h shows the batch effect from the four different platforms:

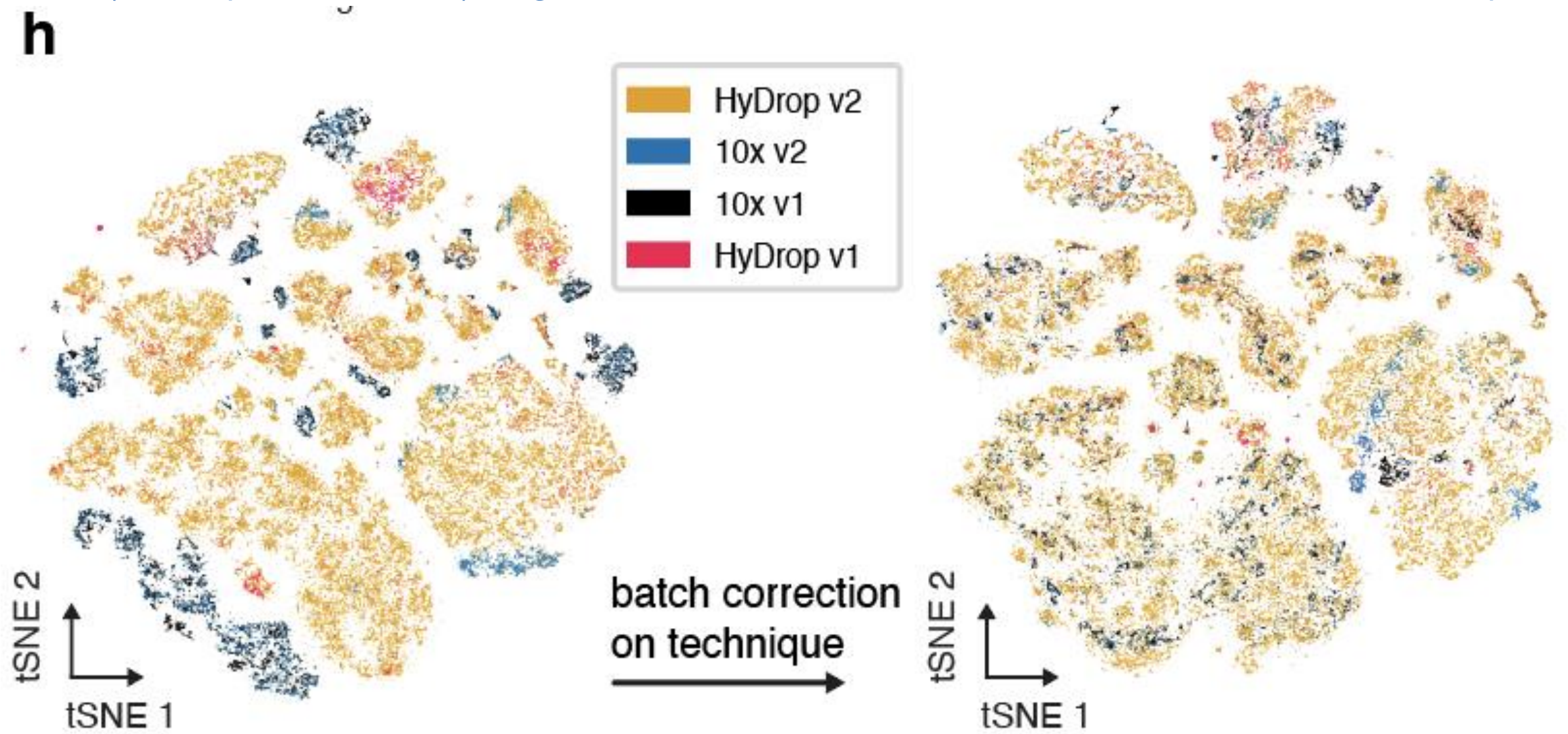

When only processing HyDrop v2 samples, no batch effect correction is needed to cluster and annotate cell types (and to generate bigWig files per cell type for S2F model training). Within each cluster, the effect of the different runs can be observed, with each cluster showing a clear presence of cells from all seven bead batches when no harmony is applied (Fig. S1g). The seven batches were tested on the same biological system (mouse cortex); however, they came from different dissections. Also, the experiments with seven batches were performed on different days, but with several replicates. Thus, we do not control for the factor of day-to-day variability, but also in a real setting, experiments are being performed over weeks.

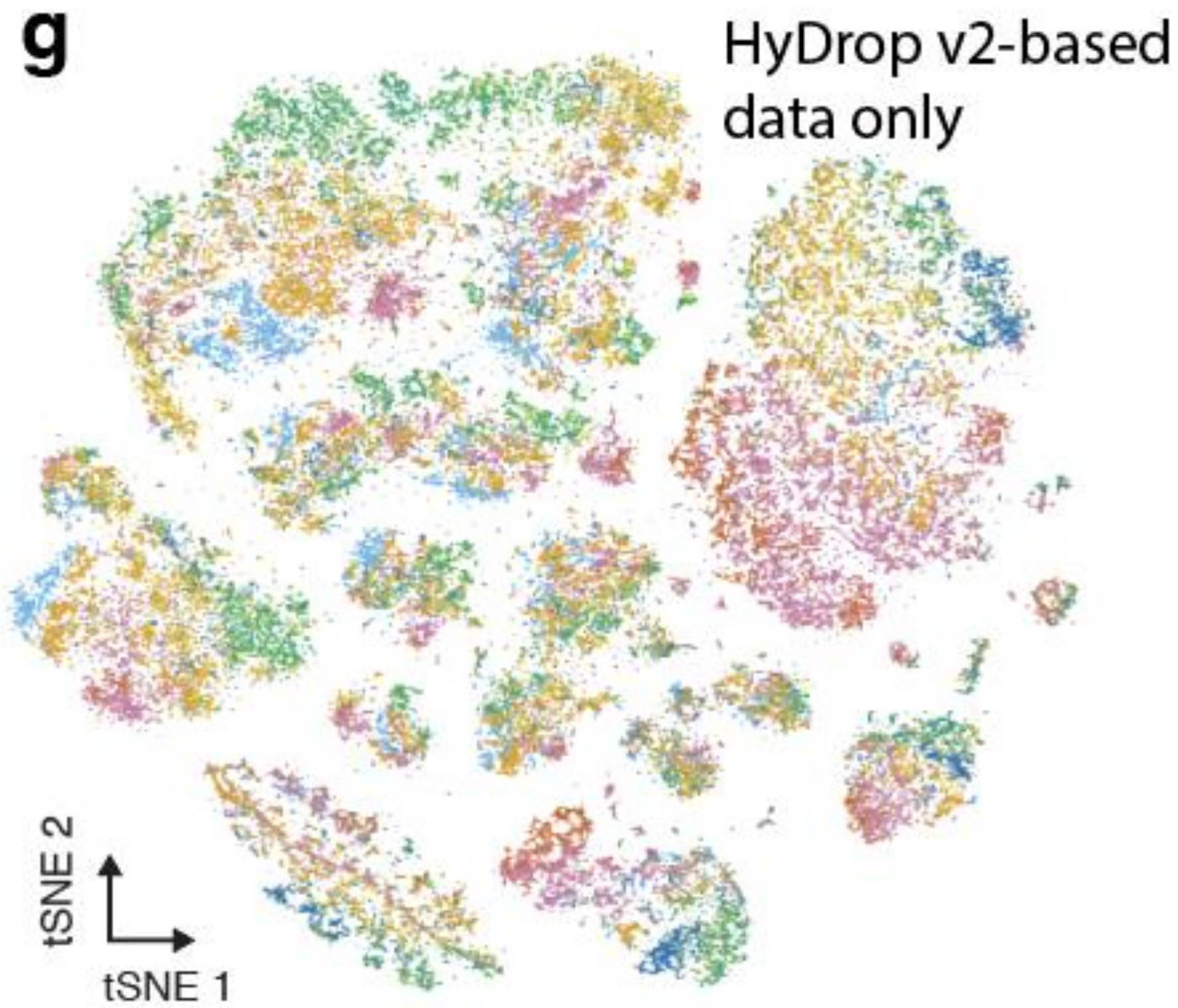

Figure 2

• For the purposes of what Fig2a is meant to show, I think it should also be shown colored by experiment type; i.e. put S2A in the main text, as that's the integration that actually makes the point, not the one colored by cell type. In S2A, we see a population that is captured only by 10x and the HydropV1. Ideas about that this is?

We agree that the integration can best be shown with an embedding colored by the different platforms used to generate the data. In the previous manuscript, the mouse dataset was generated by dissecting the mouse cortex. Here, the cell type selection can be heavily influenced by tissue dissection. To address this issue, we focus on the M1 cortex cell types that are both present in the data. With the subset dataset, the integration is clearer and not biased by certain cell types being disproportionately captured due to dissection artifacts. We still see a large cluster on the right with fewer 10x and HyDrop v1 cells. This cluster contains oligodendrocytes, which are the main cell type captured in M1. They are, thus, overrepresented when sampling more cells (10x combined: 3,263 vs HyDrop v2: 12,524 oligodendrocyte cells).

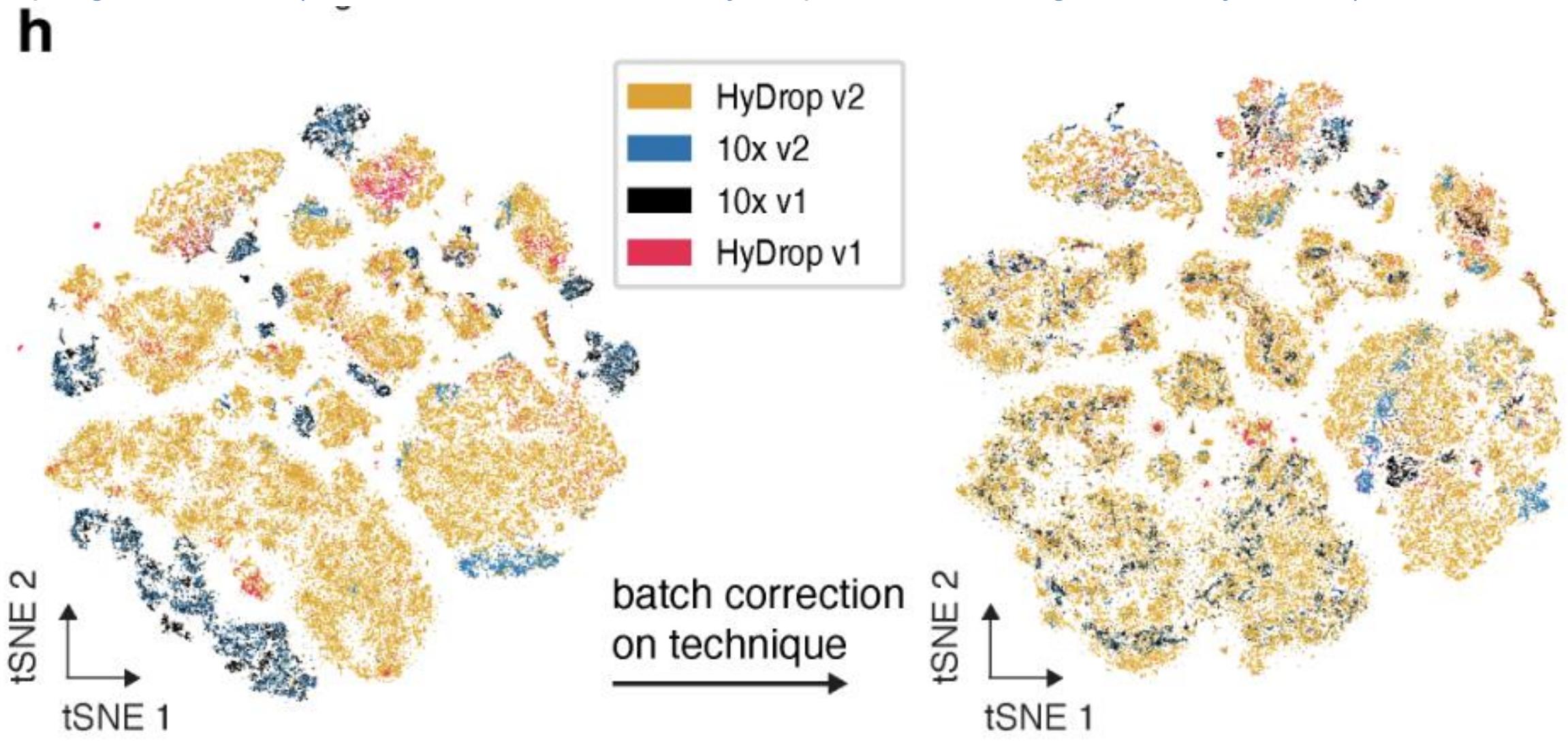

• Do you capture the same cell types – my feeling was that is was predominantly a problem of the labeling discrepancy between the 10x dataset and the Hydrov2 dataset.

As mentioned in the previous question, there is indeed a discrepancy between the number of cells present in the dataset coming from the different platforms. As in the mouse, the cell type selection in heavily influenced but the tissue dissection, so we subsampled only the cell types of M1 (motor cortex). However, as there is less 10x-based (v1 and v2) data available than we generated with the HyDrop v2 platform, we again supsampled the data to the same number of cells per cell type before training S2F models to also rule out the influence of the number of cells. In the table below, the cell count used for the full model (all cell types, at full sequencing depth) is shown. We added both tables with cell count at full sequencing depth and downsampled to 36kRPC to the manuscript.

annotation	10x_count	HyDrop_v2_count	Dataset with fewer cells
Astro	2095	3807	10x
Endo_VLMC	424	484	10x
L2_3_IT	3189	9586	10x

L5_6_NP	280	1156	10x
L5_ET	523	2131	10x
L5_IT	2814	8631	10x
L6_CT	1643	5780	10x
L6_IT	1563	5453	10x
L6b	128	1041	10x
Lamp5	279	1121	10x
Micro_PVM	1030	4229	10x
OPC	911	2929	10x
Oligo	3287	14550	10x
Pvalb	580	3074	10x
Sncg_Vip	368	1326	10x
Sst	364	1782	10x

In fly, no dissection is performed, and the whole animal is sequenced. All cell types are found by both techniques to a similar extent (here, the HyDrop v2 dataset includes overall only 27% more cells). The table below shows the cell count as well as the differences per cell type. We included the tables as S3 in the manuscript. We see a vast increase in neuronal cells in the HyDrop v2 dataset as they are the largest cell group in the last stage embryo (Wang et al., 2025), leading to an overrepresentation when more cells are sampled.

annotation	10x_count	HyDrop_v2_count	Dataset with more cells	Amount of additional cells in %
**Epidermis**	40843	36833	10x_v2	10.89
**Fat_body**	17681	19985	HyDrop_v2	13.03
**Glia**	5372	5022	10x_v2	6.97
**Head_Ectoderm**	3117	4059	HyDrop_v2	30.22

**Hemocytes**	3808	3617	10x_v2	5.28
**Hindgut**	5582	6682	HyDrop_v2	19.71
**Malpighian_tubule**	3776	4904	HyDrop_v2	29.87
**Midgut**	32156	37442	HyDrop_v2	16.44
**Midgut_acidification**	2209	3283	HyDrop_v2	48.62
**NA_lowQ**	51090	77174	HyDrop_v2	51.06
**Neuroblasts**	6945	11134	HyDrop_v2	60.32
**Neuronal**	31561	61027	HyDrop_v2	93.36
**PNS_sens_neurons**	3876	7895	HyDrop_v2	103.69
**Pharnyx**	11747	10697	10x_v2	9.82
**Primordium_all**	6627	7770	HyDrop_v2	17.25
**Salivary_gland**	3853	5373	HyDrop_v2	39.45
**Somatic_muscles**	14431	15269	HyDrop_v2	5.81
**Tracheal_system**	6873	7504	HyDrop_v2	9.18
**Visceral_muscles**	5495	5803	HyDrop_v2	5.61
**Yolk**	3396	4063	HyDrop_v2	19.64
**muscle_attachement_Stripe**	6288	5068	10x_v2	24.07

Also note that the results of our motif enrichment correlated well between techniques, supporting the comparability of cell type recovery as seen in Figure S2h-i.

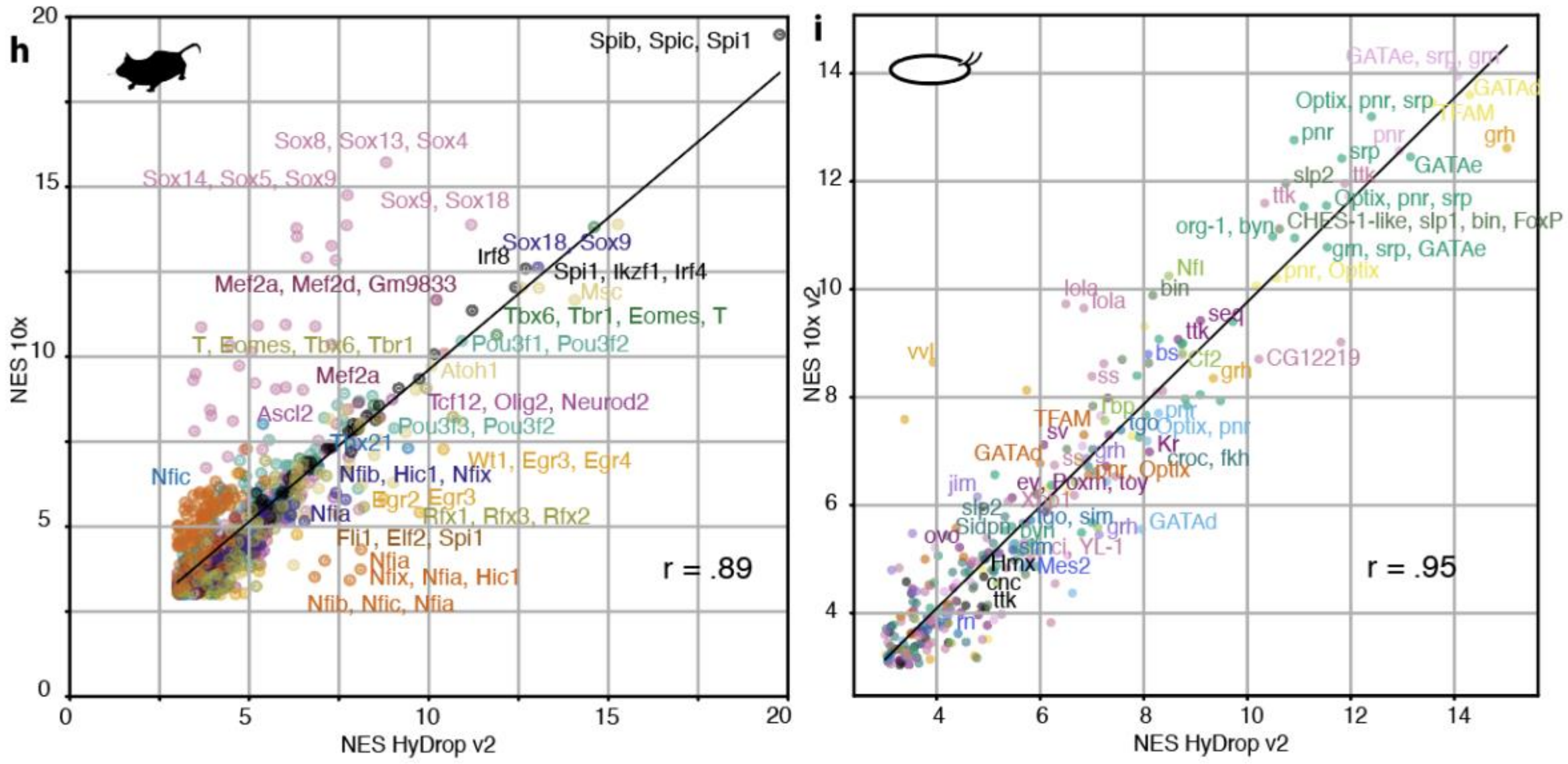

Figure S2h-i

• I find it not intuitive that the scaling on the x-axis is not the same for the chosen DAR examples. Also, would It be possible to add called peaks from macs as an additional bar above each sample. This would give us a better idea if the data is generating similar MACs peak calls and thus would yield similar downstream results.

Here, the problem is that if I take the same scale (especially in fly), a few peaks will not be visible at single locus scale, which might especially be problematic for sciATAC data. Here, as the fragment size deviates widely from that of 10x and HyDrop v2 data (see below). For the whole genome coverage, we show the carrot plot heatmap that gives a better overview on comparability.

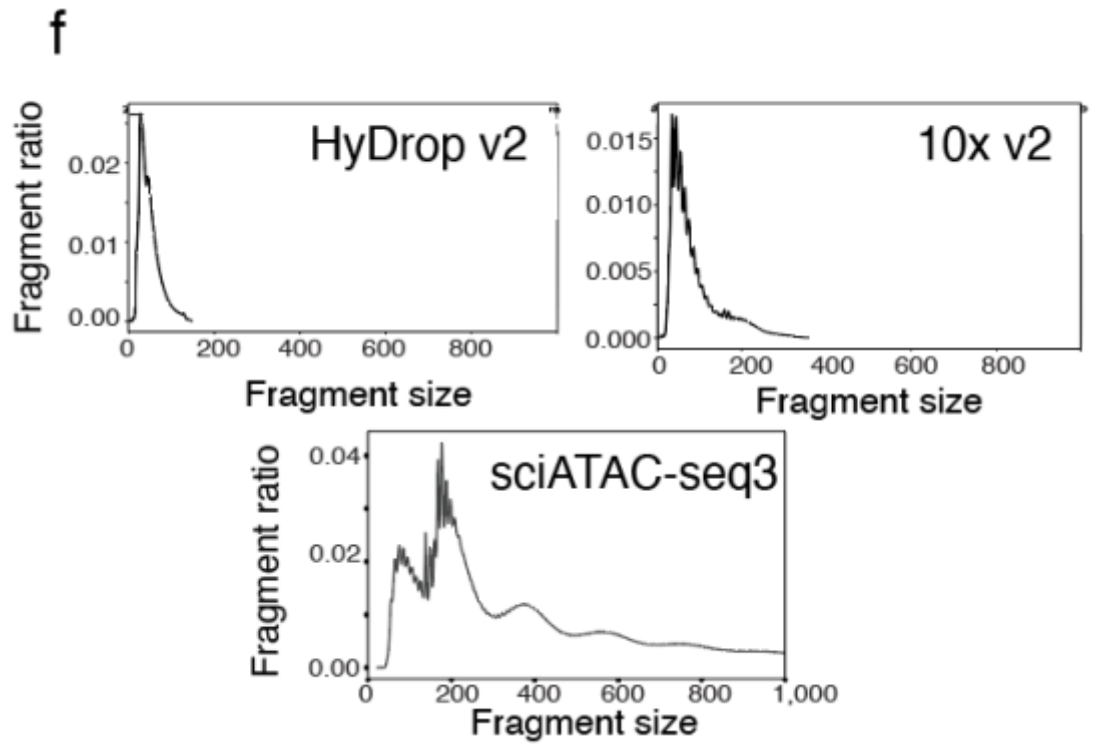

Therefore, we partly opt for a scale-adapted visualisation of the fragment coverage (top) in individual loci, and not scale-adapted for the cutsite quantification (bottom).

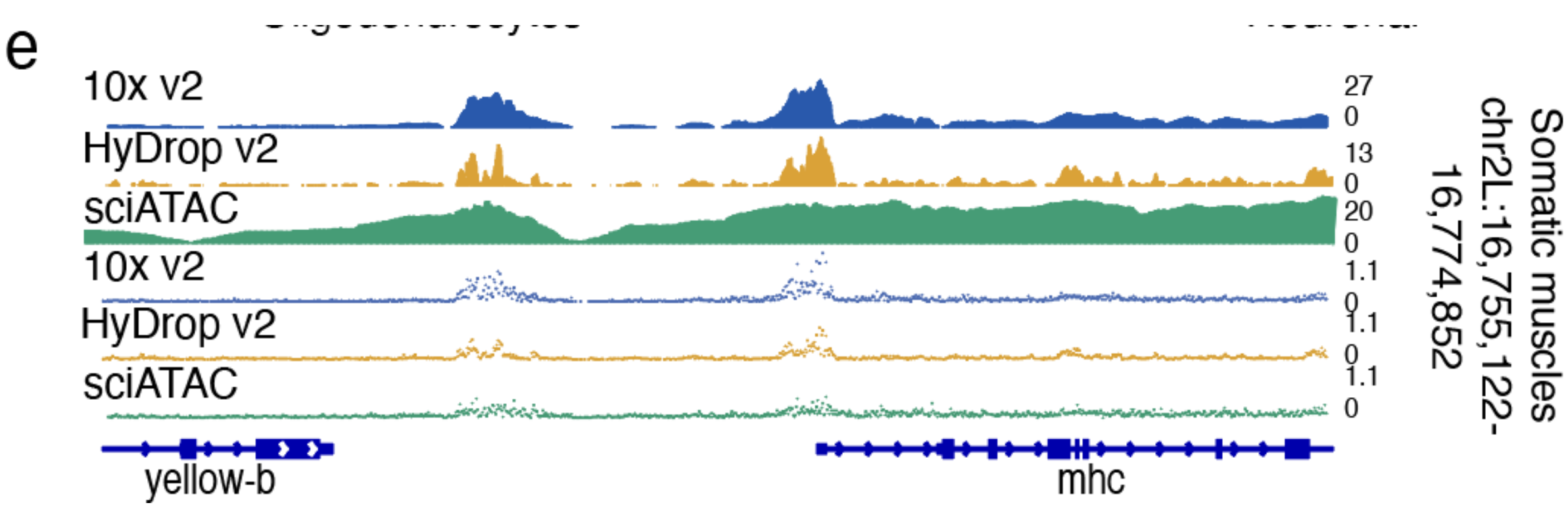

At the whole genome level, the coverage can be seen in the carrot plots of S2g for fly.

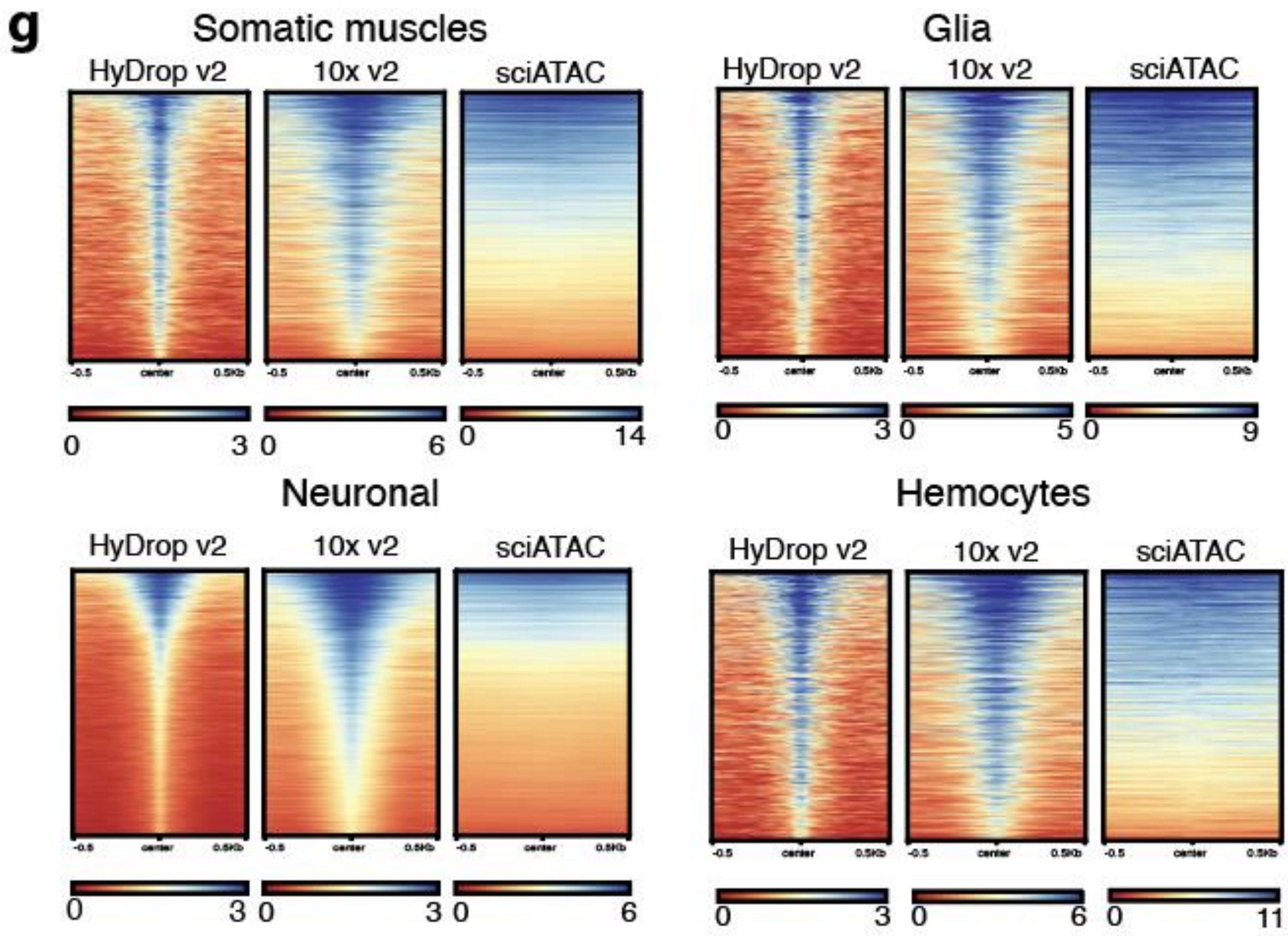

Again, in contrast to the mouse, we opt for an adapted scale as otherwise the specificity of sciATAC fragments would not be visible.

In mouse, the fragment size is more comparable between platforms:

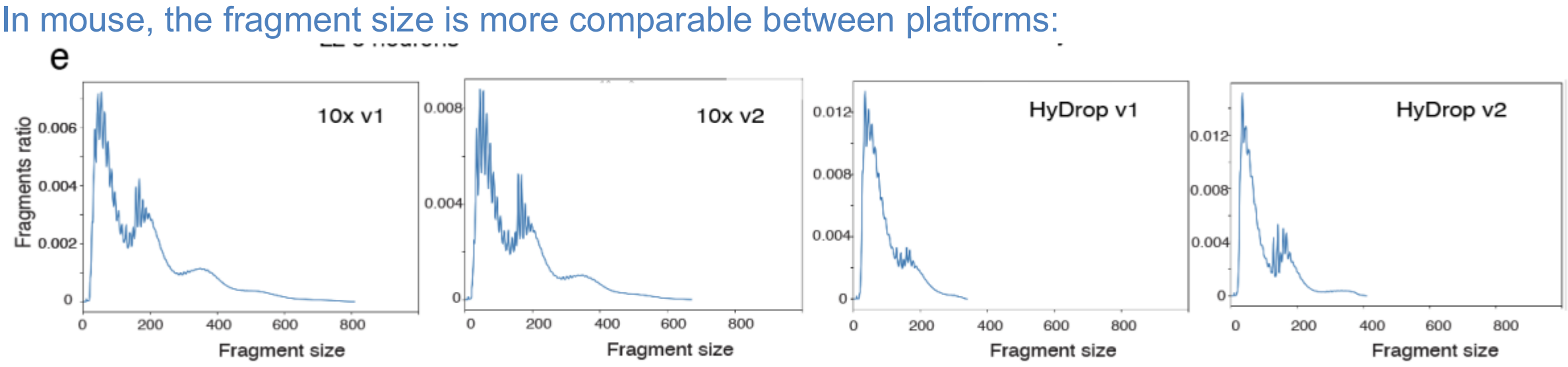

Thus, we chose the same scale to show the accessibility of cell type-specific DARs on genome genome-wide level:

e

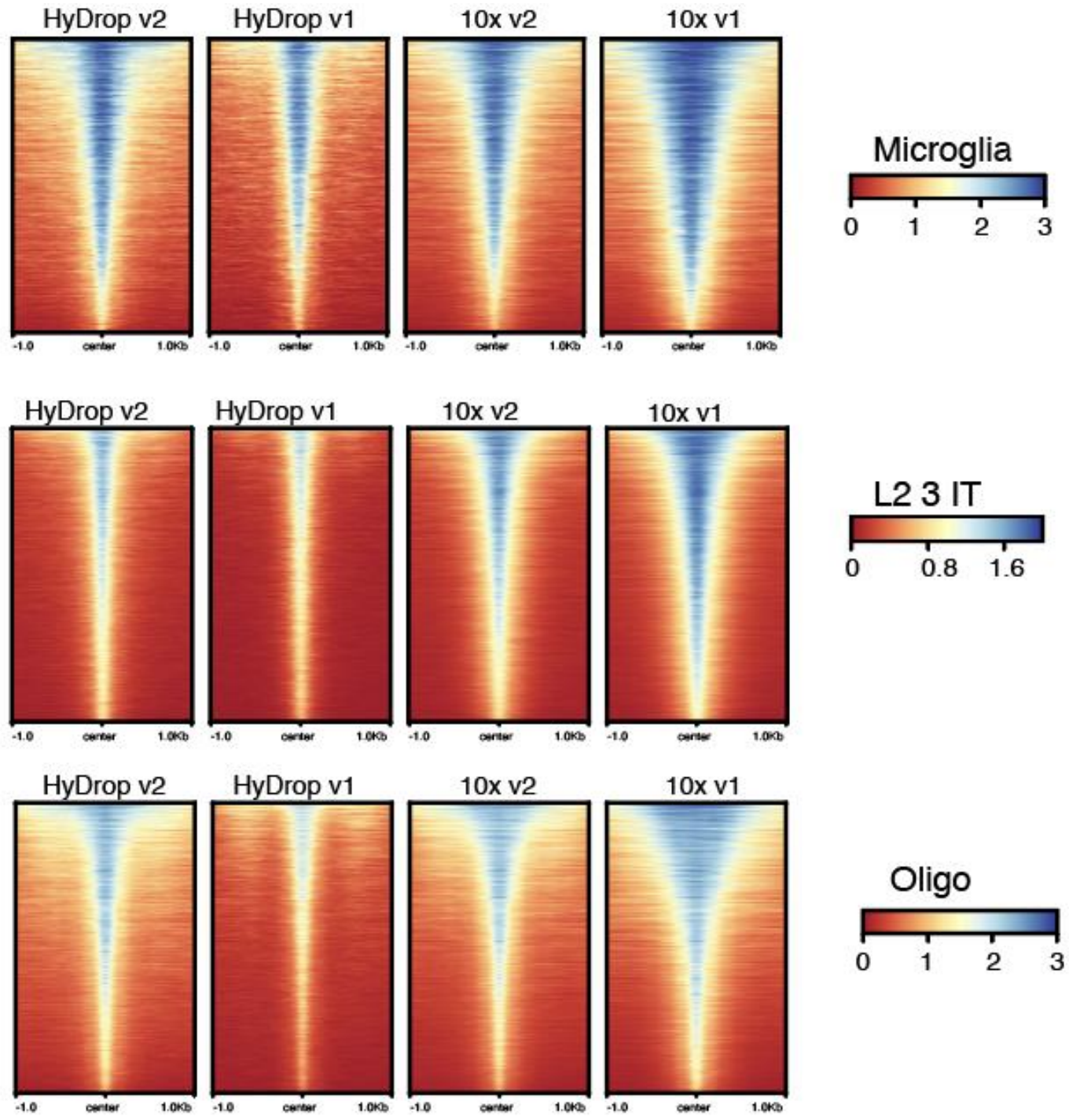

• For all plots with “percentage overlap of DARs” – it wasn’t clear to me what the percentage overlap is based on. Start and end sites of the peak? Signal in peak? Genes it’s annotated to? For 2d, in cell types with less strong concordance, is there are relationship with the number of cells captured (for example the L5_IT).

The percentage of overlap is defined as the same regions being identified as DAR in a given cell type in and 10x v2 and HyDrop v2 data, divided by the total number of DARs of that specific cell type. Due to the processing of the consensus peaks in pycisTopic, the regions are all 500 bp wide. We clarified this in the Methods section for both mouse and fly.

Figure 3:

- For clarity in reading, I think it could be useful to point out in the text how many nuclei/cells were used for the BICCN model using the 10x data to avoid having to cross-reference a lot of papers.

We agree, and as we abandoned the 10x data coming from the BICCN model, we clarified the number of cells used for the models in Tables S1 and S2 for mouse and S3 for fly.

- Possibly a rendering problem, but Figure 3f is difficult to read because of the color choice.

We regenerated this figure (Fig. S3a) with all models trained, keeping the color scheme in mind.

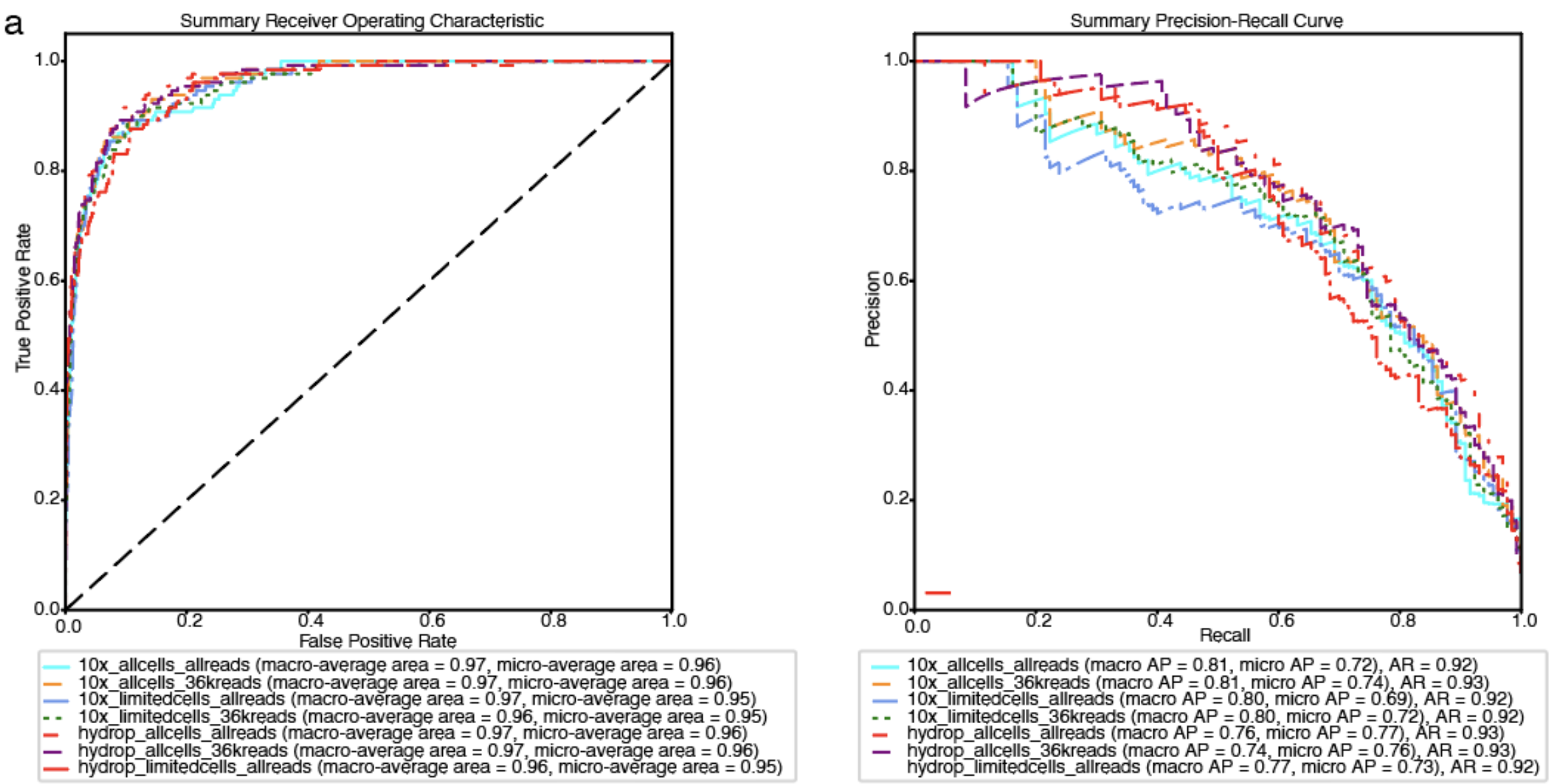

- Reference to panel 3g is missing from text, and from legend how to interpret the heatmap was not exactly clear. Presumably related to the predictions use to calculate the AP and related to 3f.

We clarified this in the current manuscript. Ben-Simon et al. (2024) published enhancer regions that were tested *in vivo*. We validated our model by investigating its prediction of these enhancer regions. We scored an enhancer as a true positive if the model predicts the region to be accessible.

- Possible edit for clarity: 3j is referenced in the main text in the context of the TF footprints, which are been assessed in the mouse and the Drosophila. I think thiis is referred two in the lines 268-271 which corresponds on the mouse section. Consider moving all the foot printing together?

We have moved the footprinting sections together as suggested. Additionally, we investigate the Tn5 bias between platforms, because seq2PRINT (Hu et al., 2025) assumes an equal Tn5 bias to be able to apply the footprinting model correctly.

Figure 4

- Consider adding Figure A from S5. This is the data that help us understand claims about the batch effects. Same comments as the mouse data apply here, except it's much easier to see as you have comparable numbers of nuclei. Since here you are really starting with the same samples, it would be possible to do an estimation of cell-type capture per technology.

In fly, no dissection is performed, and the whole animal is sequenced. All cell types are found by both techniques to a similar extent (here, the HyDrop v2 dataset includes overall only 27% more cells). The table below shows the cell count as well as the differences per cell type. We included the tables as S3 in the manuscript. We see a vast increase in neuronal cells in the HyDrop v2 dataset, as they are the largest cell group in the last stage embryo (Wang et al., 2025), leading to an overrepresentation when more cells are sampled. The tables is added as S3 to the manuscript.

**annotation**	**10x_count**	**HyDrop_v2_count**	**Dataset with more cells**	**Amount of additional cells in %**

**Epidermis**	40843	36833	10x_v2	10.89
**Fat_body**	17681	19985	HyDrop_v2	13.03
**Glia**	5372	5022	10x_v2	6.97
**Head_Ectoderm**	3117	4059	HyDrop_v2	30.22
**Hemocytes**	3808	3617	10x_v2	5.28
**Hindgut**	5582	6682	HyDrop_v2	19.71
**Malpighian_tubule**	3776	4904	HyDrop_v2	29.87
**Midgut**	32156	37442	HyDrop_v2	16.44
**Midgut_acidification**	2209	3283	HyDrop_v2	48.62
**NA_lowQ**	51090	77174	HyDrop_v2	51.06
**Neuroblasts**	6945	11134	HyDrop_v2	60.32
**Neuronal**	31561	61027	HyDrop_v2	93.36
**PNS_sens_neurons**	3876	7895	HyDrop_v2	103.69
**Pharnyx**	11747	10697	10x_v2	9.82
**Primordium_all**	6627	7770	HyDrop_v2	17.25
**Salivary_gland**	3853	5373	HyDrop_v2	39.45
**Somatic_muscles**	14431	15269	HyDrop_v2	5.81
**Tracheal_system**	6873	7504	HyDrop_v2	9.18

**Visceral_muscles**	5495	5803	HyDrop_v2	5.61
**Yolk**	3396	4063	HyDrop_v2	19.64
**muscle_attachement_Strip e**	6288	5068	10x_v2	24.07

- There is a change in the efficiency of the sequencing (i.e HyDropv2 needs more reads to get to the same depth). Within the error of the 10x this doesn't seem meaningful to me (and from a cost perspective is not important) but I am more curious is you have a sense of why. I ask this in the case of thinking about "non-ideal" tissues – i.e. postmortem samples from patients, surgical discard etc. where already the noise tends to be larger and the data quality decreases.

Indeed, there is a sequencing efficiency drop for HyDrop v2 compared to 10x. This might be due to a lower efficiency of ATAC fragment and barcode pairing in the custom setup. Even though we solved the bead impurities of HyDrop v1 to a large extent, some impurities may remain. Other factors might be the nuclear lysis and the oligo number released from the bead, both within in droplet. Comparing the effect of sequencing depth on S2F model performance, however, shows that downsampling to 36kRPC (mouse) only slightly alters model performance parameters while still being able to capture biologically relevant features in the form of *in vivo* validated enhancers. Importantly, our libraries appeared largely saturated at this depth, offering a possibility for cost reduction by providing a potential cutoff for sequencing depth when producing data for DL purposes. This negligible effect of sequencing depth in DL tools is in line with earlier findings showing that the number of DARs does not depend on sequencing depth if saturated (De Rop et al., 2023).
In regard to the patient samples, all our in-house generated samples (10x and HyDrop) were processed from frozen tissue partly stored >12 months, making the input material more comparable to stored post-mortem samples as well.

- The scoring of 4f is generally better than in the mouse, suggesting that really the differences are more experiment to experiment (as the mouse data was not collected at the same time as the mouse Hydrop) and highlights the concordance of the data nicely. There is about 80% overlap in the two technologies on average, which is great. Do the authors have a sense about what the 20% non-overlapping represents? Technical noise, differences in the Tn5 and other reagents etc?

Great, that is exactly what we wanted to discuss with the figure. Especially in mouse, the 20% might also be attributed to sampling-related differences due to the dissection (as discussed above).

In fly, sequencing depth can largely be excluded as a factor; an earlier publication (De Rop et al., 2023) finds that it does not affect the DAR selection as well as both datasets are sequenced comparably deep (10x v2: Mdn=13.21kRPC, SD=2.09; HyDrop v2: Mdn=15.88kRPC, SD=6.81). Additionally, we show that the Tn5 bias is comparable in both techniques. Thus, technical noise could be the source of the non-overlapping regions.

Figure 5:
- Are the 340,604 nuclei from 5A the same as from 4b? The difference essentially is the projection with or without 10x data in the same space?

Yes, Figure 5A only included the HyDrop data, as this is the main focus of that section, the tSNE of only 10x data can be found in supplement. In the current version of the manuscript, we adapted the figures and added a clearer explanation to the figure itself when only HyDrop data is included.

- From the lengend, a little hard to interpret the data related to the Kvon et al data (how is the ground truth in 5f scored?

5f merely takes the region (coordinates) from Kvon et al. (2014), while the ground truth is the accessibility in the actual HyDrop v2 data. We added this clarification to the figure legend.

- Line 445-446: "Possible variations between models can be attributed to model variability, as shown in the 10-fold cross-validation (Figure 5d)" – circular sentence; I think the authors means that models trained on both datasets have similar confidence intervals and thus accuracy ranges?

We would like to say "Possible variations between predictions can be attributed to model variability, as shown in the 10-fold cross-validation". This has been adapted in the manuscript.

Reviewer #4 (Remarks to the Author):

I co-reviewed this manuscript with one of the reviewers who provided the listed reports. This is part of the Nature Communications initiative to facilitate training in peer review and to provide appropriate recognition for Early Career Researchers who co-review manuscripts.
