# Supplementary material for: Evaluating single-cell ATAC-seq atlasing technologies using sequence-to-function modeling
Source: Nat Commun. 2026 Jan 22;17:1951. doi: 10.1038/s41467-026-68742-4 (PMC12929571; doi:10.1038/s41467-026-68742-4)
Supplement: Supplementary file 4 [file 41467_2026_68742_MOESM6_ESM.pdf]

# Supplementary material

## Supplementary note on HyDrop v2 technical improvements

The improvements on the HyDrop v1 platform, focusing on improved beads and protocol (Figs. 1c, d, and Methods), result in the new HyDrop v2 scATAC-seq platform (further addressed as HyDrop v2).

An earlier benchmarking[9] showed that the original HyDrop method suffered from lower sensitivity and showed a high variability in performance between bead batches. Therefore, we first modified the bead barcoding process.

The bead barcoding chemistry in the previous version of HyDrop was performed using a 3-stage extension of DNA primers grafted onto polyacrylamide gel beads that were generated using droplet microfluidics. Before each next stage of bead barcoding, the second strand was denatured, and beads were washed to remove the second strand (Fig. 1c). For details, see De Rop et al. (2022). In the current version of HyDrop v2 bead production, the polymerase-based cyclic extension of the barcoded primers was replaced by ligation of the barcoded primers. Barcoded oligo cassettes are now ligated sequentially in a 3-stage barcoding process, resulting in the same barcode complexity as the HyDrop v1 beads. Importantly, in the HyDrop v2 bead production, there is no intermediate strand denaturation or cyclic addition of primers to beads (Fig 1c), thereby enhancing the barcode stability (Figs. 1c and S1a). Single bead picking, followed by Sanger sequencing, confirmed increased purity of individual barcodes (Fig. 1e), leading to an increased sensitivity.

An additional quality challenge that we faced with HyDrop v1 bead production was the maintenance of stable bead quality, both in terms of the stability of the polyacrylamide gel bead matrix as well as in the maintenance of the size of the gel beads during bead production. Both factors are closely tied to the monomer composition of polyacrylamide chemistry. Through a series of optimization cycles, we adapted the monomer and crosslinker composition of the polyacrylamide chemistry from 8.5 %T and 4 %C in HyDrop v1 to 10 %T and 2 %C in HyDrop v2. This resulted in better hydrogel bead stability after a freeze-thaw cycle while retaining optimal dissolution times in the presence of DTT (Table in Fig S1b).

We also found that bead production using an in-house fabricated microfluidics droplet generator[17] outperformed commercial alternatives: a quality comparison of the hydrogel beads produced with commercial alternatives (droplet generator chips from Atrandi Biosciences) showed that the gel beads generated with the in-house fabricated bead generator yield beads of

superior quality. When using the Atrandi chip instead of the in-house (Fig. S1d) to generate the beads, we observed the flow and the droplet generation to be unstable, which resulted in a variable bead size and, more importantly, deformed beads after the –80°C storage. This is primarily attributed to the incompatibility of adding the N,N,N′,N′ -Tetramethylethylenediamine (TEMED), a polyacrylamide polymerization catalyst, during the bead emulsion generation with Atrandi's droplet generation chips. The deformability in beads was even more pronounced after freezing (Fig. S1e). In S1e, some beads appear brighter/more see-through and less spherical, which impairs the microfluidic flow during the experiment, causing unstable droplet generation and inefficient single cell encapsulation. When using the in-house bead generator, the beads had a very uniform size and varied minimally in size. With the optimized bead chemistry, we also observed no freezing artifacts (broken/deformed beads)..

The improvements in HyDrop v2 beads also led to an increased sequencing efficiency approaching the efficiency of 10x Genomics v1 ATAC chemistry (Fig. S1f). Despite the optimization of bead chemistry to improve the number of good-quality, valid barcodes, we are not yet at the level of 10x v2 ATAC chemistry, and this might be arising from the lower sequencing efficiency related to the downstream parts of the workflow, especially the enzymes and reaction buffers used in single indexing of the ATAC fragment. Suboptimality with this will directly affect the efficiency of ATAC fragments getting barcoded and, hence to the sequencing efficiency.
Next, HyDrop v2 and 10x atlases were generated for the mouse motor cortex (Fig. 1a) and the last four hours of *Drosophila melanogaster* embryo development (Fig. 1b). This embryo atlas represents the largest *Drosophila* embryo atlas of stage 17[30], a ten-fold increase compared to the stage 17 cells within the previously published time course of the *Drosophila* embryo[30] that was generated with sciATAC-seq3, a custom plate-based assay using combinatorial indexing[42]. A breakdown of the costs for bead generation and HyDrop v2 reagents can be found in Supplementary Tables S4 and S5, respectively. The total costs of the beads and ATAC reagents result in a price of approximately 100 euro for single nuclei sequencing of 10-12k cells.

After sequencing, for both species (mouse: HyDrop v1, v2, 10x v1, v2; fly: HyDrop v2, 10x v2), the fastq files were processed together using the PUMATAC pipeline (https://github.com/aertslab/PUMATAC), resulting in fragment files. These were then processed per species in pycisTopic[36]. For batch correction, harmony was performed with the pycisTopic[36] tool on the variable of the platform/experimental technique. It has been shown that this is effective in correcting for technical batch effects[9]. This straightforward processing facilitates the data generation (see Methods for details).

After processing, we compared the key quality metrics across platforms. Compared to the previously published HyDrop v1 atlas of the mouse cortex, we observe a 9.14% increase in the recovered unique fragments (log), now reaching 93.8% of the unique fragments (log) count of 10x v2-based data on average at a sequencing depth of 36k reads per cell (RPC, Fig. S2a).

Regarding the FRIP in mouse data, at 36kRPC, HyDrop v2 data reaches 97.01% (M=.5, SD=.09) of the FRIP of 10x v2 data (M=.53, SD=.09), while HyDrop v1 only reaches 81.8% (M=.43, SD=.1) of the 10x v2 FRIP score. The TSS enrichment in mouse at 36kRPC, is lower in HyDrop v2 (M=11.71, SD=5.3) than HyDrop v1 (M=13.73, SD=5.33) and 10x v2 (M=18.69, SD: 7.66) samples. Given the large standard deviation in data from all platforms, the TSS enrichment score seems to vary strongly across samples.

In the fly embryo atlas, HyDrop v2-based data also reaches an average of 94.1% of the unique fragments (log) count, compared to 10x v2-based data, both at 12kRPC (Fig. S2b). The number of unique fragments (log) is lower in sciATAC-based data (M=3.33, SD=.25) compared to HyDrop v2 (M=3.58, SD=.36) r 10x v2 (M=3.81, SD=.23). The FRIP scores are comparable across techniques (HyDrop v2: M=.55, SD=.14; 10x v2: M=.59, SD=.09; sciATAC: M=.6, SD=.08). The TSS enrichment of the plate-based sciATAC data is, however, lower with a mean of 2.73 (SD=.51) compared to 4.46 (SD=.95) in HyDrop v2 and 4.18 (SD=.81) in 10x v2. Importantly, the sequencing depth of the HyDrop v2 and 10x v2 data is downsampled to 12kRPC, while the sciATAC data was not downsampled, given the availability of the fragment files only.

To compare cell-type features in-depth, we calculated differentially accessible regions (DARs) of each cell type, per technique. Details on the DAR comparison and motif enrichment analysis can be found in the main text and Figures S2d-e.

As shown in the HyDrop v1 paper[17], the microfluidic setup is relatively straightforward, also when compared to other platforms mentioned in a recent scATAC platform benchmark[9]. We provide detailed protocols that make the generation of the beads and chips feasible, also for small labs. Additionally, plate-based assays are not always cheaper, as there are direct and indirect costs of automating the platforms, the laborious nature of the workflow, etc can add up to the costs of the assay[18]. As exemplified with the fly sciATAC data, there might appear additional quality issues that lead to strong batch effects we were not able to resolve. Integrating HyDrop v1, v2, and 10x v1, v2-based data, however, works well with a simple harmony batch correction in fly, mouse (Fig. S1h), and PBMC[9]. This makes HyDrop v2 a low-cost, high-throughput alternative that can be used for pilot experiments, protocol optimization, or generating whole atlases, of which the data can be integrated seamlessly with other microfluidic-based platforms. Important for application to stored samples, the current data generation was based on snap-

frozen tissue, achieving high-quality output data that can serve as training data for DL tools. Possible future optimizations for the HyDrop v2 platform include an extension to combinatorial indexing, as seen with OAK-seq[18] to achieve ultra-high throughput sample generation.

# Figures

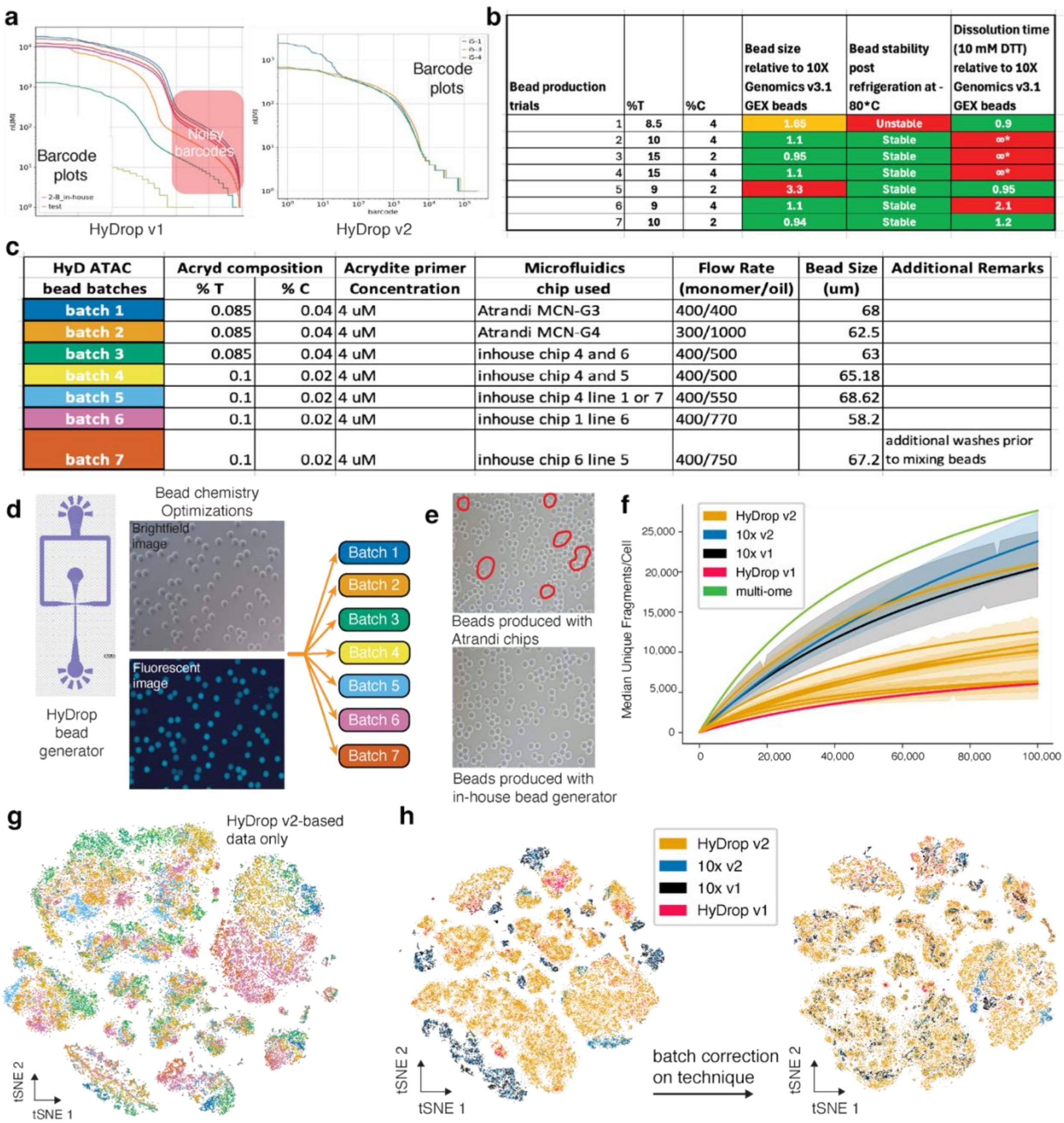


| Bead production trials | %T | %C | Bead size relative to 10X Genomics v3.1 GEX beads | Bead stability post refrigeration at -80*C | Dissolution time (10 mM DTT) relative to 10X Genomics v3.1 GEX beads |
|---|---|---|---|---|---|
| 1 | 8.5 | 4 | 1.65 | Unstable | 0.9 |
| 2 | 10 | 4 | 1.1 | Stable | ∞* |
| 3 | 15 | 2 | 0.95 | Stable | ∞* |
| 4 | 15 | 4 | 1.1 | Stable | ∞* |
| 5 | 9 | 2 | 3.3 | Stable | 0.95 |
| 6 | 9 | 4 | 1.1 | Stable | 2.1 |
| 7 | 10 | 2 | 0.94 | Stable | 1.2 |

| HyD ATAC bead batches | Acryd composition % T | % C | Acrydite primer Concentration | Microfluidics chip used | Flow Rate (monomer/oil) | Bead Size (um) | Additional Remarks |
|---|---|---|---|---|---|---|---|
| batch 1 | 0.085 | 0.04 | 4 uM | Atrandi MCN-G3 | 400/400 | 68 | |
| batch 2 | 0.085 | 0.04 | 4 uM | Atrandi MCN-G4 | 300/1000 | 62.5 | |
| batch 3 | 0.085 | 0.04 | 4 uM | inhouse chip 4 and 6 | 400/500 | 63 | |
| batch 4 | 0.1 | 0.02 | 4 uM | inhouse chip 4 and 5 | 400/500 | 65.18 | |
| batch 5 | 0.1 | 0.02 | 4 uM | inhouse chip 4 line 1 or 7 | 400/550 | 68.62 | |
| batch 6 | 0.1 | 0.02 | 4 uM | inhouse chip 1 line 6 | 400/770 | 58.2 | |
| batch 7 | 0.1 | 0.02 | 4 uM | inhouse chip 6 line 5 | 400/750 | 67.2 | additional washes prior to mixing beads |

**Fig S1|Improvement in HyDrop v2 bead generation. a**, Barcode ranking plot showing noisy barcode contamination in the HyDrop v1 beads arising from the barcode bleeding during the cyclic polymerase extension in the bead production. **b**, Characterization of bead quality and stability with different monomer compositions. Different percentages of total monomer and cross-linker composition are varied during different bead productions. The stability of the beads was evaluated using three different characteristics: i.) bead size variations, ii.) bead stability post-refrigeration, and iii.)

dissolution time of the beads in a reducing environment relative to 10X genomics NextGEM 3' RNA beads. **c**, Table illustrating different parameters and characteristics of different bead production batches. **d**, The design of the microfluidic chip to generate HyDrop hydrogel beads (left), alongside the image of the polymerized hydrogel bead and fluorescence image of the bead labeled with the complement of the Tn5 fragment capture site. Seven bead batches were produced. **e**, Bright field image of the beads showing freezing artifact (deformed beads, see-through beads as they are less solid) in suboptimal bead batch productions. **f**, Sequencing efficiency compared across 10x v1 and v2, multi-ome, HyDrop v1 and HyDrop v2 beads (7 batches). **g**, t-distributed stochastic neighbor embedding (tSNE) of all 67,080 cells generated with HyDrop v2 (35 experiments), colored by bead batch (seen in c), no batch correction is applied. Data points were randomly shuffled before plotting. **h**, *t*-distributed stochastic neighbor embedding (tSNE) of all 92,363 cells across 44 experiments, colored by technique used, left: no batch correction, right: batch corrected using Harmony for the used technique. Data points were randomly shuffled before plotting.

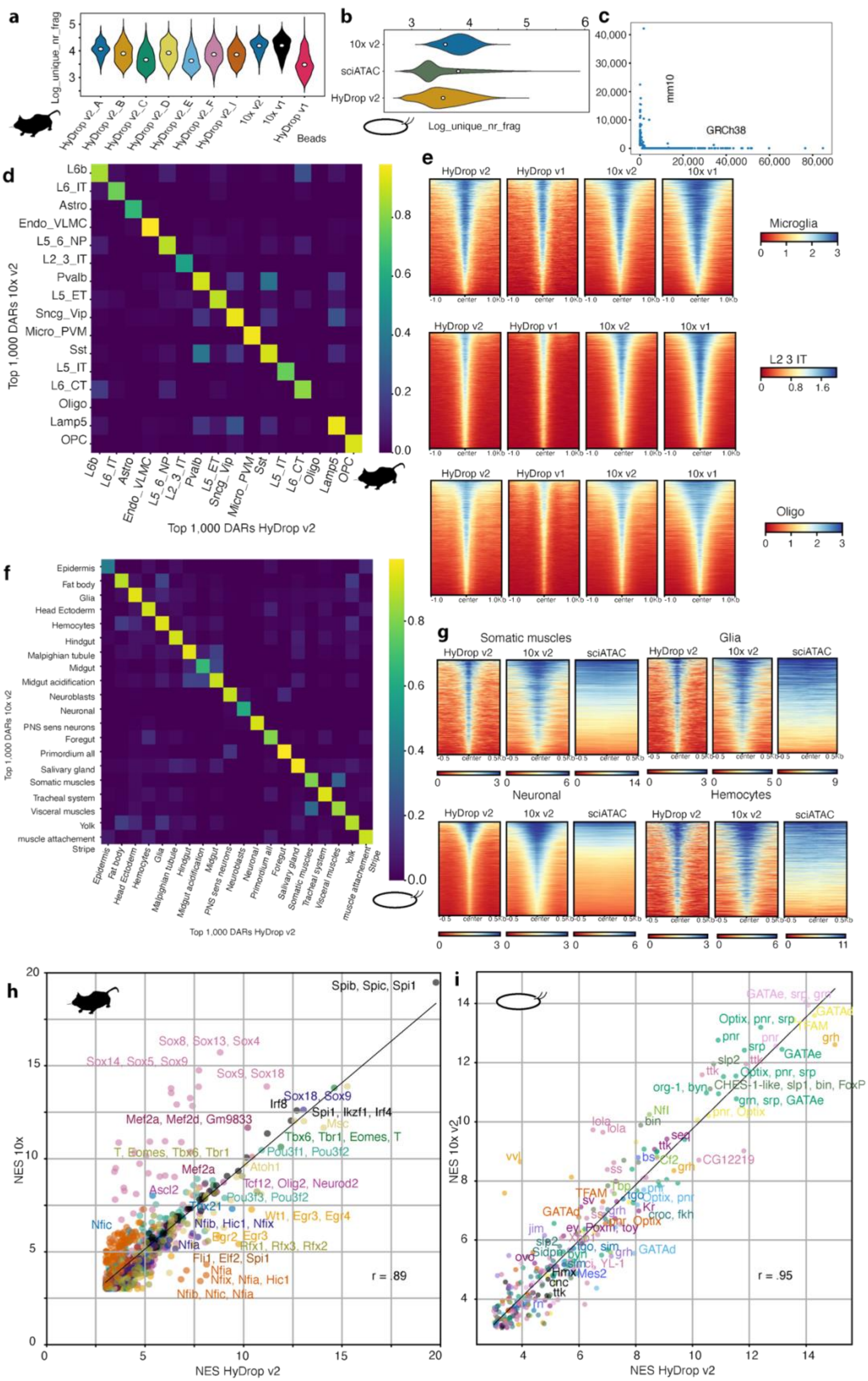
a
Log_unique_nr_frag
HyDrop v2_A
HyDrop v2_B
HyDrop v2_C
HyDrop v2_D
HyDrop v2_E
HyDrop v2_F
HyDrop v2_I
10x v2
10x v1
HyDrop v1
Beads
b
10x v2
sciATAC
HyDrop v2
Log_unique_nr_frag
c
mm10
GRCh38
d
Top 1,000 DARs 10x v2
Top 1,000 DARs HyDrop v2
L6b
L6_IT
Astro
Endo_VLMC
L5_6_NP
L2_3_IT
Pvalb
L5_ET
Sncg_Vip
Micro_PVM
Sst
L5_IT
L6_CT
Oligo
Lamp5
OPC
e
HyDrop v2
HyDrop v1
10x v2
10x v1
Microglia
L2 3 IT
Oligo
f
Top 1,000 DARs 10x v2
Top 1,000 DARs HyDrop v2
Epidermis
Fat body
Glia
Head Ectoderm
Hemocytes
Hindgut
Malpighian tubule
Midgut
Midgut acidification
Neuroblasts
Neuronal
PNS sens neurons
Foregut
Primordium all
Salivary gland
Somatic muscles
Tracheal system
Visceral muscles
Yolk
muscle attachement Stripe
g
Somatic muscles
Glia
Neuronal
Hemocytes
HyDrop v2
10x v2
sciATAC
h
NES 10x
NES HyDrop v2
Spib, Spic, Spi1
Sox8, Sox13, Sox4
Sox14, Sox5, Sox9
Sox9, Sox18
Sox18, Sox9
Irf8
Spi1, Ikzf1, Irf4
Mef2a, Mef2d, Gm9833
Tbx6, Tbr1, Eomes, T
T, Eomes, Tbx6, Tbr1
Pou3f1, Pou3f2
Atoh1
Mef2a
Tcf12, Olig2, Neurod2
Ascl2
Pou3f3, Pou3f2
Tbx21
Nfic
Wt1, Egr3, Egr4
Nfib, Hic1, Nfix
Nfia
Egr2, Egr3
Rfx1, Rfx3, Rfx2
Fli1, Elf2, Spi1
Nfia
Nfix, Nfia, Hic1
Nfib, Nfic, Nfia
r = .89
i
NES 10x v2
NES HyDrop v2
GATAe, srp, grn
GATAe
Optix, pnr, srp
TFAM
pnr
srp
grh
GATAe
slp2
ttk
Optix, pnr, srp
org-1, byn
CHES-1-like, slp1, bin, FoxP
grn, srp, GATAe
Nf1
bin
pnr, Optix
lola
lola
vvl
seq
ttk
bs
Cf2
CG12219
grh
ss
TFAM
sv
GATAd
Optix, pnr
Kr
croc, fkh
jim
ey
Poxm, toy
Optix
slp2
GATAd
ovo
r = .95

**Fig. S2|Quality metrics for mouse cortex and *Drosophila* embryo**. **a**, Mouse cortex: Count of unique fragments (log) for all cells sequenced at >36kRPC, for seven HyDrop v2 bead batches compared to 10x, and HyDrop v1 samples. The mean is indicated in white. **b,** Fly embryo: Count of unique fragments (log) for all cells sequenced at minimally 12kRPC recovered by experiments pooled per platform (10x v2, HyDrop v2, sciATAC). The mean is indicated per sample in white. **c**, HyDrop v2 barnyard with mouse and human cell line. Each dot represents cells assigned to genome. **d**, Mouse cortex: Heatmap of percental overlap of top 1000 DARs per cell type of 10x v2 and HyDrop v2 data. Source data are provided as a Source Data file. **e**, Mouse cortex: Heatmap of genome-wide regions of accessible chromatin ±1 kb around the center of DARs (logFC ≥ 1.5), sorted by the highest (blue) to lowest (red) accessibility for microglia, L2 3 IT neurons, and oligodendrocytes across the four different techniques. DARs are calculated on fragment coverage for each technique. **f**, Fly embryo: Heatmap of percental overlap of top 1,000 DARs per cell type of 10x v2 and HyDrop v2 data. **g**, Fly embryo: Heatmap of genome-wide regions of accessible chromatin ±0.5 kb around the center of DARs sorted by the highest (blue) to lowest (red) accessibility for neuronal and somatic muscle cells in 10x v2, HyDrop v2, and sciATAC data. DARs are calculated on fragment coverage for each technique. Source data are provided as a Source Data file. **h**, Mouse cortex: Scatterplot of normalized enrichment score of cell-type-specific transcription motifs, dots represent a common motif between HyDrop v2- and 10x-based data, colored per cell type as shown in Fig. 2. The labels are shown for the top three motifs per cell type **i**, Fly embryo: Scatterplot of normalized enrichment score of cell-type-specific transcription motifs, dots represent a common motif between HyDrop v2 and 10x v2 data colored per cell type as shown in Fig. 3. The labels are shown for the top three motifs per cell type. DAR: differentially accessible region, NES: normalized enrichment score.

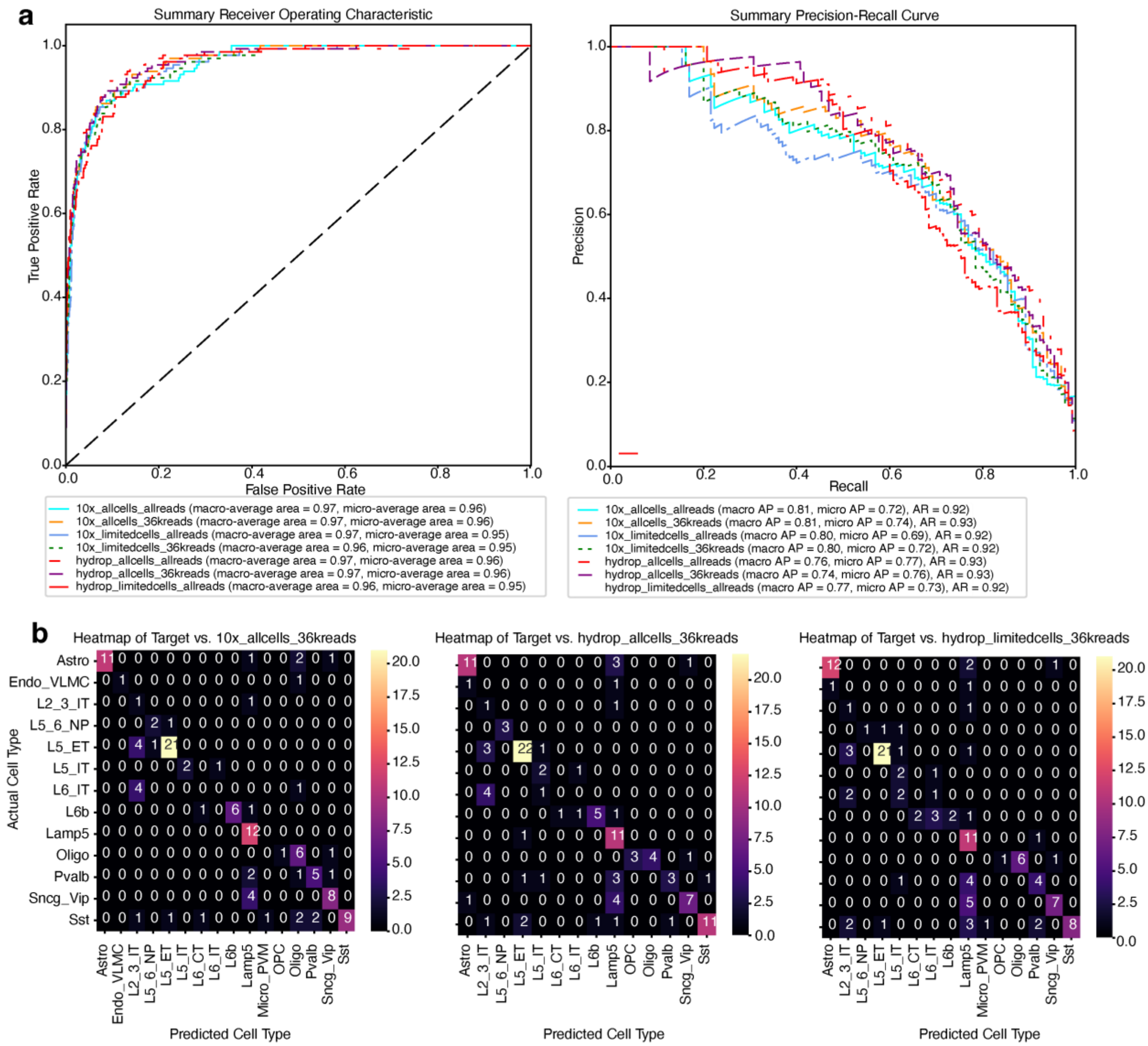


**Fig S3|S2F model performance in correctly identifying in vivo validated enhancers in the mouse motor cortex.** **a**, Summary receiver operating characteristics and specificity prediction for *in vivo* enhancer activity of validated mouse cortex enhancers by Ben-Simon et al. (2024) shown for the HyDrop v2-based and 10x-based models shown in Fig. 2b. **b**, Heatmap of *in vivo* validated enhancers (Ben-Simon et al., 2024) identified true positives by the 10x-based and HyDrop v2-based models. Source data are provided as a Source Data file.

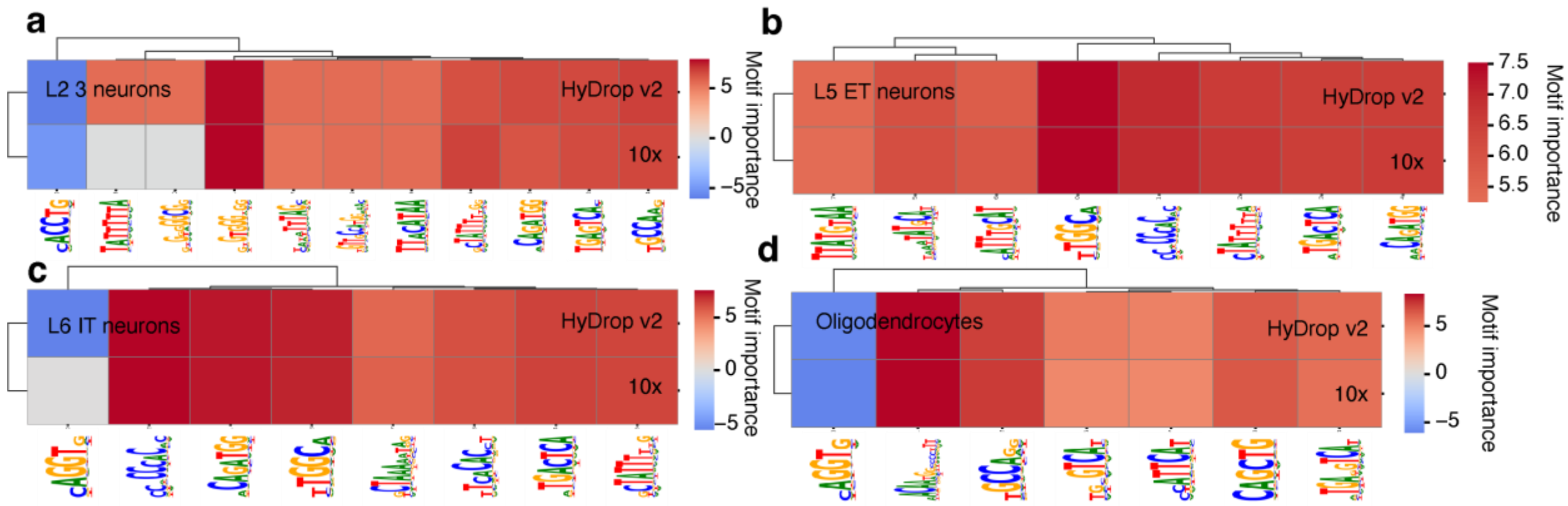


**Fig S4|Motif importance in the mouse motor cortex. a-d**, Evaluation of motif importance scores overlapping regions (present in HyDrop v2-based and 10x-based data) for cell type identity models trained on HyDrop v2 data- and 10x-based data for L2 3 (**a**), L5 ET (**b**), and L6 IT neurons (**c**), and oligodendrocytes (**d**).

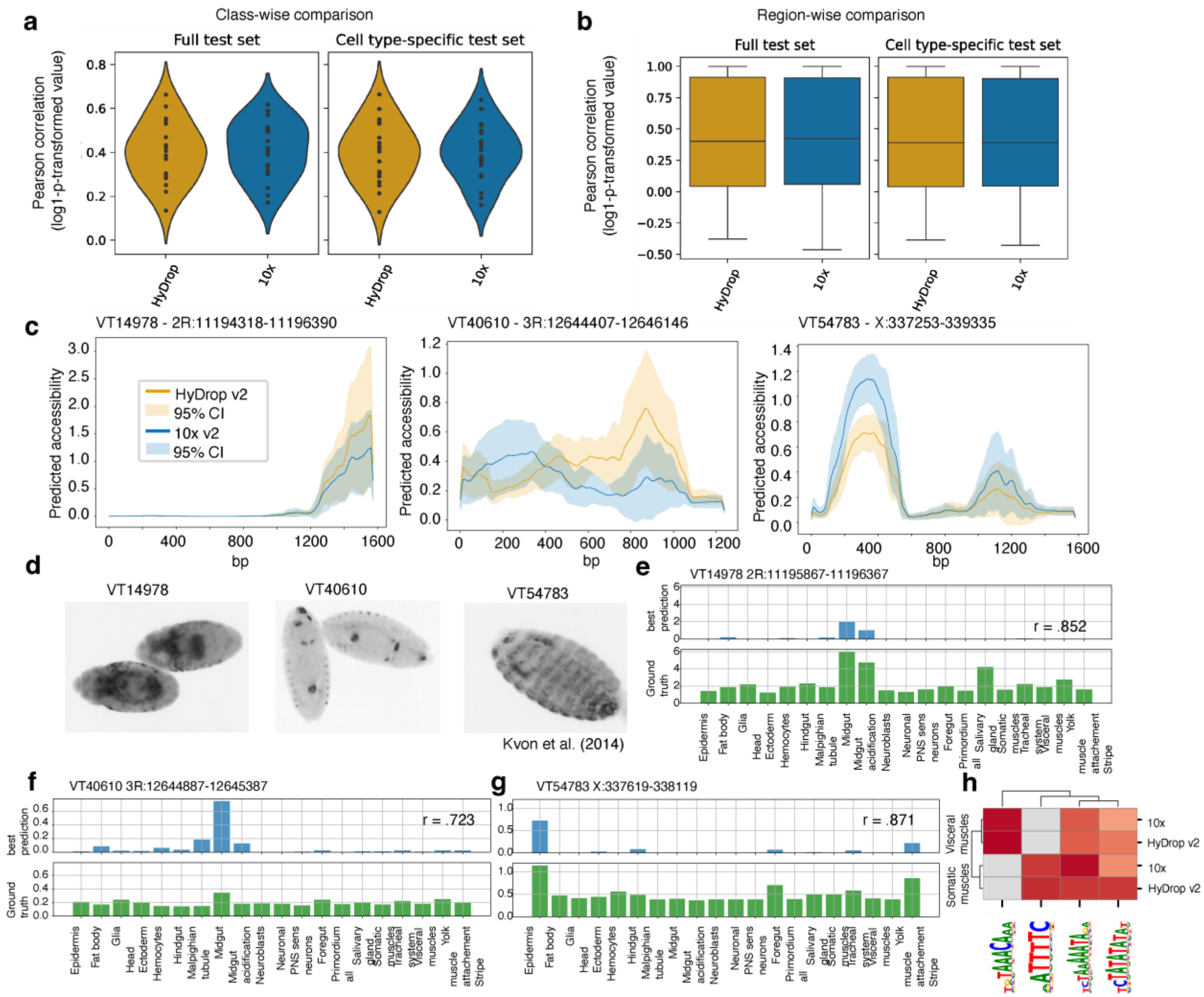


**Fig. S5|HyDrop v2-based sequence model matches results of 10x v2-based sequence model. a-b**, Model comparison of sequence models trained on HyDrop v2-based data (orange) and the 10x v2-based data set (blue). The accuracy of the predictions evaluated based on the ground truth of region accessibility is compared on class-wise (**a**) and region-wise level (**b**) for both the full test set in the data and cell type-specific test sets. **c**, Predicted accessibility of 500 bp windows of VT14978, VT40610, VT54783 enhancers from the VDRC library. Predictions based on the HyDrop v2 and 10x v2 data are shown confidence intervals reflect 10-fold cross-validation models. Line plots show mean predicted accessibility across n=10 cross-validation folds. Shaded bands represent 95% confidence intervals (1.96 × SD). **d**, *In situ* hybridization of VT14978-, VT40610-, or VT54783-Gal4 reporter embryos stage 15 with antisense Gal4 probe by Kvon et al. (2014). **e-g**, Prediction (top) versus ground truth (bottom) of 500bp region within 2kb long VDRC enhancers (VT14978, VT40610, VT54783). **h**, Evaluation of motif importance scores of highly relevant regions for cell type identity indicated by the 10x v2 data-based model and HyDrop v2 data-based model for visceral muscles compared to somatic muscles. An importance threshold of 5 was handled. VDRC: Vienna Drosophila Resource Center

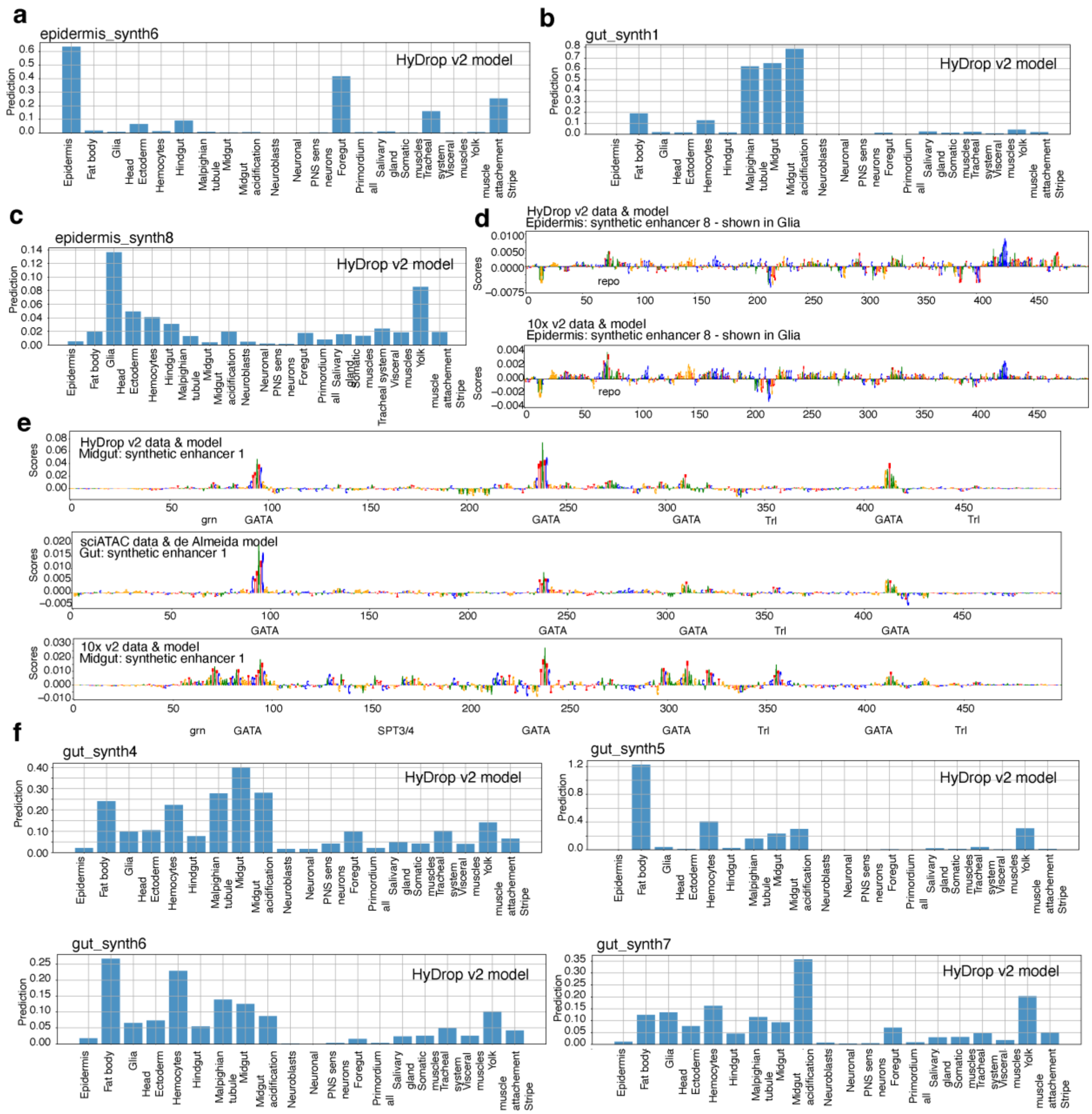

**Fig. S6|Evaluation of synthetic enhancers designed by de Almeida et al (2023) by the HyDrop v2-based model.** **a**, Prediction of tissue accessibility of a synthetic enhancer designed for epidermis cells in 10-12h embryos. Enhancers were designed by de Almeida et al. (2023) and scored with the HyDrop v2 model. Accessibility is specifically predicted for epidermis cells in the HyDrop v2 data. **b**, Prediction of tissue accessibility of a synthetic enhancer designed for gut cells in 10-12h embryos. Accessibility is specifically predicted for midgut cells in the HyDrop v2 data. **c**, Prediction of tissue accessibility of a synthetic enhancer designed for epidermis cells in 10-12h embryos. Accessibility is specifically predicted for glial cells in the HyDrop v2 data. **d**, Nucleotide contribution score of the 500 bp of synthetic enhancer designed to be active in epidermis cells based on sciATAC data by de Almeida et al. (2023): HyDrop v2 and 10x v2 model predictions shown for glial cells instead, and showing a *repo* motif. **e,** Nucleotide contribution score of the 500 bp of synthetic enhancer designed to be active in gut cells based on sciATAC data by de Almeida et al. (2023): HyDrop

v2 and 10x v2 model predictions shown for midgut cells versus predictions by the original model for general gut cells. **f**, Prediction of tissue accessibility of a synthetic enhancer designed for gut cells in 10-12h embryos. Enhancers were designed by de Almeida et al. (2023) but did not give a signal *in vivo*. Here, the synthetic sequences are scored with the HyDrop v2 model, showing predicted accessibility in tissues other than the gut.

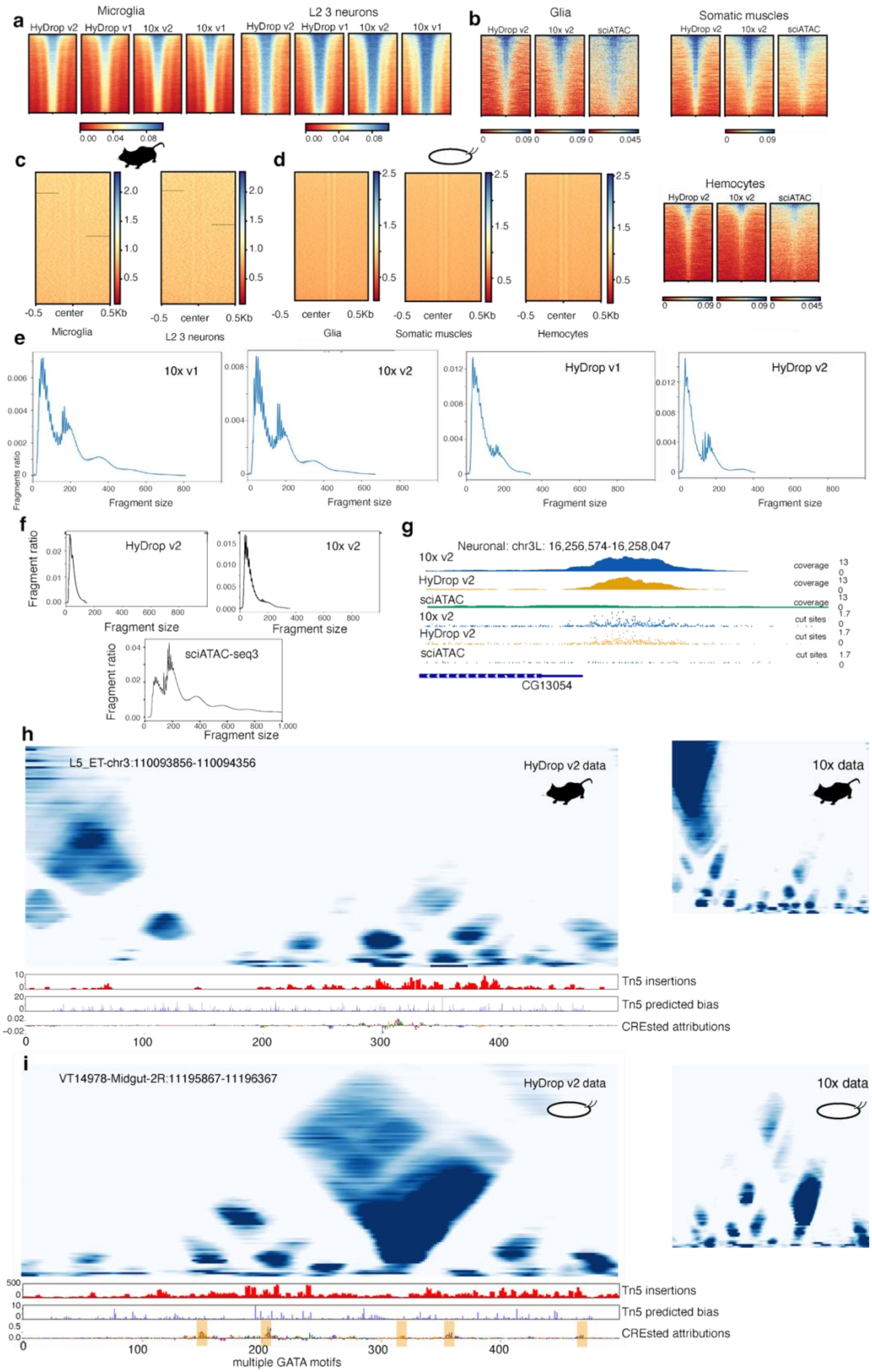
a
Microglia
HyDrop v2
HyDrop v1
10x v2
10x v1
L2 3 neurons
b
Glia
sciATAC
Somatic muscles
c
d
Hemocytes
e
10x v1
10x v2
HyDrop v1
HyDrop v2
Fragments ratio
Fragment size
f
Fragment ratio
sciATAC-seq3
g
Neuronal: chr3L: 16,256,574-16,258,047
coverage
cut sites
CG13054
h
L5_ET-chr3:110093856-110094356
HyDrop v2 data
10x data
Tn5 insertions
Tn5 predicted bias
CREsted attributions
i
VT14978-Midgut-2R:11195867-11196367
multiple GATA motifs

**Fig. S7|Similar Tn5 bias between droplet-based scATAC platforms results in comparable footprints using seq2PRINT pipeline. a-b**, Heat plot showing the regions of accessible chromatin ±0.5 kb around the center of DARs sorted by the highest (blue) to lowest (red) accessibility in for HyDrop v2-, HyDrop v1-, 10x v2-, and 10x v1-based data in mouse (**a**) and HyDrop v2-, 10x v2-, and sciATAC-based data in fly (**b**). **c**, Species-specific Tn5 bias from Hu et al., (2025) plotted on cell type-specific DARs. **e-f**, Fragment size plot of 10x v1, 10x v2, HyDrop v1, HyDrop v2 experiment in mouse (**e**) and HyDrop v2, 10x v2, and sciATAC-seq3 experiment in fly (**f**). **g**, Genome tracks (upper) and cut site insertion of Tn5 (lower) of cell-type specific DARs in somatic muscle cells down-sampled to the smallest count present for each technique (8,916 cells). The shown genome tracks are normalized for the fragment count. **h**, Multiscale footprint of the region (mm10 chr3:110093856-110094356) in L5 ET neurons from HyDrop v2-based data (left) and 10x-based data (right). Bottom tracks show the Tn5 insertion, the predicted Tn5 bias, and the nucleotide contribution scores based on the HyDrop v2-based S2F model. Previously described. **i**, Multiscale footprint of 500bp region in VT14978 in midgut cells from HyDrop v2-based data (left) and 10x-based data (right). The bottom track shows Tn5 insertion, the predicted Tn5 bias, and the nucleotide contribution scores based on the HyDrop v2 sequence model. DAR: Differentially accessible region, VDRC: Vienna Drosophila Resource Center, TF: Transcription Factor.

# Tables

**Table S1. Mouse M1 motor cortex cell count at full read depth**

| annotation | 10x_count | HyDrop_v2_count | Dataset with fewer cells |
|---|---|---|---|
| Astro | 2095 | 3807 | 10x |
| Endo_VLMC | 424 | 484 | 10x |
| L2_3_IT | 3189 | 9586 | 10x |
| L5_6_NP | 280 | 1156 | 10x |
| L5_ET | 523 | 2131 | 10x |
| L5_IT | 2814 | 8631 | 10x |
| L6_CT | 1643 | 5780 | 10x |
| L6_IT | 1563 | 5453 | 10x |
| L6b | 128 | 1041 | 10x |
| Lamp5 | 279 | 1121 | 10x |
| Micro_PVM | 1030 | 4229 | 10x |
| OPC | 911 | 2929 | 10x |
| Oligo | 3287 | 14550 | 10x |
| Pvalb | 580 | 3074 | 10x |
| Sncg_Vip | 368 | 1326 | 10x |
| Sst | 364 | 1782 | 10x |

**Table S2. Mouse M1 motor cortex cell count at 36kRPC**

| annotation | 10x_count | HyDrop_v2_count | Dataset with fewer cells |
|---|---|---|---|
| Astro | 2066 | 3332 | 10x |
| Endo_VLMC | 424 | 407 | HyDrop_v2 |
| L2_3_IT | 3188 | 8216 | 10x |
| L5_6_NP | 280 | 986 | 10x |
| L5_ET | 523 | 1771 | 10x |
| L5_IT | 2 813 | 7315 | 10x |
| L6_CT | 1642 | 4912 | 10x |
| L6_IT | 1562 | 4590 | 10x |
| L6b | 128 | 936 | 10x |
| Lamp5 | 279 | 940 | 10x |
| Micro_PVM | 1023 | 3679 | 10x |
| OPC | 904 | 2523 | 10x |
| Oligo | 3263 | 12524 | 10x |
| Pvalb | 580 | 2649 | 10x |
| Sncg_Vip | 368 | 1119 | 10x |
| Sst | 364 | 1514 | 10x |

**Table S3.** ***Drosophila*** **embryo cell count**

| annotation | 10x_count | HyDrop_v2_count | Dataset with more cells | Number of additional cells in % |
|---|---|---|---|---|
| **Epidermis** | 40843 | 36833 | 10x_v2 | 10.89 |
| **Fat_body** | 17681 | 19985 | HyDrop_v2 | 13.03 |
| **Glia** | 5372 | 5022 | 10x_v2 | 6.97 |
| **Head_Ectoderm** | 3117 | 4059 | HyDrop_v2 | 30.22 |
| **Hemocytes** | 3808 | 3617 | 10x_v2 | 5.28 |
| **Hindgut** | 5582 | 6682 | HyDrop_v2 | 19.71 |
| **Malpighian_tubule** | 3776 | 4904 | HyDrop_v2 | 29.87 |
| **Midgut** | 32156 | 37442 | HyDrop_v2 | 16.44 |
| **Midgut_acidification** | 2209 | 3283 | HyDrop_v2 | 48.62 |
| **NA_lowQ** | 51090 | 77174 | HyDrop_v2 | 51.06 |
| **Neuroblasts** | 6945 | 11134 | HyDrop_v2 | 60.32 |
| **Neuronal** | 31561 | 61027 | HyDrop_v2 | 93.36 |
| **PNS_sens_neurons** | 3876 | 7895 | HyDrop_v2 | 103.69 |
| **Pharnyx** | 11747 | 10697 | 10x_v2 | 9.82 |
| **Primordium_all** | 6627 | 7770 | HyDrop_v2 | 17.25 |
| **Salivary_gland** | 3853 | 5373 | HyDrop_v2 | 39.45 |
| **Somatic_muscles** | 14431 | 15269 | HyDrop_v2 | 5.81 |
| **Tracheal_system** | 6873 | 7504 | HyDrop_v2 | 9.18 |
| **Visceral_muscles** | 5495 | 5803 | HyDrop_v2 | 5.61 |
| **Yolk** | 3396 | 4063 | HyDrop_v2 | 19.64 |
| **muscle_attachement_Stripe** | 6288 | 5068 | 10x_v2 | 24.07 |

**Table S4. Cost breakdown of HyDrop v2 beads**

| **Amount in unit** | **Unit Price** | **Price** |
|---|---|---|
| | | |
| 400 | € 500.00 | € 55.00 |
| 100000 | € 100.00 | € 0.27 |
| 50000 | € 300.00 | € 0.29 |
| 25000 | € 65.00 | € 0.17 |
| 25000 | € 185.00 | € 0.07 |
| 50000 | € 800.00 | € 48.00 |
| | | € 15.00 |
| | | € 10.00 |
| 125000 | € 164.00 | € 3.94 |
| 250000 | € 77.00 | € 0.01 |
| 100000 | € 222.00 | € 5.99 |
| | | € 10.00 |
| | | **€ 148.74** |
| | | |
| | | |
| 7680 | € 1,132.35 | € 63.69 |
| 20 | € 1,800.00 | € 90.00 |
| 20 | € 1,800.00 | € 90.00 |
| 20 | € 800.00 | € 40.00 |
| 3 | € 912.00 | € 912.00 |
| 1000 | € 75.00 | € 121.19 |
| 50000 | € 88.00 | € 0.97 |
| | | € 80.00 |
| | | € 100.00 |
| | | **€ 1,497.85** |
| | | |
| 3000 uL of BHBs: | **Total** | **€ 1,646.59** |

Total usable for 60 reactions, resulting a cost per reaction of 27.44 euro.

## Table S5. Cost breakdown of HyDrop v2 reactions

| Item | Amount needed | Amount in unit | Unit Price | Price |
|---|---|---|---|---|
| **Enzymes** | | | | |
| **Tn5 (in-house synthesized)** | 3 | 170 | € 2,121.28 | € 37.43 |
| **Phusion High-Fidelity DNA Polymerase (2 U/µL)** | 1.6 | 250 | € 556.00 | € 3.56 |
| **Deep Vent** | 1.6 | 500 | € 511.00 | € 1.64 |
| **ET SSB** | 1.6 | 100 | € 196.00 | € 3.14 |
| **KAPA Master Mix** | 50 | 6250 | € 517.52 | € 4.14 |
| **Sub Total** | | | | € 49.90 |
| | | | | |
| **Microfluidic consumables** | | | | |
| **BioRad oil QX-200** | 200 | 70000 | € 270.00 | € 0.77 |
| **PDMS chip** | | | | € 5.00 |
| **Trichloro(1H,1H,2H,2H-perfluorooctyl)silane** | 1 | 7692 | € 88.00 | € 0.01 |
| **Sub Total** | | | | € 5.78 |
| | | | | |
| **Basic lab reagents** | | | | |
| **dH2O** | 200 | 1000000 | € 204.00 | € 0.04 |
| **Tris-HCl 7.4** | 15 | 1000000 | € 76.30 | € 0.00 |
| **MgCl2** | 5 | 250000 | € 100.30 | € 0.00 |
| **DMF** | 100 | 500000 | € 58.10 | € 0.01 |
| **Tween 20** | 11.5 | 100000 | € 24.20 | € 0.00 |
| **NP-40** | 0.5 | 50000 | € 72.20 | € 0.00 |
| **MgCl2** | 5 | 100000 | € 67.30 | € 0.00 |
| **Digitonin 2%** | 3 | 400 | € 278.00 | € 2.09 |
| **NaCl 5M** | 30 | 500000 | € 121.00 | € 0.01 |
| **DMF** | 100 | 250000 | € 41.00 | € 0.02 |
| **PBS** | 500 | 5000000 | € 33.00 | € 0.00 |
| **DTT** | 3 | 100000 | € 70.50 | € 0.00 |
| **dNTPs** | 8 | 1000 | € 286.00 | € 2.29 |
| **GITC** | 104.125 | 100000 | € 233.00 | € 0.24 |
| **EDTA** | 10 | 400000 | € 75.00 | € 0.00 |
| **MyOne Silane Dynabeads** | 10 | 5000 | € 488.00 | € 0.98 |
| **EtOH** | 2000 | 1000000 | € 10.00 | € 0.02 |
| **Ampure** | 200 | 500000 | € 2,120.00 | € 0.85 |
| **Other reagents and consumables** | | | € 10.00 | € 10.00 |
| **Sub Total** | | | | € 16.55 |
| **Cost of Beads** | | | | € 27.44 |
| | | | | |
| **Total cost per 10000 nuclei** | | | | € 99.68 |

The total costs of the beads and ATAC reagents results in a price of approximately 100 euro for single nuclei sequencing of 10-12k cells.
